# Supplementary material for: Superstructured mesocrystals through multiple inherent molecular interactions for highly reversible sodium ion batteries
Source: Sci Adv. 2021 Sep 8;7(37):eabh3482. doi: 10.1126/sciadv.abh3482 (PMC8442931; doi:10.1126/sciadv.abh3482)
Supplement: 20210908-1 [file sciadv.abh3482.v1.pdf]

APPLIED SCIENCES AND ENGINEERING

Superstructured mesocrystals through multiple inherent molecular interactions for highly reversible sodium ion batteries

Xiaoling Qiu[1†], Xiaoling Wang[1,2†], Yunxiang He[1†], Jieying Liang[3], Kang Liang[3], Blaise L. Tardy[4], Joseph J. Richardson[5], Ming Hu[6], Hao Wu[1]*, Yun Zhang[1]*, Orlando J. Rojas[7], Ian Manners[8], Junling Guo[1,2,9]*

Copyright © 2021 The Authors, some rights reserved; exclusive licensee American Association for the Advancement of Science. No claim to original U.S. Government Works. Distributed under a Creative Commons Attribution License 4.0 (CC BY).

Soft structures in nature, such as supercoiled DNA and proteins, can organize into complex hierarchical architectures through multiple noncovalent molecular interactions. Identifying new classes of natural building blocks capable of facilitating long-range hierarchical structuring has remained an elusive goal. We report the bottom-up synthesis of a hierarchical metal-phenolic mesocrystal where self-assembly proceeds on different length scales in a spatiotemporally controlled manner. Phenolic-based coordination complexes organize into supramolecular threads that assemble into tertiary nanoscale filaments, lastly packing into quaternary mesocrystals. The hierarchically ordered structures are preserved after thermal conversion into a metal-carbon hybrid framework and can impart outstanding performance to sodium ion batteries, which affords a capability of 72.5 milliampere hours per gram at an ultrahigh rate of 200 amperes per gram and a 90% capacity retention over 15,000 cycles at a current density of 5.0 amperes per gram. This hierarchical structuring of natural polyphenols is expected to find widespread applications.

INTRODUCTION

Hierarchical self-assembly with long-range spatial arrangement is ubiquitous in nature and is of widespread interest for engineering materials with enhanced properties (*1–5*) for applications in photonics (*6*), energy storage (*7*), drug delivery (*8*), gas adsorption (*9*), and catalysis (*10*). For example, hierarchical self-assembly via multiple noninterfering interactions on different length scales in a spatiotemporally controlled manner is the basis of various essential biological superstructures, such as supercoiled DNA (*11*), folded proteins (*12*), and biologically active cell membranes (*13*). Inspired by multiscale hierarchical structures in biology, substantial efforts have been devoted to developing synthesis pathways for similar intricate hierarchical systems including DNA origami construction (*14*), peptide-induced assembly (*15*), coordination-driven self-assembly (*16*), supramolecular polymerization (*17*), block copolymer assembly (*18*), and crystallization-driven self-assembly (*19*). Still, these synthetic approaches primarily rely on the same building blocks that biology already uses for assembling functional hierarchical structures (i.e., DNA and peptides) or rely on the elaborate design and fabrication of orthogonal units within synthetic organic ligands. Therefore, identifying new classes of simple, natural building blocks capable of facilitating long-range hierarchical structuring through multiple molecular interactions in a spatiotemporally controlled manner could shed light into the fundamentals of hierarchical self-assembly and potentially enhance the performance of the constituent building blocks for various applications.

Natural polyphenols, ubiquitous in plants and other organisms, are promising versatile "green" building blocks for engineering multifunctional supramolecular materials due to their ability to simultaneously exert multiple noncovalent interactions (*20–23*). For example, the catechol and galloyl moieties of many phenolic molecules can chelate metal ions and form hydrogen bond through their hydroxyl groups and can π-π stack and undergo hydrophobic interactions with their aromatic groups (*24–26*). Therefore, phenolic molecules offer a potential means to engineer hierarchical materials where different driving forces govern the self-assembly process on different length scales.

Here, we self-assembled a small planar natural phenolic molecule [ellagic acid (EA), a heterotetracyclic molecule found in fruits, vegetables, and tree bark] with bismuth ions (Bi^{3+}) into hierarchical metal-phenolic mesocrystals with ordered quaternary structure (Fig. 1, A to E). Small-angle x-ray scattering (SAXS) and computational thermochemistry modeling revealed a spatiotemporal hierarchical self-assembly process where coordination complexes (elementary structure) extended into supramolecular threads (secondary structure) directed by relatively strong π-π interactions between EA molecules form different complexes. The secondary threads then assembled into nanoscale filaments (tertiary structure) through dipole-dipole interaction between the bound solvent molecules, and lastly, the filaments packed into higher-order microscale mesocrystals via electrostatics (quaternary structure). These metal-phenolic mesocrystals could then be morphosynthetically transformed into Bi-carbon while still maintaining the hierarchically ordered structure, which allowed for their use as a high-performance anode material for sodium ion batteries (SIBs). In situ transmission electron microscopy (TEM) and in situ x-ray diffraction (XRD)

[1]BMI Center for Biomass Materials and Nanointerfaces, College of Biomass Science and Engineering, College of Materials Science and Engineering, Sichuan University, Chengdu Sichuan 610065, China. [2]Harvard John A. Paulson School of Engineering and Applied Sciences, Harvard University, Cambridge, MA 02138, USA. [3]School of Chemical Engineering, University of New South Wales, Sydney, New South Wales 2052, Australia. [4]Department of Bioproducts and Biosystems, School of Chemical Engineering, Aalto University, 02150 Espoo, Finland. [5]Department of Materials Engineering, School of Engineering, The University of Tokyo, Tokyo 113-8656, Japan. [6]School of Physics and Materials Science, East China Normal University, Shanghai 200241, China. [7]Bioproducts Institute, Departments of Chemical and Biological Engineering, Chemistry, and Wood Science, The University of British Columbia, Vancouver, BC, Canada. [8]Department of Chemistry, University of Victoria, Victoria, BC V8W 3V6, Canada. [9]State Key Laboratory of Polymer Materials Engineering, Sichuan University, Chengdu, Sichuan 610065, China.
*Corresponding author. Email: hao.wu@scu.edu.cn (H.W.); y_zhang@scu.edu.cn (Y.Z.); junling.guo@scu.edu.cn, junlingguo@g.harvard.edu (J.G.)
†These authors contributed equally to this work.

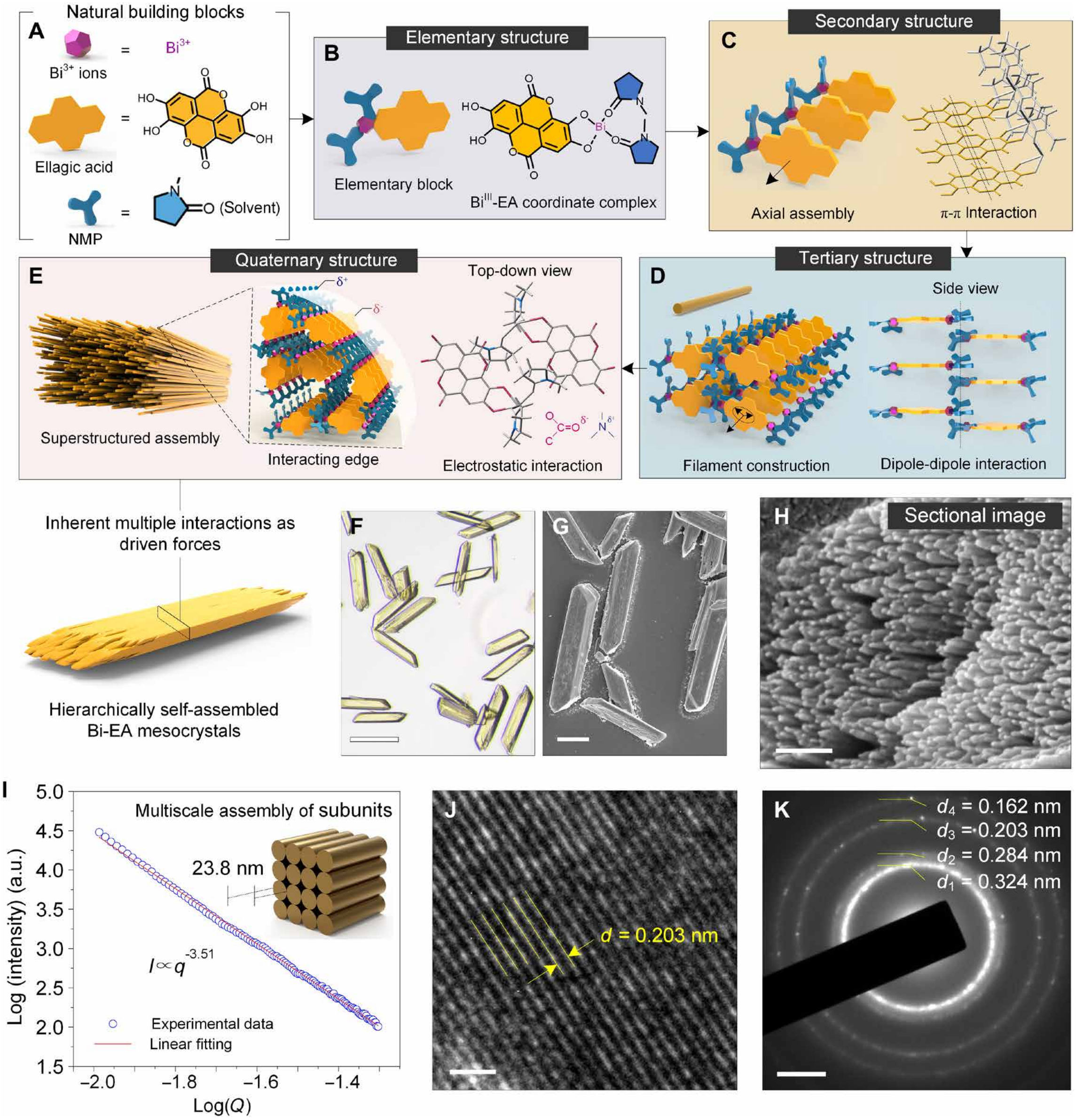

Fig. 1. Supramolecular hierarchal assembly of Bi-EA mesocrystals spatiotemporally driven by multiple inherent molecular interactions. (**A** to **E**) Schematic illustration of the hierarchical assembly of the structured Bi-EA mesocrystals driven by multiple molecular interactions on different length scales in a spatiotemporally controlled manner. Yellow color in (C) represented the structure of EA. Red and blue colors in (D and E) represented oxygen and nitrogen atoms, respectively. (**F**) Optical microscope image of Bi-EA mesocrystals. (**G**) SEM image of Bi-EA mesocrystals. (**H**) Magnified SEM image of a mesocrystal ends from (G). (**I**) Analyzed SAXS data of the Bi-EA mesocrystals and a schematic showing the multiscale structure of filaments to the mesocrystal. a.u., arbitrary unit. (**J** and **K**) TEM image and corresponding SAED pattern of a Bi-EA mesocrystal. Scale bars, 20 μm (F and G), 200 nm (H), 1 nm (J), and 2 1/nm (K).

techniques provided comprehensive insights into the electrochemical sodium storage mechanism and structural evolution process as well as the structural advantages of hierarchically ordered nanocomposites in facilitating Na^+ ions diffusion.

RESULTS AND DISCUSSION

Preparation and characterization of hierarchically structured Bi-EA mesocrystals

Bi-EA mesocrystals were prepared by simple mixing of EA and bismuth nitrate [$Bi(NO_3)_3$] in *N*-methyl-2-pyrrolidone (NMP) at room temperature. The complexation of EA and Bi^{3+} ions started as amorphous complexes, which leads to the formation of spherical disordered aggregates due to the rate-limiting step of addition of the last component in solution. The nanoscale aggregates sintered and ordered themselves upon equilibration toward the most stable conformation (i.e., Bi-EA mesocrystals) with aging. Scanning electron microscopy (SEM) showed a morphology transition from aggregated spherical particles to mesocrystals during a 7-day aging process (Fig. 1, F and G, and fig. S1, A and B). SEM images on the surface and ends of the mesocrystals revealed the subunit structures of aligned nanoscale filaments (tertiary structure) assembled into the

mesocrystals (Fig. 1H and fig. S1C). This subunit structure can also be supported from the height profiles obtained by atomic force microscopy (AFM) measurements. The formation of nanoscale two-dimensional (2D) sheets (height, ~4.2 nm) suggested the existence of subunits within the tertiary structure (fig. S2). SAXS data were fitted using a sphere model and indicated a radius of 23.8 nm of the filament section in the mesocrystals. A further Porod's law analysis, in which the log(I) versus log(q) plot showed a slope of −3.51, elucidated a fractal surface (Fig. 1I and fig. S3). Moreover, the clear fringes of high-resolution TEM image and the scattered ring pattern of selected-area electron diffraction (SAED) confirmed the crystallinity of the hierarchically structured mesocrystal particles (Fig. 1, J and K).

The elementary structure of the mesocrystals was the coordination complexes between EA and Bi^{3+} ions, where the extra positive charge was compensated by NMP molecules. Specifically, in the high-resolution x-ray photoelectron spectroscopy (XPS) spectra (Fig. 2A and fig. S4), a new peak for Bi—O bond (531.6 eV) in O 1s spectra and a peak for C—N bond (284.8 eV) in C 1s spectra, together with a peak shift of O—C, C═O, and C—C bond in C 1s spectra, confirmed the coordination among the three building blocks (*27*). Fourier transform (FT)–Raman spectra further supported the elementary coordination structures in the mesocrystals (*28*, *29*). The frequencies at 1748 and 1720 cm^{-1} were assigned to the carbonyl groups in NMP and EA. The sharp peaks at 1609, 1514, and 1499 cm^{-1} were assigned to the EA ring stretching. In the fingerprint region, the sharp peak at 1386 cm^{-1} was the characteristic frequency of the methyl group stretching in NMP. Lower intensity peaks at 396 and 414 cm^{-1} were observed for the Bi—O stretching between EA-Bi and NMP-Bi (Fig. 2B). Electrospray ionization mass spectrometry (ESI-MS) showed a fragment peak of 100.2, which provided clear evidence on the cocoordination of NMP in the elementary coordination structure (fig. S5).

To further understand the hierarchical and ordered features of the Bi-EA mesocrystals, we performed density functional theory (DFT) calculations on Bi-EA–NMP complexes under the B3LYP/6-311 theoretical model. The optimal coordination number of Bi^{3+} was first calculated with a model complex of $Bi(NMP)_n^{3+}$ (Fig. 2C). Because of strain and fast ligand dissociation of hexacoordinates $[Bi(NMP)_6^{3+}]$ during the calculation, five $[Bi(NMP)_5^{3+}]$ and four $[Bi(NMP)_4^{3+}]$ were adopted as the optimized coordination numbers for the complexes, as the results showed a similar stability (Gibbs free energy values). Tetracoordinate (−145.6 kcal mol^{-1}) and pentacoordinate (−163.4 kcal mol^{-1}) structures were selected as model complexes and applied directly to a reported unit cell of EA·H_2O for crystal structure simulation (Fig. 2D and fig. S6, A and B) (*30*). The calculated XRD patterns of tetracoordinate and pentacoordinate complexes correlated well with the experimental mesocrystal XRD pattern, which suggested the existence of at least two types of crystal units in

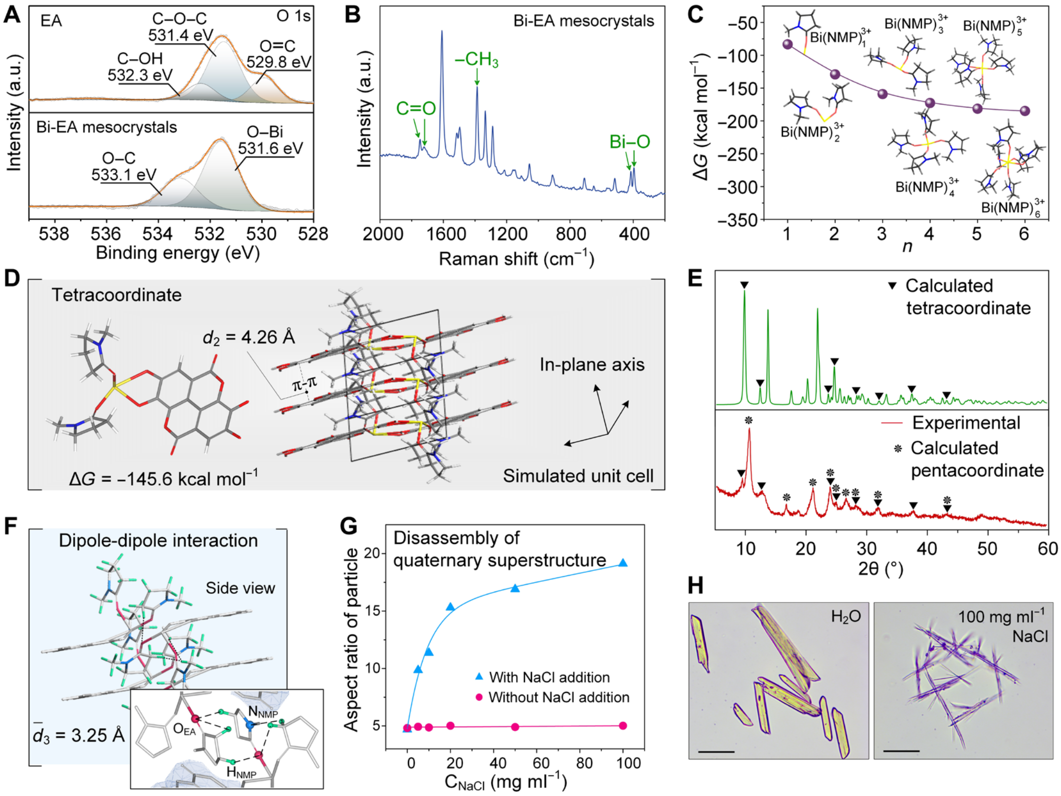

Fig. 2. Structural analysis of the multiscale structures in the Bi-EA mesocrystals. (**A**) High-resolution XPS spectra of O 1s for EA and Bi-EA mesocrystals. (**B**) Raman spectrum of Bi-EA mesocrystals. (**C**) Coordination number optimization based on a $Bi(NMP)_n^{3+}$ complex model. (**D**) Optimized tetracoordinate structure and corresponding simulated unit cell. (**E**) XRD patterns of Bi-EA mesocrystals (experimental) and those calculated for the optimal coordination numbers, namely, the tetracoordinate and the pentacoordinate complexes. (**F**) Side view and detailed molecular structure of the connection side of the supramolecular threads. (**G**) Disassembly of quaternary superstructure to nanoscale filaments as evidenced by the changes of aspect ratio of the superstructured mesocrystals with the addition of NaCl. (**H**) Morphological change of superstructured mesocrystals with the addition of NaCl. Scale bars, 20 μm (H).

the mesocrystals (Fig. 2E and fig. S6C). Note that from the modeling, the planar π-π interactions between EA layers along one out-of-plane axis (d_2 = 4.26 Å) are favored over the dipole-dipole interaction of bound NMP in the two perpendicular axes (average d_3 = 3.25 Å) (Fig. 2F).

The presence of π-π interactions in the hierarchal assemblies is also supported by the bathochromic shifts in the ultraviolet-visible (UV-Vis) and fluorescence emission spectra of the Bi-EA mesocrystals when compared to those of EA molecule and nonstructured Bi-EA complexes (fig. S7) (*31*, *32*). It can therefore be rationalized that the coordination complexes can be extended into the secondary structure of supramolecular threads through relatively strong π-π interactions and then assembled into the tertiary structure of nanoscale filaments through dipole-dipole interactions. Still, the final dominant interaction leading to the packing of the filaments into superstructured mesocrystals was determined to be electrostatics, as high molar NaCl was the only solvent that could disassemble the mesocrystals into nanoscale filaments, likely due to the screening of electrostatic interactions (Fig. 2, G and H, and fig. S8). Both SEM and AFM demonstrated that the Bi-EA mesocrystals disassembled into needle-like crystals after NaCl treatment (fig. S8). Moreover, no change in the Bi^{3+} ion content was observed by inductively coupled plasma optical emission spectrometer (ICP-OES), which suggested that the Bi^{3+} ions were not readily be replaced by Na^+ ions (table S1). Specifically, urea and Tween 80 solvents used for breaking hydrogen and hydrophobic bonds, respectively, did not lead to disassembly of the mesocrystals (fig. S9, B and C). Different cations and solvents were also examined for the preparation of metal-phenolic mesocrystals, including Fe^{3+}, Al^{3+}, Cu^{2+}, and Co^{2+} ions and ethanol (EtOH), ethyl acetate (EtOAc), toluene, and *N*,*N*-dimethyl formamide (DMF); however, no mesocrystal particles were observed for any of these experiments (fig. S10). Collectively, these results confirm the unique assembly mechanism and preparation condition of Bi-EA mesocrystals and the importance of using NMP as the solvent.

Morphological transformation of hierarchical Bi-EA mesocrystals

The ordered structure could be preserved after carbonization of the Bi-EA mesocrystals into a hierarchical Bi-carbon hybrid (HBiC) under inert atmosphere at 800°C for 2 hours (Fig. 3A and fig. S11). The HBiC contained uniformly distributed ultrafine Bi nanoparticles (about 9.8 nm in size) (Fig. 3B and fig. S12). The SAED diffraction rings of HBiC revealed the formation of metallic state of Bi in the nanostructured carbon matrix of HBiC (Fig. 3C). Energy-dispersive

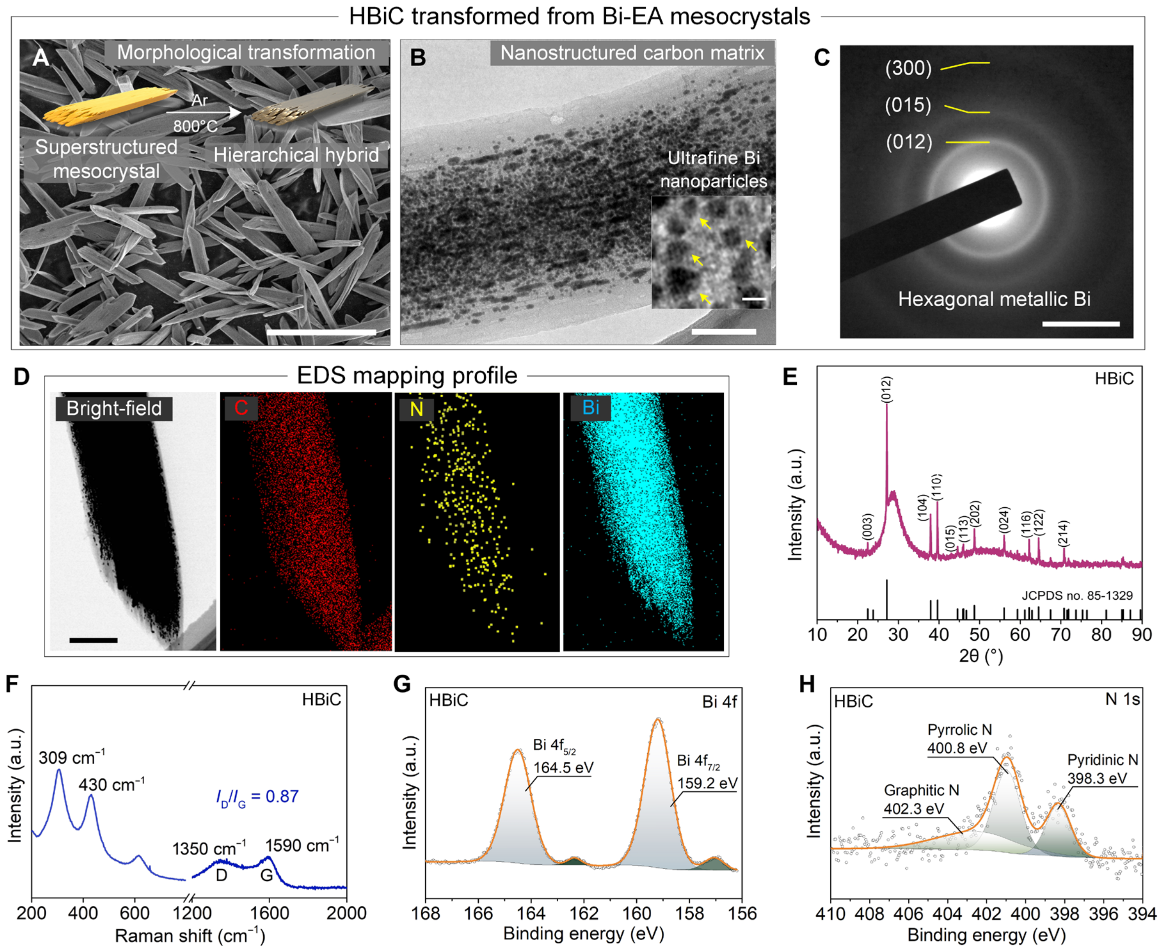

Fig. 3. Morphology and physical characterization of the HBiC. (**A**) SEM image of HBiC. (**B**) TEM images of HBiC. (**C**) SAED pattern of HBiC [inset of (B)]. (**D**) TEM image and corresponding EDS elemental mapping of HBiC. (**E**) XRD pattern of HBiC composite. JCPDS, Joint Committee on Powder Diffraction Standards. (**F**) Raman spectrum of HBiC. (**G** and **H**) XPS spectra of Bi 4f and N 1s of the HBiC. Scale bars, 20 μm (A), 200 nm (B), 5 1/nm (C), 500 nm (D), and 20 nm [inset of (B)].

x-ray spectroscopy (EDS) mapping profile showed the dense distribution of Bi element in the HBiC materials (Fig. 3D). In addition, the XRD pattern of HBiC also presented sharp peaks assigned to the hexagonal Bi phase (Fig. 3E). Specifically, the Bi content in the composite was calculated to be ~67% (fig. S13 and table S2), and its specific surface area was increased from 10.7 to 91.6 m^2 g^{-1} due to the graphitization of the supramolecular structures (fig. S14). The isotherm curves of the Bi-EA mesocrystals and HBiC were both type I (typical feature of microporous structure) and type IV (characteristic of mesoporous materials), indicating the existences of both micropores and mesopores in the Bi-EA mesocrystals and HBiC. The micropores, mesopores, and macropores contained in HBiC could increase the contact areas between the HBiC electrode and electrolyte, which further shorten the diffusion distance of Na^+ ions and facilitate their transportation (table S3). The peaks of Raman spectrum centered at 1350 and 1590 cm^{-1} were assigned to the D and G bands, respectively, and the corresponding I_D/I_G value of 0.87 implied a partially graphitized N-doped carbon matrix with high conductivity (Fig. 3F) (*33*, *34*). The main peaks of Bi 4f spectrum in XPS (at 159.2 and 164.5 eV) were ascribed to metallic Bi (Fig. 3G). The N 1s spectrum of XPS was deconvoluted into pyridinic N (at 398.32 eV, 31.64%), pyrrolic N (at 400.88 eV, 47.26%), and graphitic N (at 402.38 eV, 21.12%), respectively (Fig. 3H and fig. S15).

The chemistry and morphology of the HBiC composite suggest a promising candidate as anode for SIBs, which is given by the expected efficient cost, nontoxicity, and sustainability of the naturally abundant starting materials. Moreover, the nanostructured carbon matrix could lead to more favorable contact with the electrode-electrolyte interface and provide more accessible sodium storage sites. The Bi nanoparticles are promising components of the composite, due to the large lattice fringes of Bi along the *c* axis [$d_{(003)}$ = 3.95 Å] for Na^+ insertion. Last, such a N-doped carbon matrix should be conducive to enriching Na^+ trapping, thus enhancing the sodium storage capabilities.

Electrochemical testing of HBiC

Cyclic voltammetry (CV) tests with HBiC at a scan rate of 0.1 mV s^{-1} in the voltage window of 0.01 to 1.5 V (versus Na/Na^+) were first carried out to investigate the electrochemical reactions of Na_3Bi ↔ NaBi ↔ Bi (fig. S16), which was consistent with the results of charge-discharge plateaus (fig. S17) (*35*, *36*). The initial discharge and charge capacities were 449 and 359 mA·hour g^{-1}, respectively, with a high initial Coulombic efficiency of 79.9%, which was superior to recent reports on alloying-type anode batteries (*27*, *37*–*39*). At relatively low current densities (1.0, 2.0, and 10 A g^{-1}), the average rate capacity reached 317, 315, and 297 mA·hour g^{-1}, respectively (Fig. 4A and fig. S18). The capacity of the HBiC was maintained at 263 mA·hour g^{-1} (83% capacity retention versus 317 mA·hour g^{-1} at 1.0 A g^{-1}), even with a sharp increase in the testing current to 50 A g^{-1}. At a 100 A g^{-1} discharge-charge rate (6.5 s to full charge or discharge), more than 210 mA·hour g^{-1} was achieved, while the ultrahigh charge-discharge rate of 200 A g^{-1} (0.43 s to total charge or discharge) led to a capacity of 72.5 mA·hour g^{-1}. While lacking hierarchically ordered structure (fig. S19, A to D), the carbon sheet–based Bi materials (Bi-C) showed low-rate capacities at various current densities and poor cycling stability (fig. S19, L and M). Although the carbon nanotube–based Bi materials (Bi-CNTs) presented some ordering, derived from the assembled CNTs (fig. S19, E to H), its rate capacities at various current densities and cycling stability were also observed to be inferior (fig. S19, L and M), suggesting that the multiscaled porosity of HBiC and uniformly distributed ultrafine Bi nanoparticles were additionally important. Full recovery of the capacity (318 mA·hour g^{-1}) was obtained with the subsequently reducing test current to 1.0 A g^{-1}, which suggested an excellent tolerance for rapid Na^+ ions insertion-extraction reactions derived from the hierarchical structure of HBiC. However, when the Bi content was reduced to 46.7%, the rate capabilities of HBiC-1 were inferior with the increased current densities, lastly reaching zero at a current of 50 A g^{-1} (fig. S20). The capacity of HBiC-4 with a higher Bi content (78.9%) also decayed to zero, as the current density exceeded 100 A g^{-1}, although its capacity was higher to 550.2 mA·hour g^{-1} than that of HBiC (540.1 mA·hour g^{-1}) at the low current density of 0.1 A g^{-1}. The rate performance of the HBiC, especially at current densities exceeding 100 A g^{-1}, is the highest ever reported for Bi-based anode materials in SIBs (Fig. 4B and table S4).

The redox process in CV curve of HBiC showed fast reaction kinetics and depended on the diffusion of Na^+ ions (fig. S21). The average diffusion coefficient of Na^+ ions was calculated as 1.36 × 10^{-9} cm^{-1} s^{-1} (figs. S22 and S23), thus delivering into this high charge-discharge rate capability. According to DFT calculations, the Na^+ adsorption was modeled on the (001) crystal plane of Bi, which was the most readily exposed to the real environment, for the lowest surface energy (0.18 J m^{-2}) (table S5 and fig. S24), where we selected two stable sites (S1 site and S2 site) (fig. S25). The Na^+ preferred to migrate from an S1 site to an S2 site for a low adsorption energy of −0.67 eV and further to another S1 site (Fig. 4C and table S6). The corresponding diffusion energy barrier was 0.35 eV (fig. S26), which was low enough to enable fast ionic diffusion within layered Bi at room temperature (*40*). Thus, at the current densities switched between 0.2 and 2.0 A g^{-1}, the capacities remained at 329 and 337 mA·hour g^{-1}, respectively, without capacity fading after 160 cycles (fig. S27). Moreover, the charge-discharge voltage profiles had overlapped over 100 cycles, indicating the stability and reversibility of the HBiC (fig. S28).

An ultralong cycling performance of HBiC was also achieved with a high-capacity retention of 263 mA·hour g^{-1} over 15,000 cycles at a high current density of 5.0 A g^{-1} (90.6% of capacity retention) (Fig. 4D). The HBiC exhibited remarkable cycling stability, retaining a capacity of 325 mA·hour g^{-1} at a current density of 1.0 A g^{-1} after 1000 cycles (fig. S29). Furthermore, a high capacity of 302 mA·hour g^{-1} over 1700 cycles at 2.0 A g^{-1} was also tested. This outstanding stability could be ascribed to the fact that the hierarchically ordered structure of the HBiC could potentially relieve the stresses resulting from swelling during the alloying process to facilitate rapid Na^+ ions diffusion, thus delivering a high sodium ion storage performance (fig. S30). The HBiC presented a high reversible capacity of 306.9 mA·hour g^{-1} after 1035 cycles at 5.0 A g^{-1} with a mass loading of 2.72 mg cm^{-2} (fig. S31). At mass loadings of 3.89 and 6.08 mg cm^{-2}, the corresponding capacities were still maintained at 307.6 and 301.7 mA·hour g^{-1} after 450 cycles at 1.0 A g^{-1}, respectively (fig. S32). Even at a high mass loading of 9.35 mg cm^{-2}, a capacity of 284 mA·hour g^{-1} was observed after 450 cycles, corresponding to a reversible areal specific capacity of 2.66 mA·hour cm^{-2}. This is fundamentally comparable to commercial lithium ion battery anodes as electrodes in commercial cells, which commonly show an areal specific capacity of 2.5 to 3.5 mA·hour cm^{-2} and a mass loading of 9.0 to 9.5 mg cm^{-2} (*41*).

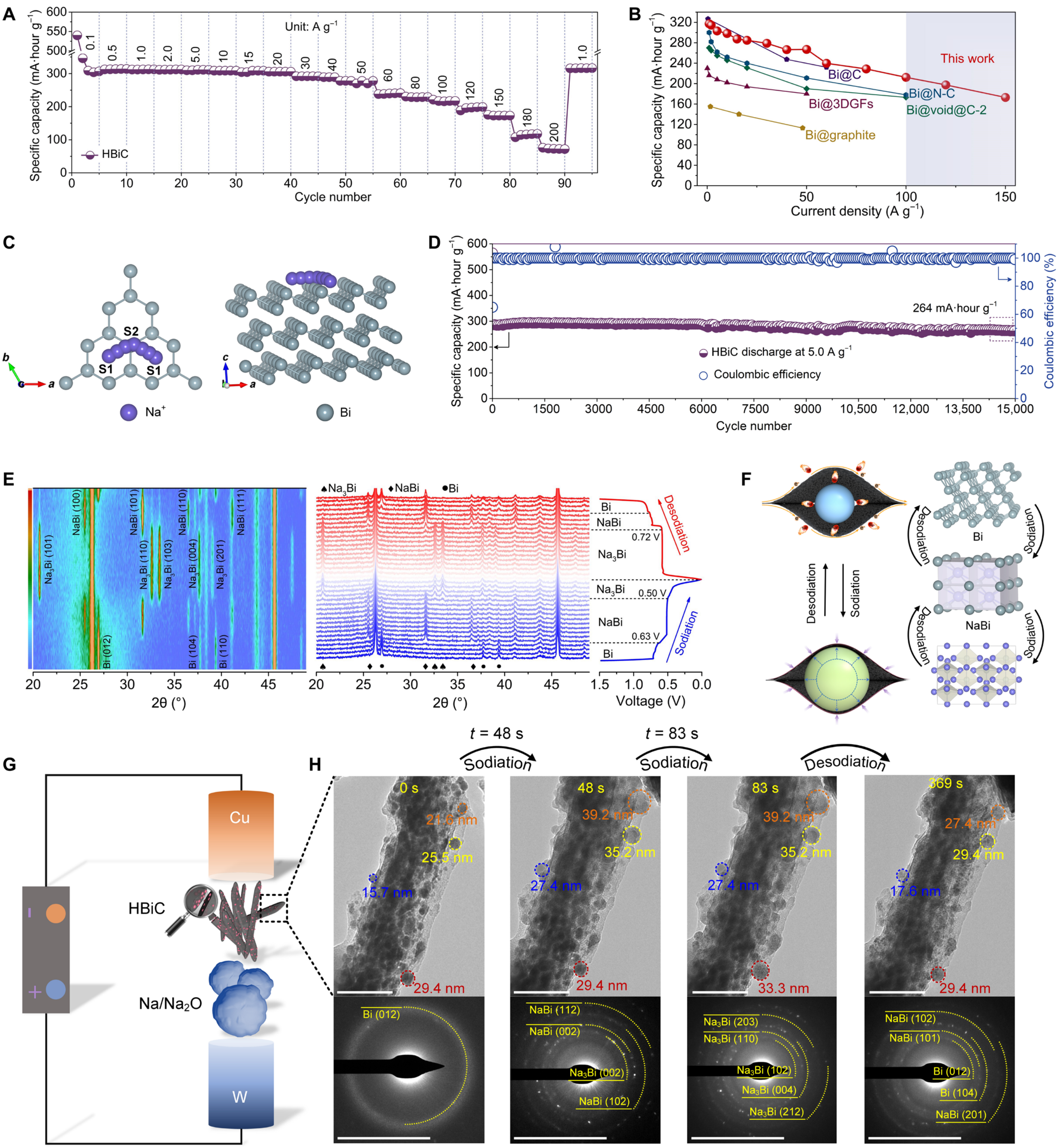

Fig. 4. Electrochemical performance and sodium storage mechanism of HBiC as an anode material. (**A**) Rate capabilities of HBiC at 0.1, 0.5, 1.0, 2.0, 5.0, 10, 15, 20, 30, 40, 50, 60, 80, 100, 120, 150, 180, 200, and 1.0 A g^{-1}. (**B**) Comparison of rate capability of HBiC with several reported Bi-based anode materials for SIBs. (**C**) Schematic illustration of the selected diffusion pathway for Na^+ adsorption and diffusion in Bi metal. (**D**) Specific capacity and Coulombic efficiency of HBiC after 15,000 cycles at 5.0 A g^{-1}. (**E**) In situ XRD patterns of the HBiC during the initial cycle. (**F**) Schematic illustration of the HBiC anode and corresponding phase transformation during sodiation-desodiation process. (**G**) Schematic illustration of the in situ TEM nanobattery setup. (**H**) Time-resolved TEM images and corresponding SAED patterns of HBiC. Scale bars, 100 nm and 5 1/nm, respectively (H).

In situ XRD was used to elucidate the reaction mechanism and phase evaluation during the first sodiation-desodiation process (Fig. 4E). The characteristic diffraction peaks in the contour plot represented the (012), (104), and (110) planes of Bi in the voltage window of 1.5 to 0.63 V. In the first voltage platform of 0.63 to 0.50 V, the peaks of Bi were gradually faded, accompanied by the emergence of new peaks at the (100), (101), and (110) planes of NaBi formed by the alloying reaction between Bi and Na. During the discharge process, the peaks of the (100), (101), (110), and (111) planes of Na_3Bi evolved in the voltage window of 0.50 to 0.01 V. These results demonstrated that Bi was first alloyed via sodiation to NaBi and then to Na_3Bi, which was consistent with our CV results. During the charge process, the XRD pattern also verified the gradual reduction of Na_3Bi to NaBi and lastly to Bi. These results indicated the completely reversible transformations among Bi, NaBi, and Na_3Bi (Fig. 4F).

The volume variation of the incorporated Bi nanoparticles in HBiC during the initial sodiation-desodiation process was monitored by in situ TEM (Fig. 4G and movie S1). A progressive volume expansion occurred rapidly and, after 83 s (full sodiation), led to the expansion of a particle size of 51.7% compared with original Bi nanoparticles (Fig. 4H). This value was markedly smaller than that of the theoretical volume expansion of Bi during the sodiation-desodiation process (352%), which provided further evidence to the accommodation capability of the structured carbon matrix. In situ SAED showed that the Bi could progressively transform to NaBi with the insertion of Na^+ ions. Subsequent sodiation leaded to additional diffraction rings, suggesting the further alloying reaction between Bi metal and NaBi and the formation of Na_3Bi. The Bi nanoparticles exhibited gradual volume shrinkage during the subsequent desodiation process, coupled with the changes of diffraction rings. These results suggested that the HBiC kept its structural integrity without mechanical degradation and cracking during these processes (movie S2 and fig. S33).

A full Na^+ cell was assembled by coupling the HBiC anode with a commercial $Na_3V_2(PO_4)_3$ (NVP) cathode, denoted as HBiC//NVP, and measured in the 1.6- to 3.2-V voltage window (fig. S34). The HBiC//NVP existed a reversible capacity of 256 mA·hour g^{-1} after 100 cycles test at 1.0 A g^{-1} without any capacity decay. Moreover, the reversible capacities were maintained at 260, 258, 255, 253, 253, 246, 240, and 229 mA·hour g^{-1} at 0.1, 0.2, 0.5, 0.8, 1.0, 2.0, 3.0, and 5.0 A g^{-1}, respectively. With the increasing in current density, the reversible capacities and voltage plateaus were found to be slightly reduced due to small polarization in the full cells arisen from the fast kinetics (fig. S35). Moreover, HBiC//NVP could deliver an energy density of 76.6 watt-hour (Wh) kg^{-1} at 29.5 W kg^{-1}. Even at 1445.6 W kg^{-1}, the energy density was still 66.2 Wh kg^{-1}. Comparison on electrochemical parameters of specific energy/power densities between the assembled HBiC//NVP full cell and other prototype instances are shown in a Ragone plot (fig. S36). These results suggested that HBiC//NVP could be a potential electrode material having high power, high energy densities, and high output voltage.

Our work shows that the multiple inherent molecular interactions of the simple, natural building blocks EA and Bi^{3+} enable the self-assembly of hierarchical mesocrystals with quaternary structure in a spatiotemporally controlled manner. The relative strong π-π interaction among EA-Bi layers facilitates the formation of supramolecular threads that further assemble into nanoscale filaments driven by dipole-dipole force and, eventually, superstructure into hierarchical mesocrystals. The hierarchical structure of the mesocrystals was preserved after thermal transformation into a Bi-carbon hybrid, presenting outstanding sodium energy storage performance. This work probes the fundamentals of hierarchical self-assembly by exploring a new class of natural building blocks apart from nucleobases and peptides and provides new simple manufacturing pathways of complex micro-macroscale hybrid materials and devices with enhanced performance.

MATERIALS AND METHODS

General chemicals

EA ($C_{14}H_6O_8$; 97%,) was purchased from Acros (Belgium). $Bi(NO_3)_3$ pentahydrate [$Bi(NO_3)_3{\cdot}5H_2O$; 99%] and NMP (99%) were purchased from Chron Chemicals Co. Ltd. (China). Tannic acid (TA; $C_{76}H_{52}O_{46}$, AR) was purchased from Sigma-Aldrich. Commercial CNTs were purchased from Chengdu Organic Chemicals Co. Ltd., Chinese Academy of Sciences. Aluminum chloride hexahydrate ($AlCl_3{\cdot}6H_2O$; 98%), cupric chloride ($CuCl_2$; 98%), cobalt chloride ($CoCl_2$; 98%), iron(III) chloride ($FeCl_3$; 98%), commercial bismuth citrate (BC; $C_6H_5BiO_7$; 98%), NaCl (99%), urea (99%), and Tween 80 (99%) were purchased from Adams-beta (China). EtOH, EtOAc, toluene, and DMF solvents were purchased from General Reagents. All these materials were used as received. Milli-Q water with a resistivity of 18.2 megohm·cm was obtained from Merck Elix Advantage water purification system. All solutions were freshly prepared for immediate use in each experiment.

Synthesis of Bi-EA mesocrystals and HBiC

EA (0.26 mmol) and $Bi(NO_3)_3{\cdot}5H_2O$ (0.52 mmol) were dissolved in NMP (25 ml) under ambient condition without stirring. After aging for 7 days, a light yellow precipitate was obtained and collected by centrifugation (5000 rpm for 5 min). The precipitate was further washed with NMP (1 × 50 ml) and EtOH (2 × 50 ml). The Bi-EA mesocrystals were collected by centrifugation (5000 rpm for 5 min) and dried at 40°C under vacuum. The corresponding HBiC was obtained by annealing EA-Bi mesocrystals at 800°C for 2 hours under argon atmosphere with a heating rate of 5°C min^{-1}.

The preparation of materials with different cations and solvents were conducted in similar methods as that of Bi-EA mesocrystals. For different cations, $Bi(NO_3)_3{\cdot}5H_2O$ was replaced with $FeCl_3$, $AlCl_3{\cdot}6H_2O$, $CuCl_2$, and $CoCl_2$, respectively. For different solvent, NMP was replaced with DMF, EtOH, EtOAc, and toluene, respectively.

The preparation of HBiC-1 and HBiC-4 were described as follows: EA (0.26 mmol) and $Bi(NO_3)_3{\cdot}5H_2O$ (0.26 and 1.04 mmol) were dissolved in NMP (25 ml) under ambient condition without stirring, respectively. After aging for 7 days, the corresponding precipitates (EA-Bi–1 and EA-Bi–4) were further washed and collected as above-mentioned treatment. Last, the HBiC-1 and HBiC-4 were obtained after carbonized as following the above thermal treatment process, respectively.

Preparation of the Bi-C materials

In a typical preparation, 2.0 g of BC was annealed at 800°C for 2 hours in argon atmosphere with a heating rate of 2°C min^{-1}. After being cooled down to room temperature, the product of the Bi-C materials was collected.

Preparation of the Bi-CNTs composite

Fifty micrograms of CNTs were dispersed into 30 ml of distilled water, and the 1.0 ml of TA solution (40 mg ml^{-1}) was added. After

mixing, the 1.5 ml of Bi^{3+} in NMP solution was further added with well mixing. Then, the precipitates were collected, and after annealing at 800°C for 2 hours in argon atmosphere with a heating rate of 2°C min^{-1}, the Bi-CNT materials were prepared.

General characterization

Instruments and software

The crystal structure and phase analysis were identified by XRD (Cu Kα radiation, λ = 1.54178 Å; Philips X'PERT TROMPD). SAXS measurement was performed with Bruker NANOSTAR U SAXS (50 kV, 0.6 mA, λ = 1.54 Å). FT-Raman spectra were taken on a Raman spectrophotometer (HORIBA Jobin Yvon, HR800, France) with a 532-nm laser excitation in the range of 200 to 2000 cm^{-1}. SEM images were observed by a Hitachi S-4800 field-emission scanning electron microscope (Japan). TEM characterizations were performed using JEOL 2100F. XPS (ESCALAB 250Xi+, Thermo Fisher Scientific, USA) of the as-prepared composites were recorded using Mg Kα x-rays ($h\nu$ = 1253.6 eV) and a pass energy of 31.5 eV. AFM observation was carried out by Bruker MultiMode (Bruker, Dimension Icon, USA). N_2 adsorption-desorption isotherms and pore size distributions were characterized using a Kubo-X1000 analyzer (Beijing Builder Electronic Technology Co. Ltd.). The specific surface area was analyzed using the Brunauer-Emmett-Teller method. The pore size distribution was derived from the desorption branches of isotherms using the Barrett-Joyne-Halenda model. Positive ion mode MS was obtained using the Bruker solanX70 FT-MS. Sample (1 μl, 0.5 mg ml^{-1}) was injected into ESI ion source. Water/acetonitrile (v/v = 1:1) was used as effluent solvent. The thermogravimetric analysis was performed by Shimadzu DTG-60H (Shimadzu, Japan) with a heating rate of 10°C min^{-1} in air. The contents of metal ions were tested by ICP-OES. UV-Vis spectra were acquired on a PerkinElmer LAMBDA 1050 spectrophotometer. Fluorescence spectra were acquired on a Hitachi Fluorescence Spectrophotometer F-7000.

Electrochemical measurements

The working electrodes were prepared by a coated Cu foil with a slurry, containing the HBiC composite [80 weight % (wt %)], acetylene black (10 wt %; Tianjin Ebory Chemical Co. Ltd.), sodium carboxymethylcellulose binder (1000 to 1400 mPa·s, 10 wt %; Aladdin), and deionized water. After drying at 80°C for 12 hours, the coated Cu foil was punched into disks with a diameter of 12 mm, and the areal loading of active materials was approximately 0.9 to 1.2 mg cm^{-2} per slice. The NVP cathode was prepared by blending of NVP powder (Guangdong Card New Energy Technology Co. Ltd.), acetylene black, and polyvinylidene fluoride (Arkema, HSV 900) in a mass ratio of 9.0:0.5:0.5 in NMP and then coating on an Al foil. The corresponding area loading was about 6.0 mg cm^{-2}. For the Na^+ ion half cells, CR2016 coin-type cells were assembled in an argon-filled glove box. Sodium metal was used as the reference electrode and counter electrode, while Celgard 2325 (polypropylene/polyethylene/polypropylene) severed as the separator. $NaPF_6$ in 1,2-dimethixyethane (1.0 M) was used as the electrolyte. A battery test system (Neware, China) was used to test the electrochemical performances with a voltage window of 0.01 to 1.5 V (versus Na/Na^+) for HBiC anode and 2.0 to 3.8 V for NVP cathode. The HBiC anode and NVP cathode were paired up in a mass ratio of 1:6 in CR2032 coin-type cells, of which electrochemical performances were performed between 1.6 and 3.2 V. CV was carried out on a PARSTAT multichannel electrochemical workstation (Princeton Applied Research, PMC1000DC, USA).

In situ XRD measurement

The in situ XRD of the HBiC was carried out by a Rigaku Ultima IV power XRD with Cu Kα radiation at 40 kV and 40 mA. A homemade battery with an x-ray transparent Be window was connected with a LANNE CT2001 battery instrument with a current density of 100 mA g^{-1}. Data were collected in the 2θ range of 20° to 50° with a step width of 2.5° min^{-1}.

In situ TEM measurement

The dynamic sodiation-desodiation processes were conducted by in situ TEM using a nanobattery system (Nanofactory TEM holder operated at 200 kV). HBiC was attached to a Cu tip, and a piece of Na metal was mounted on a W tip. The native Na_2O on the Na metal surface served as a solid electrolyte. Once the contact between HBiC and Na_2O was established, a constant bias of −2.0/2.0 V was applied to the HBiC and Na metal to drive sodiation and desodiation reactions.

SAXS data fitting

The SAXS data were analyzed by McSAS package 1.3.1, with a minimal assumption Monte Carlo method. A sphere model was used to fit the collected data. The q range was 0.0711 to 2.28 nm^{-1}, corresponding to the sphere size in the range of 1.38 to 44.2 nm; the number of smearing points around each q was 25; the minimum uncertainty estimate was 1%; the target number of bins was 100; the variance of Gaussian beam profile was 0.0115 nm^{-1}; the convergence criterion was 1; the number of repetitions was 10; and the fitted data that showed the section diameters of the filament were from 1.38 to 44.2 nm with the number of bins of 50.

DFT calculations

The quantum chemical calculations in this work were performed using Gaussian 09 program package (*42*). Full geometry optimizations in NMP solvent were performed to locate all the stationary points, using the B3LYP (*43*) method with the 6-311 + $G(d, p)$ (*44*) basis set for C, H, O, and S; atoms; and the lanl2dz (*45*) basis set for Bi atoms, namely, B3LYP/6-311+ $G(d, p)$, lanl2dz at 298.25 K. Dispersion corrections were computed with Grimme's D3(BJ) method in optimization (*46*). The self-consistent reaction field method based on the universal solvation model SMD was adopted to evaluate the effect of the solvent (*47*). Basis set superposition error (BSSE) was taken into consideration of the calculation of the interaction energy (E_i), which was obtained by Eq. 1

$$E_i = E_{(a+b)} - E_a - E_b + E_{BSSE} \quad (1)$$

Where $E_{(a+b)}$ represented the total energy after the interaction of fragment a and b, E_a and E_b represented the energies of a and b species, respectively, and E_{BSSE} represented the BSSE energy. Unless specified, the Gibbs free energies at 298.25 K were used in Results and Discussion.

In addition, the first-principles calculation was used to describe the properties of Bi surface structure based on DFT. All calculation was carried out with pseudo-potential by the projector augmented wave and the Perdew-Burke-Ernzerhof. In addition, the cutoff energy was set to 400 eV, and the Brillouin zone was used with a 4 × 4 × 4 Γ-centered k-mesh for 2 × 2 × 2 supercell and 3 × 3 × 1 Γ-centered k-mesh for surface structure. All structures were optimized until the self-consistent force was less than 0.03 eV $Å^{-1}$ and the energy between two consecutive steps was less than 10^{-6} eV. Last, Na^+ migration barrier energy was calculated using the climbing nudged elastic band.

The adsorption energy was calculated by Eq. 2

$$E_d = E_{total} - EV_{total} - E_1 \quad (2)$$

where the E_{total} was the Bi surface structure with atom adsorbed, EV_{total} was the energy of the Bi surface structure, and E_1 was the energy of the atom.

The surface energy was calculated by Eq. 3

$$E_s = (EV_{total} - nE_{bulk})/2A \quad (3)$$

where the E_{bulk} was the energy of the Bi structure and A was the area of the surface.

Crystal unit modeling of tetracoordinate and pentacoordinate complexes was built with Material Studio v8.0. A reported unit cell data (*30*) of EA were used for modeling (a = 7.6561 Å, b = 9.5631 Å, c = 4.2631 Å, α = 97.881°, β = 103.21°, γ = 102.22°, V = 315.9 Å^3, d = 1.78 g ml^{-3}, and P − 1, Z = 1). Simulated XRD patterns were calculated with powder diffraction reflex module.

SUPPLEMENTARY MATERIALS

Supplementary material for this article is available at https://science.org/doi/10.1126/sciadv.abh3482

REFERENCES AND NOTES

1. C. Yuan, W. Ji, R. Xing, J. Li, E. Gazit, X. Yan, Hierarchically oriented organization in supramolecular peptide crystals. *Nat. Rev. Chem.* **3**, 567–588 (2019).
2. M. R. Jones, N. C. Seeman, C. A. Mirkin, Programmable materials and the nature of the DNA bond. *Science* **347**, 1260901 (2015).
3. Y. Lu, J. Lin, L. Wang, L. Zhang, C. Cai, Self-assembly of copolymer micelles: Higher-level assembly for constructing hierarchical structure. *Chem. Rev.* **120**, 4111–4140 (2020).
4. Y. Sun, C. Chen, P. J. Stang, Soft materials with diverse suprastructures via the self-assembly of metal-organic complexes. *Acc. Chem. Res.* **52**, 802–817 (2019).
5. T. Christoff-Tempesta, Y. Cho, D. Y. Kim, M. Geri, G. Lamour, A. J. Lew, X. Zuo, W. R. Lindemann, J. H. Ortony, Self-assembly of aramid amphiphiles into ultra-stable nanoribbons and aligned nanoribbon threads. *Nat. Nanotechnol.* **16**, 447–454 (2021).
6. D. J. Park, C. Zhang, J. C. Ku, Y. Zhou, G. C. Schatz, C. A. Mirkin, Plasmonic photonic crystals realized through DNA-programmable assembly. *Proc. Natl. Acad. Sci. U.S.A.* **112**, 977–981 (2015).
7. Y. F. Chao, R. Jalili, Y. Ge, C. Y. Wang, T. Zheng, K. W. Shu, G. G. Wallace, Self-assembly of flexible free-standing 3D porous MoS_2-reduced graphene oxide structure for high-performance lithium-ion batteries. *Adv. Funct. Mater.* **27**, 1700234 (2017).
8. Z. Fan, L. Sun, Y. Huang, Y. Wang, M. Zhang, Bioinspired fluorescent dipeptide nanoparticles for targeted cancer cell imaging and real-time monitoring of drug release. *Nat. Nanotechnol.* **11**, 388–394 (2016).
9. K. J. Chen, D. G. Madden, S. Mukherjee, T. Pham, K. A. Forrest, A. Kumar, B. Space, J. Kong, Q. Y. Zhang, M. J. Zaworotko, Synergistic sorbent separation for one-step ethylene purification from a four-component mixture. *Science* **366**, 241–246 (2019).
10. M. Mon, R. Bruno, S. Sanz-Navarro, C. Negro, J. Ferrando-Soria, L. Bartella, L. Di Donna, M. Prejano, T. Marino, A. Leyva-Perez, D. Armentano, E. Pardo, Hydrolase-like catalysis and structural resolution of natural products by a metal-organic framework. *Nat. Commun.* **11**, 3080 (2020).
11. R. Freeman, M. Han, Z. Alvarez, J. A. Lewis, J. R. Wester, N. Stephanopoulos, M. T. Mcclendon, C. Lynsky, J. M. Godbe, H. Sangji, E. Luijten, S. I. Stupp, Reversible self-assembly of superstructured networks. *Science* **362**, 808–813 (2018).
12. Y. Bai, Q. Luo, J. Liu, Protein self-assembly via supramolecular strategies. *Chem. Soc. Rev.* **45**, 2756–2767 (2016).
13. A. F. Mason, B. C. Buddingh, D. S. Williams, J. C. M. Van Hest, Hierarchical self-assembly of a copolymer-stabilized coacervate protocell. *J. Am. Chem. Soc.* **139**, 17309–17312 (2017).
14. X. Liu, F. Zhang, X. Jing, M. Pan, P. Liu, W. Li, B. Zhu, J. Li, H. Chen, L. Wang, J. Lin, Y. Liu, D. Zhao, H. Yan, C. Fan, Complex silica composite nanomaterials templated with DNA origami. *Nature* **559**, 593–598 (2018).
15. O. Vonshak, Y. Divon, S. Forste, D. Garenne, V. Noireaux, R. Lipowsky, S. Rudorf, S. S. Daube, R. H. Bar-Ziv, Programming multi-protein assembly by gene-brush patterns and two-dimensional compartment geometry. *Nat. Nanotechnol.* **15**, 783–791 (2020).
16. R. Chakrabarty, P. S. Mukherjee, P. J. Stang, Supramolecular coordination: Self-assembly of finite two- and three-dimensional ensembles. *Chem. Rev.* **111**, 6810–6918 (2011).
17. D. Kashiwagi, H. K. Shen, S. Sim, K. Sano, Y. Ishida, A. Kimura, T. Niwa, H. Taguchi, T. Aida, Molecularly engineered "janus groel": Application to supramolecular copolymerization with a higher level of sequence control. *J. Am. Chem. Soc.* **142**, 13310–13315 (2020).
18. C. Li, Q. Li, Y. V. Kaneti, D. Hou, Y. Yamauchi, Y. Mai, Self-assembly of block copolymers towards mesoporous materials for energy storage and conversion systems. *Chem. Soc. Rev.* **49**, 4681–4736 (2020).
19. H. Qiu, Z. M. Hudson, M. A. Winnik, I. Manners, Multidimensional hierarchical self-assembly of amphiphilic cylindrical block comicelles. *Science* **347**, 1329–1332 (2015).
20. H. Ejima, J. J. Richardson, K. Liang, J. P. Best, M. P. Van Koeverden, G. K. Such, J. Cui, F. Caruso, One-step assembly of coordination complexes for versatile film and particle engineering. *Science* **341**, 154–157 (2013).
21. J. Guo, B. L. Tardy, A. J. Christofferson, Y. Dai, J. J. Richardson, W. Zhu, M. Hu, Y. Ju, J. Cui, R. R. Dagastine, I. Yarovsky, F. Caruso, Modular assembly of superstructures from polyphenol-functionalized building blocks. *Nat. Nanotechnol.* **11**, 1105–1111 (2016).
22. J. Guo, M. Suastegui, K. K. Sakimoto, V. M. Moody, G. Xiao, D. G. Nocera, N. S. Joshi, Light-driven fine chemical production in yeast biohybrids. *Science* **362**, 813–816 (2018).
23. H. Lee, S. M. Dellatore, W. M. Miller, P. B. Messersmith, Mussel-inspired surface chemistry for multifunctional coatings. *Science* **318**, 426–430 (2007).
24. S. J. Yang, M. Antonietti, N. Fechler, Self-assembly of metal phenolic mesocrystals and morphosynthetic transformation toward hierarchically porous carbons. *J. Am. Chem. Soc.* **137**, 8269–8273 (2015).
25. Z. Bao, J. Wang, Z. Zhang, H. Xing, Q. Yang, Y. Yang, H. Wu, R. Krishna, W. Zhou, B. Chen, Q. Ren, Molecular sieving of ethane from ethylene through the molecular cross-section size differentiation in gallate-based metal-organic frameworks. *Angew. Chem. Int. Ed.* **57**, 16020–16025 (2018).
26. Z. Lin, J. Zhou, C. Cortez-Jugo, Y. Han, Y. Ma, S. Pan, E. Hanssen, J. J. Richardson, F. Caruso, Ordered mesoporous metal-phenolic network particles. *J. Am. Chem. Soc.* **142**, 335–341 (2020).
27. P. Xiong, P. Bai, A. Li, B. Li, M. Cheng, Y. Chen, S. Huang, Q. Iang, X.-H. Bu, Y. Xu, Bismuth nanoparticle@carbon composite anodes for ultralong cycle life and high-rate sodium-ion batteries. *Adv. Mater.* **31**, 1904771 (2019).
28. W. Xu, H. Wang, Y. Tao, X. Zheng, The structural organization of *N*-methyl-2-pyrrolidinone in binary mixtures probed by Raman spectroscopy: Experimental and quantum chemical results. *J. Raman Spectrosc.* **49**, 362–371 (2018).
29. S. J. Lee, B. S. Cheong, H. G. Cho, Surface-enhanced Raman spectroscopic studies of ellagic acid in silver colloids. *Bull. Kor. Chem. Soc.* **36**, 1637–1644 (2015).
30. M. Rossi, J. Erlebacher, D. E. Zacharias, H. L. Carrell, B. Iannucci, The crystal and molecular structure of ellagic acid dihydrate: A dietary anti-cancer agent. *Carcinogenesis* **12**, 2227–2232 (1991).
31. Z. Liu, W. He, Z. Guo, Metal coordination in photoluminescent sensing. *Chem. Soc. Rev.* **42**, 1568–1600 (2013).
32. C. F. Wang, R. F. Li, X. Y. Chen, R. J. Wei, L. S. Zheng, J. Tao, Synergetic spin crossover and fluorescence in one-dimensional hybrid complexes. *Angew. Chem. Int. Ed.* **54**, 1574–1577 (2015).
33. H. Yang, R. Xu, Y. Yao, S. Ye, X. Zhou, Y. Yu, Multicore-shell Bi@N-doped carbon nanospheres for high power density and long cycle life sodium- and potassium-ion anodes. *Adv. Funct. Mater.* **29**, 1809195 (2019).
34. P. Xue, N. Wang, Z. Fang, Z. Lu, X. Xu, L. Wang, Y. Du, X. Ren, Z. Bai, S. Dou, G. Yu, Rayleigh-instability-induced bismuth nanorod@nitrogen-doped carbon nanotubes as a long cycling and high rate anode for sodium-ion batteries. *Nano Lett.* **19**, 1998–2004 (2019).
35. C. Wang, L. Wang, F. Li, F. Cheng, J. Chen, Bulk bismuth as a high-capacity and ultralong cycle-life anode for sodium-ion batteries by coupling with glyme-based electrolytes. *Adv. Mater.* **29**, 1702212 (2017).
36. J. Chen, X. Fan, X. Ji, T. Gao, S. Hou, X. Zhou, L. N. Wang, F. Wang, C. Yang, L. Chen, C. Wang, Intercalation of Bi nanoparticles into graphite results in an ultra-fast and ultra-stable anode material for sodium-ion batteries. *Energy Environ. Sci.* **11**, 1218–1225 (2018).
37. H. Ying, W.-Q. Han, Metallic Sn-based anode materials: Application in high-performance lithium-ion and sodium-ion batteries. *Adv. Sci.* **4**, 1700298 (2017).
38. W. Li, S. Hu, X. Luo, Z. Li, X. Sun, M. Li, F. Liu, Y. Yu, Confined amorphous red phosphorus in MOF-derived N-doped microporous carbon as a superior anode for sodium-ion battery. *Adv. Mater.* **29**, 1605820 (2017).
39. W. Luo, F. Li, J.-J. Gaumet, P. Magri, S. Diliberto, L. Zhou, L. Mai, Bottom-up confined synthesis of nanorod-in-nanotube structured Sb@N-C for durable lithium and sodium storage. *Adv. Energy Mater.* **8**, 1703237 (2018).
40. J. Zhou, L. Wang, M. Yang, J. Wu, F. Chen, W. Huang, N. Han, H. Ye, F. Zhao, Y. Li, Y. Li, Hierarchical VS_2 nanosheet assemblies: A universal host material for the reversible storage of alkali metal ions. *Adv. Mater.* **29**, 1702061 (2017).
41. N. Nitta, G. Yushin, High-capacity anode materials for lithium-ion batteries: Choice of elements and structures for active particles. *Part. Part. Syst. Charact.* **31**, 317–336 (2014).

42. M. J. Frisch, G. W. Trucks, H. B. Schlegel, G. E. Scuseria, M. A. Robb, J. R. Cheeseman, G. Scalmani, V. Barone, B. Mennucci, G. A. Petersson, H. Nakatsuji, M. Caricato, X. Li, H. P. Hratchian, A. F. Izmaylov, J. Bloino, G. Zheng, J. L. Sonnenberg, M. Hada, M. Ehara, K. Toyota, R. Fukuda, J. Hasegawa, M. Ishida, T. Nakajima, Y. Honda, O. Kitao, H. Nakai, T. Vreven, J. A. Montgomery, J. J. E. Peralta, F. Ogliaro, M. Bearpark, J. J. Heyd, E. Brothers, K. N. Kudin, V. N. Taroverov, T. Keith, R. Kobayashi, J. Normand, K. Raghavachari, A. Rendell, J. C. Burant, S. S. Iyengar, J. Tomasi, M. Cossi, N. Rega, J. M. Millam, M. Klene, J. E. Knox, J. B. Cross, V. Bakken, C. Adamo, J. Jaramillo, R. Gomperts, R. E. Stratmann, O. Yazyev, A. J. Austin, R. Cammi, C. Pomelli, J. W. Ochterski, R. L. Martin, K. Morokuma, V. G. Zakrzewski, G. A. Voth, P. Salvador, J. J. Dannenberg, S. Dapprich, A. D. Daniels, O. Farkas, J. B. Foresman, J. V. Ortiz, J. Cioslowski, D. J. Fox, Gaussian 09 (revision d.01) v. Revision D.01, (Gaussian Inc., 2013).
43. Y. Zhao, D. G. Truhlar, Density functionals with broad applicability in chemistry. *Acc. Chem. Res.* **41**, 157–167 (2008).
44. R. Krishnan, J. S. Binkley, R. Seeger, J. A. Pople, Self-consistent molecular-orbital methods. 20. Basis set for correlated wave-functions. *J. Chem. Phys.* **72**, 650–654 (1980).
45. P. J. Hay, W. R. Wadt, *Ab* initio effective core potentials for molecular calculations. Potentials for the transition metal atoms Sc to Hg. *J. Chem. Phys.* **82**, 270–283 (1985).
46. S. Grimme, J. Antony, S. Ehrlich, H. Krieg, A consistent and accurate *ab initio* parametrization of density functional dispersion correction (DFT-D) for the 94 elements H-Pu. *J. Chem. Phys.* **132**, 154104 (2010).
47. A. V. Marenich, C. J. Cramer, D. G. Truhlar, Universal solvation model based on solute electron density and on a continuum model of the solvent defined by the bulk dielectric constant and atomic surface tensions. *J. Phys. Chem. B* **113**, 6378–6396 (2009).
48. Y. Wang, R. Xiao, Y. S. Hu, M. Avdeev, L. Chen, P2-$Na_{0.6}[Cr_{0.6}Ti_{0.4}]O_2$ cation-disordered electrode for high-rate symmetric rechargeable sodium-ion batteries. *Nat. Commun.* **6**, 6954 (2015).
49. S. Guo, H. Yu, P. Liu, Y. Ren, T. Zhang, M. Chen, M. Ishida, H. Zhou, High-performance symmetric sodium-ion batteries using a new, bipolar O3-type material, $Na_{0.8}Ni_{0.4}Ti_{0.6}O_2$. *Energy Environ. Sci.* **8**, 1237–1244 (2015).
50. Y. Wang, X. Yu, S. Xu, J. Bai, R. Xiao, Y.-S. Hu, H. Li, X.-Q. Yang, L. Chen, X. Huang, A zero-strain layered metal oxide as the negative electrode for long-life sodium-ion batteries. *Nat. Commun.* **4**, 2365 (2013).
51. J. Pan, S. Chen, D. Zhang, X. Xu, Y. Sun, F. Tian, P. Gao, J. Yang, SnP_2O_7 covered carbon nanosheets as a long-life and high-rate anode material for sodium-ion batteries. *Adv. Funct. Mater.* **28**, 1804672 (2018).
52. Y. Fang, L. Xiao, J. Qian, Y. Cao, X. Ai, Y. Huang, H. Yang, 3D graphene decorated $NaTi_2(PO_4)_3$ microspheres as a superior high-rate and ultracycle-stable anode material for sodium ion batteries. *Adv. Energy Mater.* **6**, 1502197 (2016).
53. M. Huang, B. Xi, Z. Feng, J. Liu, J. Feng, Y. Qian, S. Xiong, Facile synthesis of N,O-codoped hard carbon on the kilogram scale for fast capacitive sodium storage. *J. Mater. Chem. A* **6**, 16465–16474 (2018).
54. I. Hasa, X. Dou, D. Buchholz, Y. Shao-Horn, J. Hassoun, S. Passerini, B. Scrosati, A sodium-ion battery exploiting layered oxide cathode, graphite anode and glyme-based electrolyte. *J. Power Sources* **310**, 26–31 (2016).
55. Z. Li, D. Young, K. Xiang, W. C. Carter, Y.-M. Chiang, Towards high power high energy aqueous sodium-ion batteries: The $NaTi_2(PO_4)_3/Na_{0.44}MnO_2$ System. *Adv. Energy Mater.* **3**, 290–294 (2013).
56. Z. Hou, X. Li, J. Liang, Y. Zhu, Y. Qian, An aqueous rechargeable sodium ion battery based on a $NaMnO_2$–$NaTi_2(PO_4)_3$ hybrid system for stationary energy storage. *J. Mater. Chem. A* **3**, 1400–1404 (2015).
57. D. Su, S. Dou, G. Wang, Bismuth: A new anode for the Na-ion battery. *Nano Energy* **12**, 88–95 (2015).
58. S. Liu, J. Feng, X. Bian, J. Liu, H. Xu, Advanced arrayed bismuth nanorod bundle anode for sodium-ion batteries. *J. Mater. Chem. A* **4**, 10098–10104 (2016).
59. F. Yang, F. Yu, Z. Zhang, K. Zhang, Y. Lai, J. Li, Bismuth nanoparticles embedded in carbon spheres as anode materials for sodium/lithium-ion batteries. *Chem. Eur. J.* **22**, 2333–2338 (2016).
60. Y. Jin, H. Yuan, J.-L. Lan, Y. Yu, Y.-H. Lin, X. Yang, Bio-inspired spider-web-like membranes with a hierarchical structure for high performance lithium/sodium ion battery electrodes: The case of 3D freestanding and binder-free bismuth/CNF anodes. *Nanoscale* **9**, 13298–13304 (2017).
61. S. Liu, Z. Luo, J. Guo, A. Pan, Z. Cai, S. Liang, Bismuth nanosheets grown on carbon fiber cloth as advanced binder-free anode for sodium-ion batteries. *Electrochem. Commun.* **81**, 10–13 (2017).
62. J. Qiu, S. Li, X. Su, Y. Wang, L. Xu, S. Yuan, H. Li, S. Zhang, Bismuth nano-spheres encapsulated in porous carbon network for robust and fast sodium storage. *Chem. Eng. J.* **320**, 300–307 (2017).
63. L. Wang, C. Wang, F. Li, F. Cheng, J. Chen, In situ synthesis of Bi nanoflakes on Ni foam for sodium-ion batteries. *Chem. Commun.* **54**, 38–41 (2017).
64. H. Yin, Q. Li, M. Cao, W. Zhang, H. Zhao, C. Li, K. Huo, M. Zhu, Nanosized-bismuth-embedded 1D carbon nanofibers as high-performance anodes for lithium-ion and sodium-ion batteries. *Nano Res.* **10**, 2156–2167 (2017).
65. X. L. Cheng, D. J. Li, Y. Wu, R. Xu, Y. Yu, Bismuth nanospheres embedded in three-dimensional (3D) porous graphene frameworks as high performance anodes for sodium- and potassium-ion batteries. *J. Mater. Chem. A* **7**, 4913–4921 (2019).
66. H. Yang, L. W. Chen, F. He, J. Zhang, Y. Feng, L. Zhao, B. Wang, L. He, Q. Zhang, Y. Yu, Optimizing the void size of yolk-shell Bi@Void@C nanospheres for high-power-density sodium-ion batteries. *Nano Lett.* **20**, 758–767 (2020).
67. Y. Zhang, Q. Su, W. Xu, G. Cao, Y. Wang, A. Pan, S. Liang, A confined replacement synthesis of bismuth nanodots in MOF derived carbon arrays as binder-free anodes for sodium-ion batteries. *Adv. Sci.* **6**, 1900162 (2019).
68. J. Zhou, J. Chen, M. Chen, J. Wang, X. Liu, B. Wei, Z. Wang, J. Li, L. Gu, Q. Zhang, H. Wang, L. Guo, Few-layer bismuthene with anisotropic expansion for high-areal-capacity sodium-ion batteries. *Adv. Mater.* **31**, e1807874 (2019).

Acknowledgments: We thank H. Wang at the Analytical & Testing Center of Sichuan University for SEM characterization, X. He and Z. Wang at the College of Biomass Science and Engineering of Sichuan University for UV-Vis and fluorescence characterization, P. Li from Shiyanjia Lab (www.shiyanjia.com) for the TEM analysis, and Ceshigo for XPS characterization and DFT calculation. **Funding:** This work was supported by National Talents Program, Double First Class University Plan of Sichuan University, State Key Laboratory of Polymer Materials Engineering (grant no. sklpme2020-03-01), National Natural Science Foundation of China (21878192 and 51502180), the Fundamental Research Funds for the Central Universities (2016SCU04A18), Talents Program of Sichuan Province, Graduate Student's Research and Innovation Fund of Sichuan University (2018YJSY070), China Postdoctoral Science Foundation (2020TQ0209), and the Japan Society for the Promotion of Science Fellowship (P20373).

Author contributions: H.W., J.G., X.Q., and Y.Z. conceived the idea. X.Q., X.W., and Y.H. performed material synthesis, battery fabrication, and electrochemical measurements. X.W., J.L., and K.L. participated in part of the experiments. X.W., Y.H., and M.H. conducted the crystal modeling. X.Q., X.W., Y.H., J.J.R., B.L.T., and J.G. cowrote the paper. M.H., O.J.R., I.M., and J.G. contributed to the understanding and experimental design of self-assembly process. All authors discussed the results and commented on the manuscript. **Competing interests:** The authors declare that they have no competing interests. **Data and materials availability:** All data needed to evaluate the conclusions in the paper are present in the paper and/or the Supplementary Materials.

Submitted 3 March 2021
Accepted 20 July 2021
Published 8 September 2021
10.1126/sciadv.abh3482

Citation: X. Qiu, X. Wang, Y. He, J. Liang, K. Liang, B. L. Tardy, J. J. Richardson, M. Hu, H. Wu, Y. Zhang, O. J. Rojas, I. Manners, J. Guo, Superstructured mesocrystals through multiple inherent molecular interactions for highly reversible sodium ion batteries. *Sci. Adv.* **7**, eabh3482 (2021).
