# Supplementary material for: Superstructured mesocrystals through multiple inherent molecular interactions for highly reversible sodium ion batteries
Source: Sci Adv. 2021 Sep 8;7(37):eabh3482. doi: 10.1126/sciadv.abh3482 (PMC8442931; doi:10.1126/sciadv.abh3482)
Supplement: Supplementary file 2 — Figs. S1 to S36 Tables S1 to S6 Legends for movies S1 and S2 References [file sciadv.abh3482_sm.v2.pdf]

CORRECTED 15 APRIL 2022; SEE FULL TEXT

Science Advances
AAAS

advances.sciencemag.org/cgi/content/full/sciadv.[ms.no.]/DC1

Supplementary Materials for

Superstructured mesocrystals through multiple inherent molecular interactions for highly reversible sodium ion batteries

Xiaoling Qiu[†], Xiaoling Wang[†], Yunxiang He[†], Jieying Liang, Kang Liang, Blaise L. Tardy, Joseph J. Richardson, Ming Hu, Hao Wu[*], Yun Zhang[*], Orlando J. Rojas, Ian Manners, Junling Guo[*]

[†]These authors contributed equally to this work.
[*]Corresponding author. Email: hao.wu@scu.edu.cn (H.W.); y_zhang@scu.edu.cn (Y.Z.); junling.guo@scu.edu.cn, junlingguo@g.harvard.edu (J.G.)

The PDF file includes:

Figs. S1 to S36
Tables S1 to S6

Other Supplementary Material for this manuscript includes the following:

Movies S1 and S2

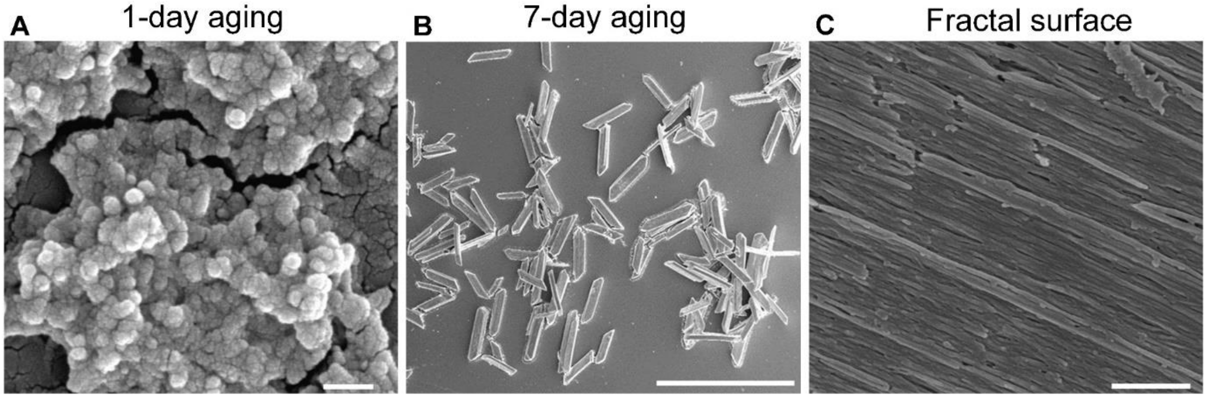

Fig. S1.
SEM characterization of Bi-EA mesocrystal formation. (A to C) SEM images of Bi-EA subunits after 1-day aging, mesocrystals after 7-day aging and fractal rough surface of mesocrystals. Scale bars are 200 nm (A), 200 μm (B) and 500 nm (C).

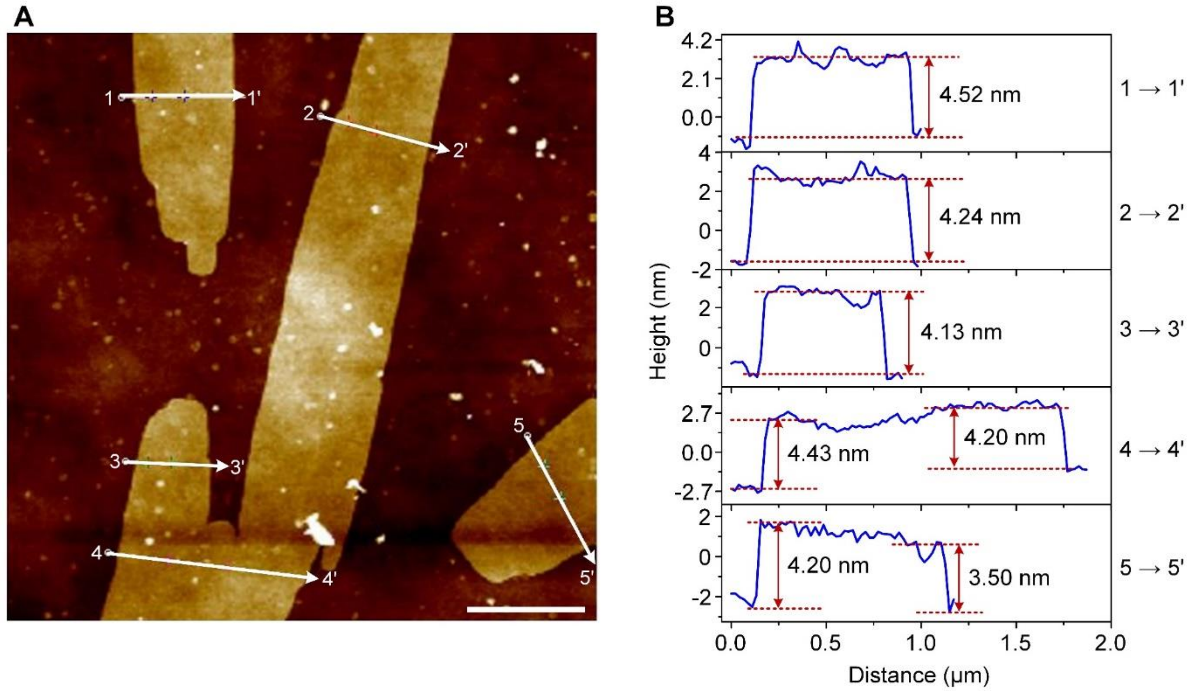

Fig. S2.
AFM image and corresponding height profiles of Bi-EA mesocrystals. Samples for AFM images were prepared by sonication in a water-bath at 23°C for 30 min with a mesocrystal suspension concentration of 0.5 mg mL^{-1}. The thin layers shown a height ~4.2 nm, which suggested the existence of subunits of filament structures. Scale bar is 1 μm (A).

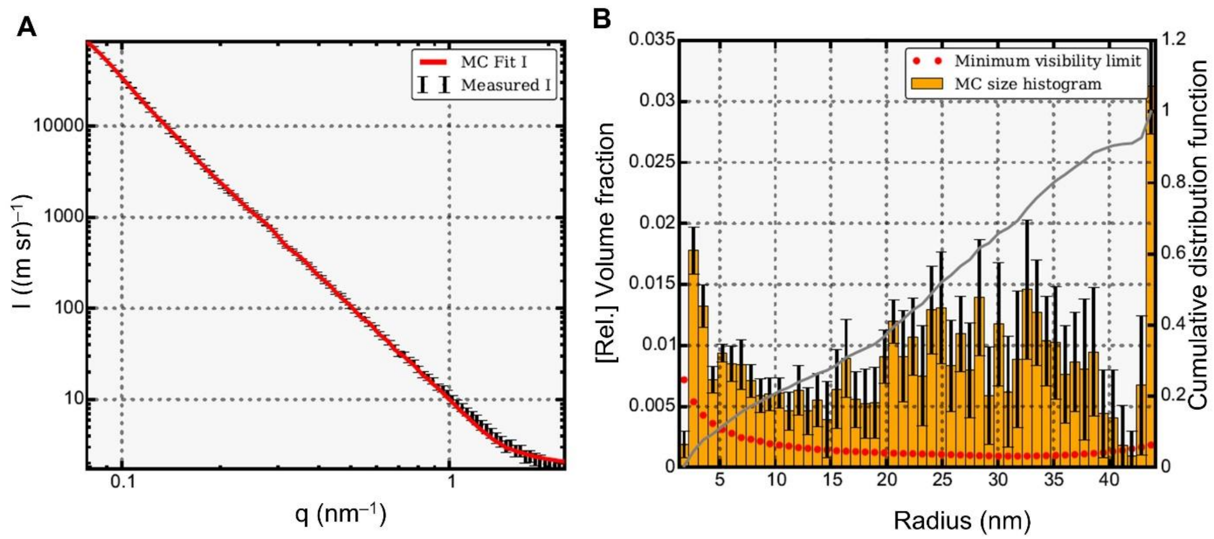

Fig. S3.

SAXS characterization of the Bi-EA mesocrystals and the model fitting for hierarchical structure analysis. (A) SAXS plot of the intensity (counts) vs. q ($Å^{-1}$) with measured data (black) and model fitted data (red). The collected data showed a power law slope and indicated the prepared sample was aggregated and polydisperse in size. (B) Distribution of the filament diameter by fitting with sphere model from SAXS data. The model fitting data suggested a distribution of the filament diameter from 1.8 − 44.2 nm. This suggested the polydispersity of the prepared sample, in which the subunits assembled randomly into filaments.

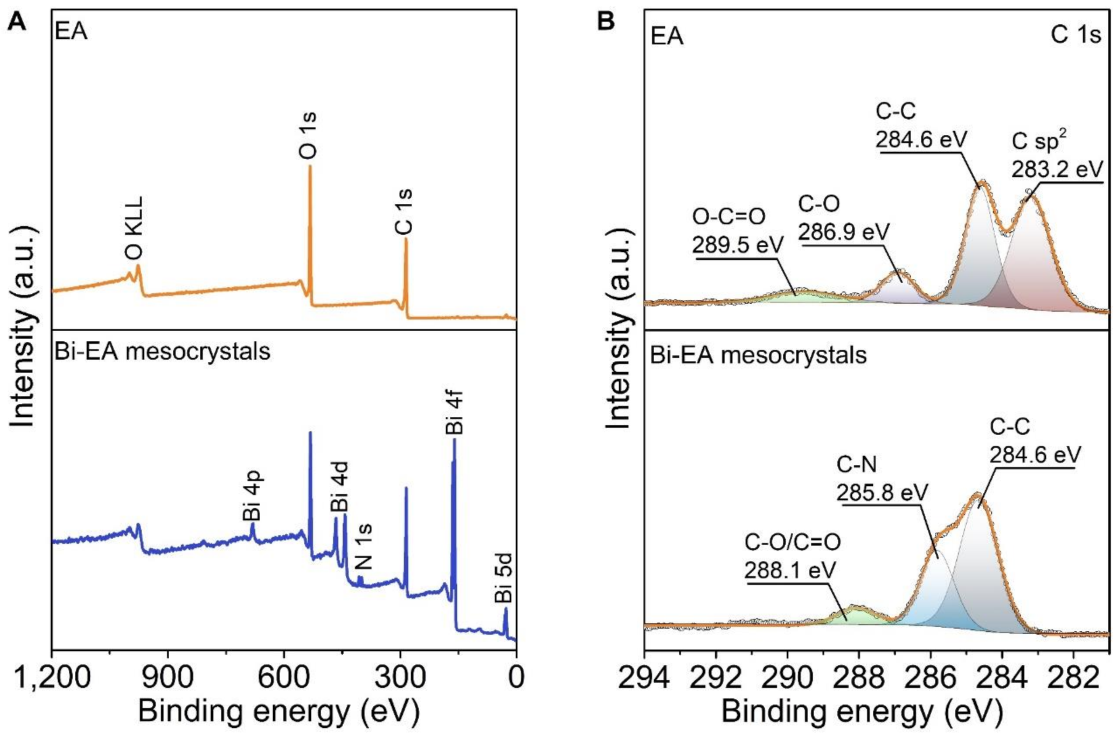

Fig. S4.

XPS analysis of EA and Bi-EA mesocrystals. (A) XPS survey spectra. (B) High-resolution XPS spectra of C 1s. A new C-N peak (285.8 eV) in C 1s spectra and the peak shifted and merged of C-O, C=O, and C-C bond, confirmed the coordination among EA, Bi^{3+} ions, and NMP molecules。

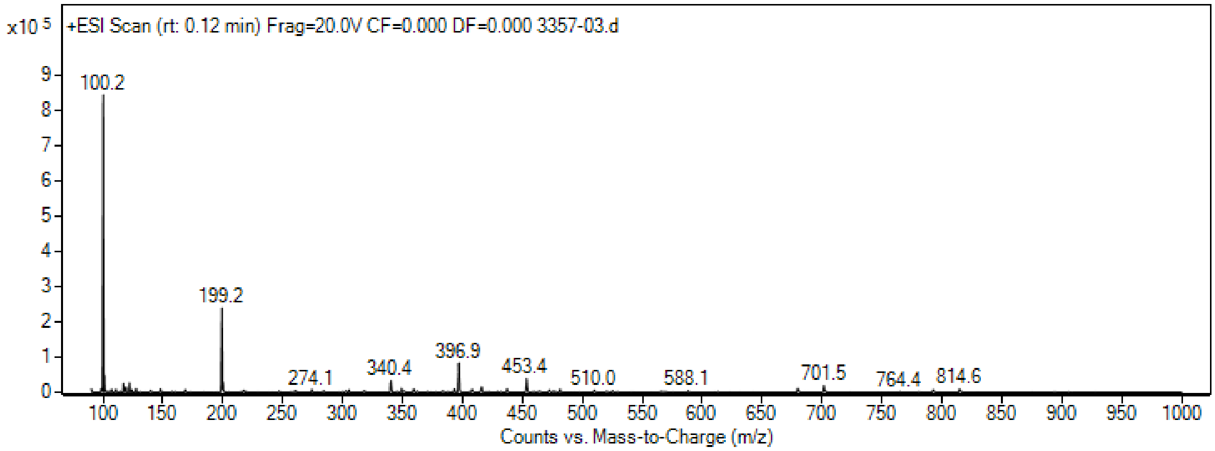

Fig. S5.

Mass spectrum of Bi-EA mesocrystals by ESI-MS. The fragment peak of 100.2 indicated the presence of NMP molecules in the mesocrystals.

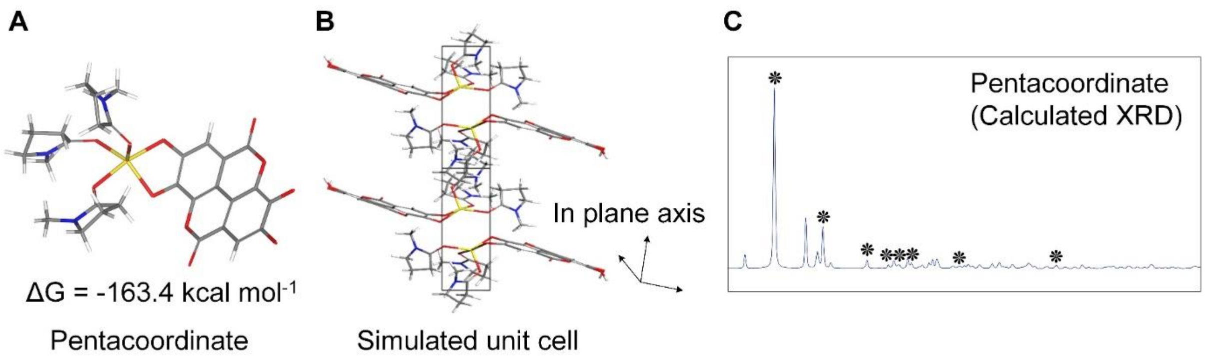

Fig. S6.
Simulation studies of the EA-Bi mesocrystals with penta-coordination primary structure. (A and B) Computational optimized pentacoordinate structure and corresponding simulated unit cell. (C) Calculated XRD pattern of pentacoordinate. The calculated XRD pattern of simulated pentacoordinate crystal unit cell presented identical peaks with the experimental ones, which suggested the presence of pentacoordinate structure characteristic, such as NMP solvent interactions in the mesocrystal.

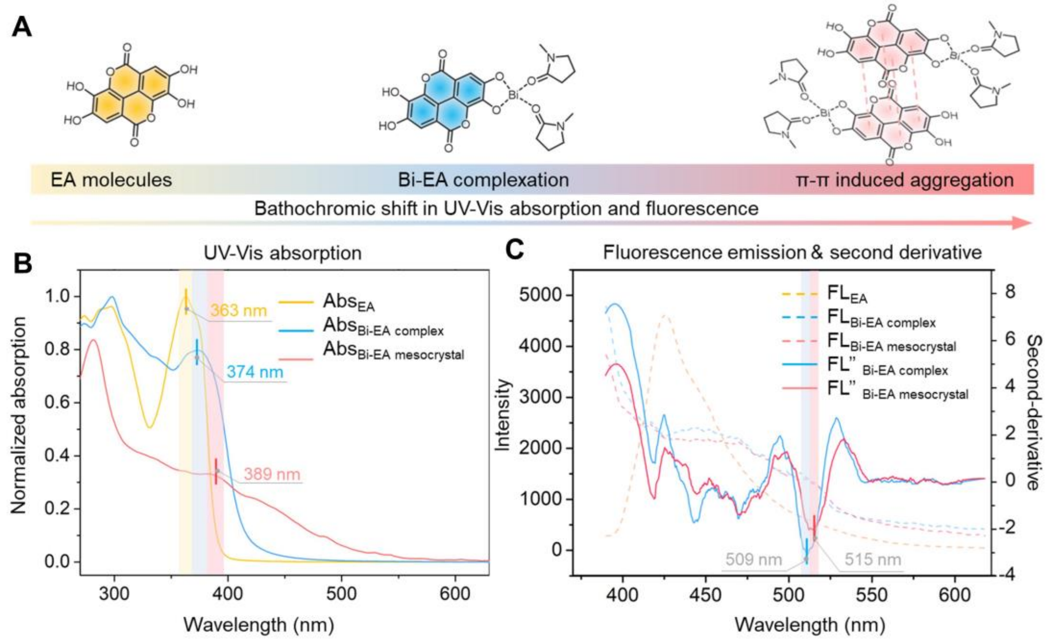

Fig. S7.

UV-vis absorption and fluorescence emission studies on the secondary structure of Bi-EA mesocrystals. (A) Structures of EA molecule, a Bi-EA complex, and π-π induced aggregation of Bi-EA complexes. (B) UV-Vis absorption spectra exhibited a bathochromic shift from 363 nm (EA molecule) to 374 nm (Bi-EA complex) due to complexation, followed by a further bathochromic shift to 389 nm (Bi-EA mesocrystal) due to π-π interactions. (C) Fluorescence (FL) spectra of EA, Bi-EA complex, and π-π induced aggregation of Bi-EA complexes, where a decreased fluorescent intensity of Bi-EA complex and Bi-EA mesocrystal compared with that of the EA molecule could be observed. Secondary-derivative spectra of Bi-EA complex and Bi-EA mesocrystal exhibited a bathochromic shift from 509 to 515 nm suggesting the formation of π-π induced aggregation.

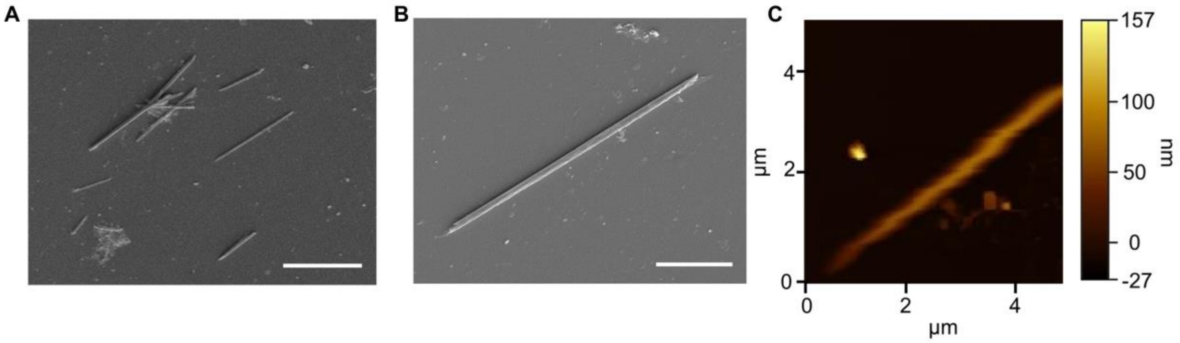

Fig. S8.
Morphological characterization of Bi-EA mesocrystals after NaCl treatment. (A and B) SEM and (C) AFM images of Bi-EA mesocrystals after NaCl treatment (200 mg mL^{-1}). Scale bars are 25 μm (A) and 10 μm (B).

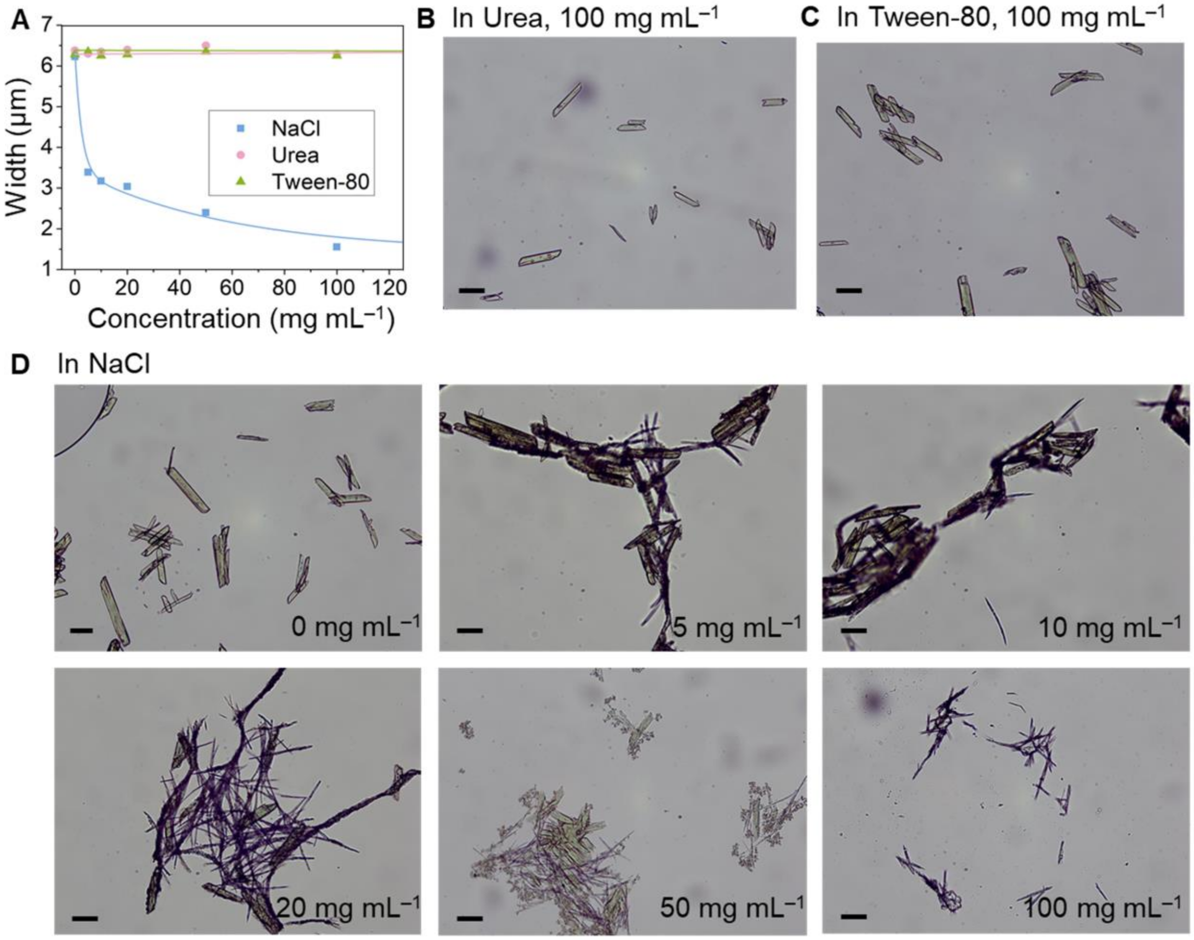

Fig. S9.

Disassembly of mesocrystals (5 mg mL^{-1} suspension in water) with additives in aqueous solution with the incubation at 37ºC for 1 h. (A) Plot of width (μm) vs. additive concentration (mg mL^{-1}). (B to D) Optical microscope images of mesocrystals in urea (100 mg mL^{-1}), Tween-80 (100 mg mL^{-1}) and NaCl (with varied concentration of 0 − 100 mg mL^{-1}). A disassembly process of mesocrystals into filaments in NaCl solution was observed with an increase of concentration. The results suggested that the assembly of filaments was driven by ionic interactions. Scale bars are 20 μm (B to D).

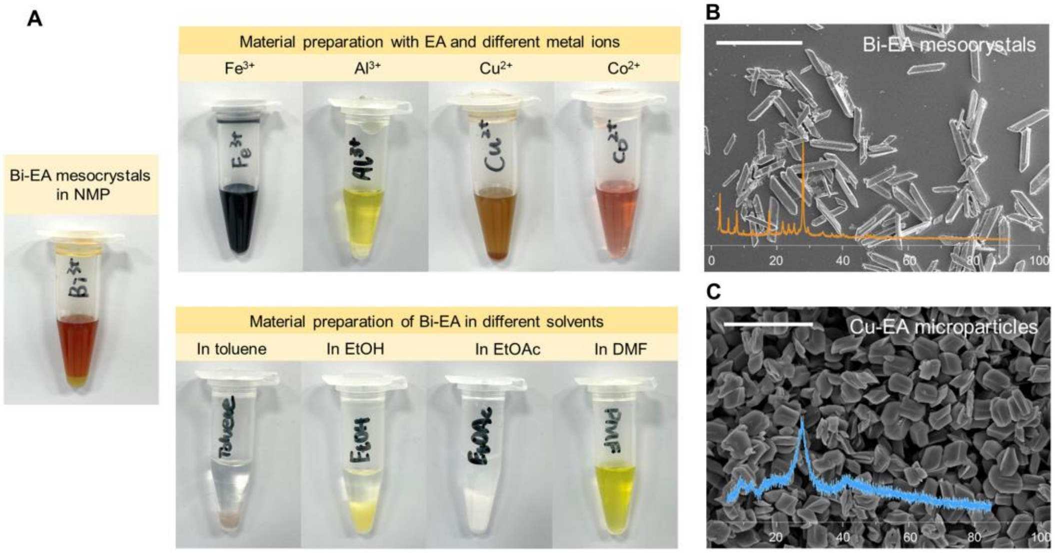

Fig. S10.

Metal ions and solvents screen for the mesocrystal preparation. (A) Photo images of samples prepared with different metal ions and solvents. For different cations, no precipitation or mesocrystal formation was seen with the usage of Fe^{3+} and Co^{2+} ions. In addition, Al^{3+} salts were not soluble in NMP, while the precipitation collected with Cu^{2+} ions had low crystallinity (C). For different solvents, either Bi^{3+} ions or EA were not soluble in toluene, EtOAc, and EtOH, while no precipitation was collected with DMF. (B and C) SEM images and XRD patterns of Bi-EA mesocrystals and Cu-EA microparticles. The morphology and crystallinity of the Cu-EA microparticles was different from that of the Bi-EA mesocrystals. Scale bars are 200 μm (B) and 5 μm (C). Photo credit (A): Xiaoling Qiu, Sichuan University.

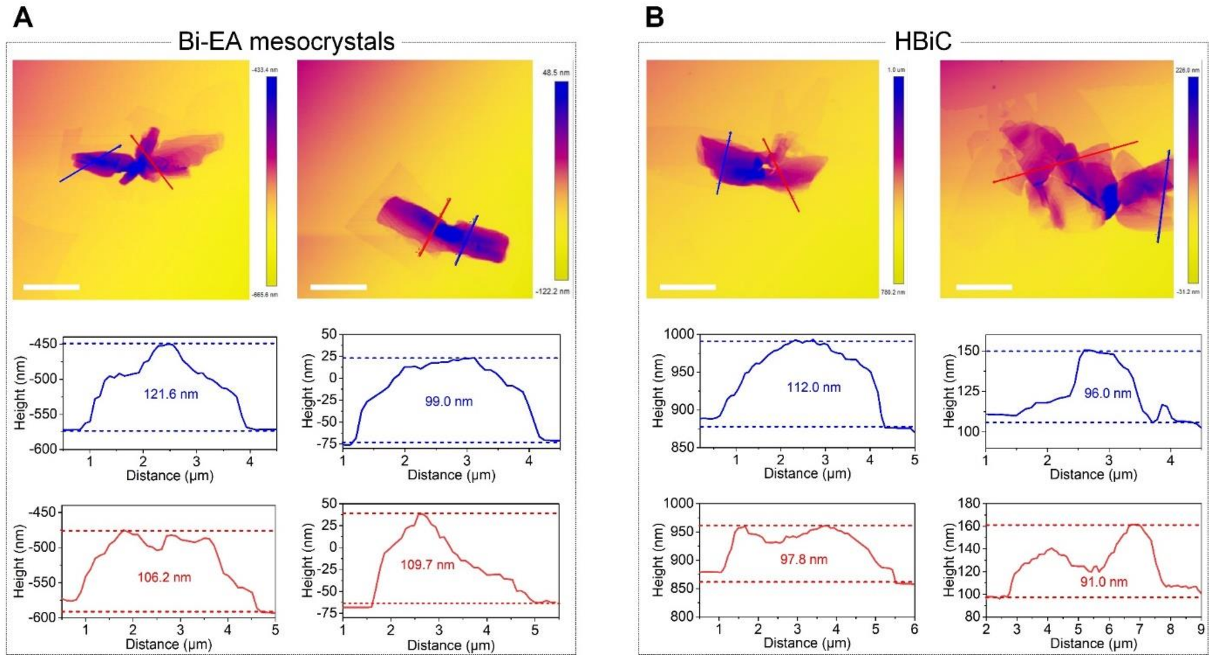

Fig. S11.
AFM images and corresponding height profiles of Bi-EA mesocrystals and HBiC hybrid. Scale bars are 5 μm (A and B).

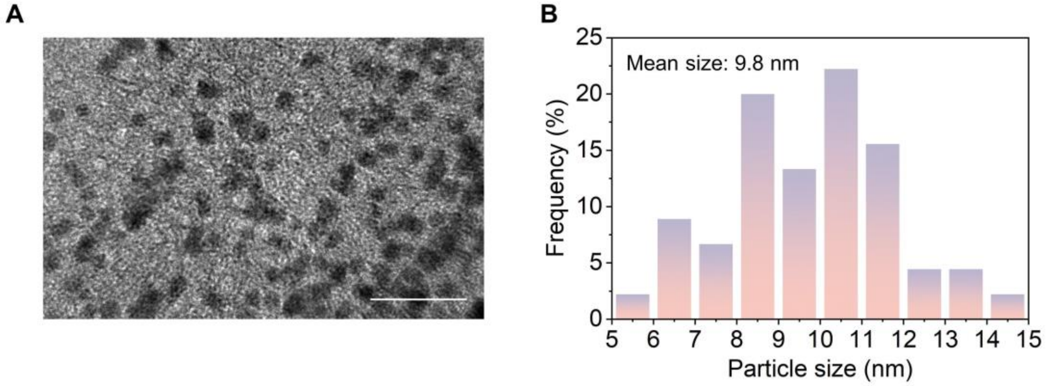

Fig. S12.
Particle size analysis of the Bi nanoparticles embedded in HBiC. (A) TEM image and (B) corresponding particle size distribution. Scale bar is 50 nm (A).

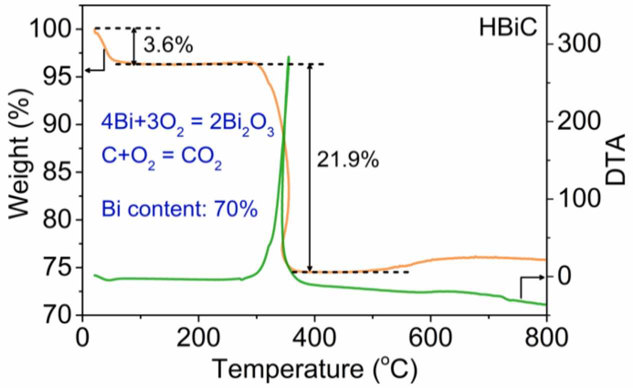

Fig. S13.
TGA curve (in air) of the HBiC. The Bi content was calculated based on the following equation and the weight content of Bi was 70%.

$$Bi\ (wt\%) = 100 \times \frac{2 \times molecular\ weight\ of\ Bi}{molecular\ weight\ of\ Bi_2O_3} \times \frac{final\ weight\ of\ Bi_2O_3}{initial\ weight\ of\ HBiC}$$

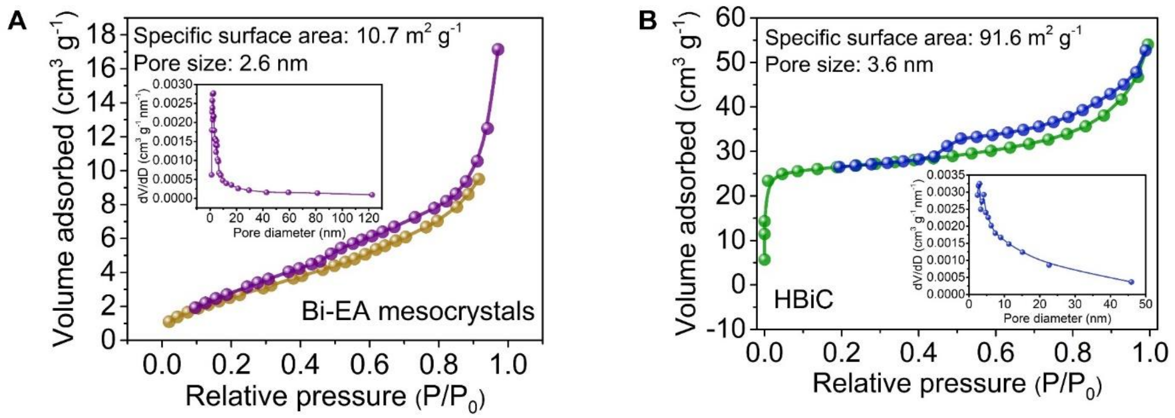

Fig. S14.

BET measurements of Bi-EA mesocrystals and HBiC. N_2 adsorption-desorption isotherms and pore size distributions (insets) of Bi-EA mesocrystals (A) and HBiC (B). As calculation from the adsorption isotherms using the BET theory and t-plots, the BET characteristic parameters determined by N_2 adsorption data of Bi-EA mesocrystals and HBiC were further showed in **table S3**. The BET specific surface area and total pore volume of HBiC were calculated to be 91.6 m^2 g^{-1} and 0.0836 cm^3 g^{-1}, respectively, which is larger than the value of Bi-EA mesocrystals (10.7 m^2 g^{-1} and 0.0322 cm^3 g^{-1}). The increased specific surface area and pore volume should be ascribed to the additional defects induced by the carbothermic reduction. Bi-EA mesocrystals showed pore size distribution range from 1.15 to 122.53 nm with an average pore of 2.6 nm, while the HBiC exhibited pores centered from 0.18 to 210.99 nm with an average pore size of 3.6 nm, indicating the micropores, mesopores, and macropores coexist in these two samples. Moreover, the Bi-EA mesocrystals showed no or negligible micropore (smaller than 2 nm diameter) volumes, while the HBiC showed high proportions of micropore specific surface area (77.4%) and micropore volume (37.9%) of the total N_2 adsorption isothermal. Accordingly, the electrolyte could freely get into the macropores possessing the sizes of > 50 nm, or even micron-size (2 − 50 nm), and infiltrated into the whole structure of HBiC easily. This hierarchical porous structure could increase the contact areas between the electrode and electrolyte and decrease the diffusion distance, which was in favor of the fast transportation of Na^+ ions.

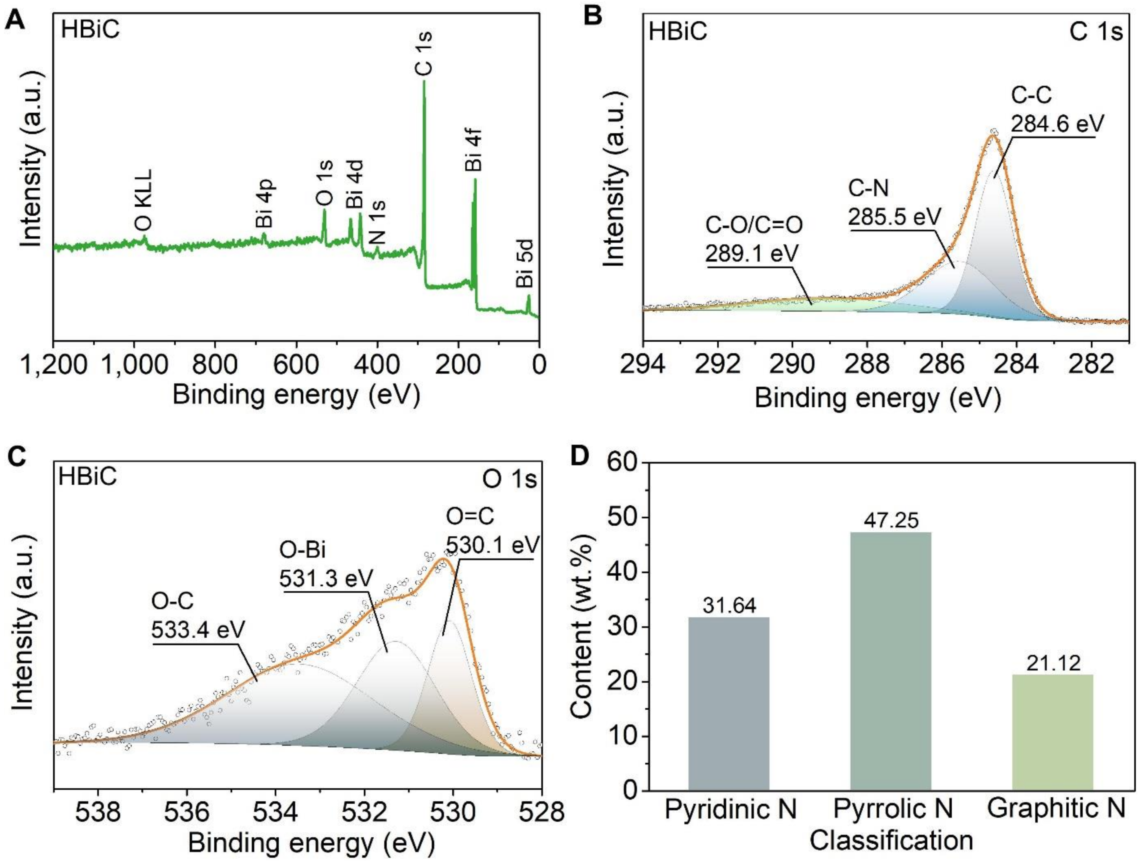

Fig. S15.
XPS analysis of HBiC. (A) XPS survey spectrum. (B and C) High-resolution spectra of C 1s and O 1s. The peaks for C-O/C=O bond (289.1 eV) in C 1s spectra and O-Bi bond (531.3 eV) in O 1s spectra suggested the existence of Bi-O-C bonds in the HBiC hybrid. (D) The relative contents of pyridinic N, pyrrolic N and graphitic N in N 1s spectra. The nitrogen content in the *N*-doped carbon was 5.36%, and this *N*-doped carbon matrix had been certified to enrich the Na^+ trapping defects, thus enhancing the sodium storage capabilities.

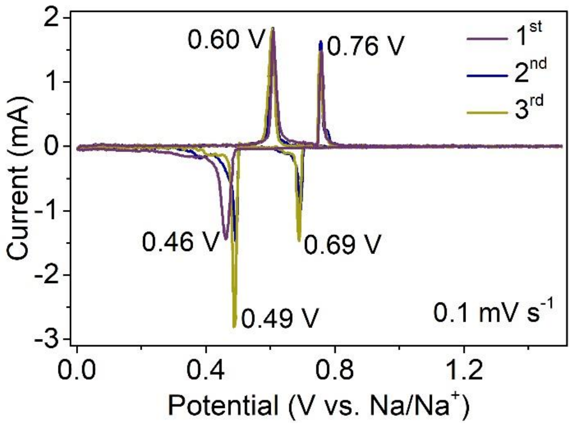

Fig. S16.

CV curves at a scan rate of 0.1 mV s^{-1} of HBiC anode.

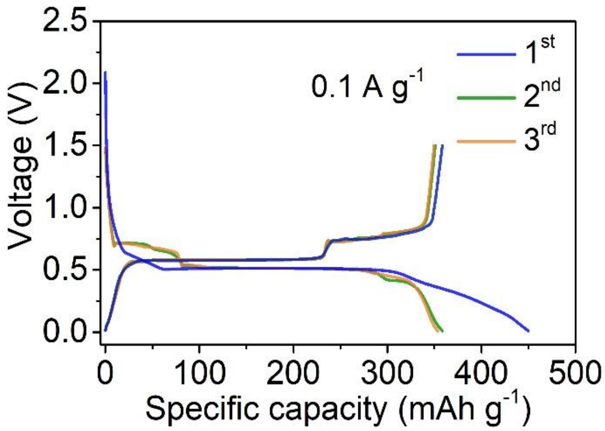

Fig. S17.
Galvanostatic charge-discharge curves at 0.1 A g^{-1} for the initial 3 cycles of HBiC anode.

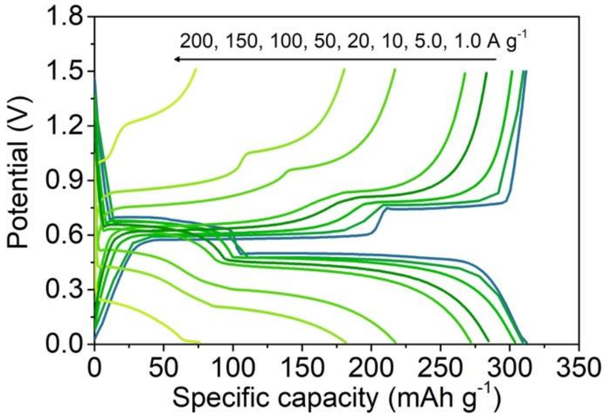

Fig. S18.
Charge-discharge curves of HBiC at different current densities.

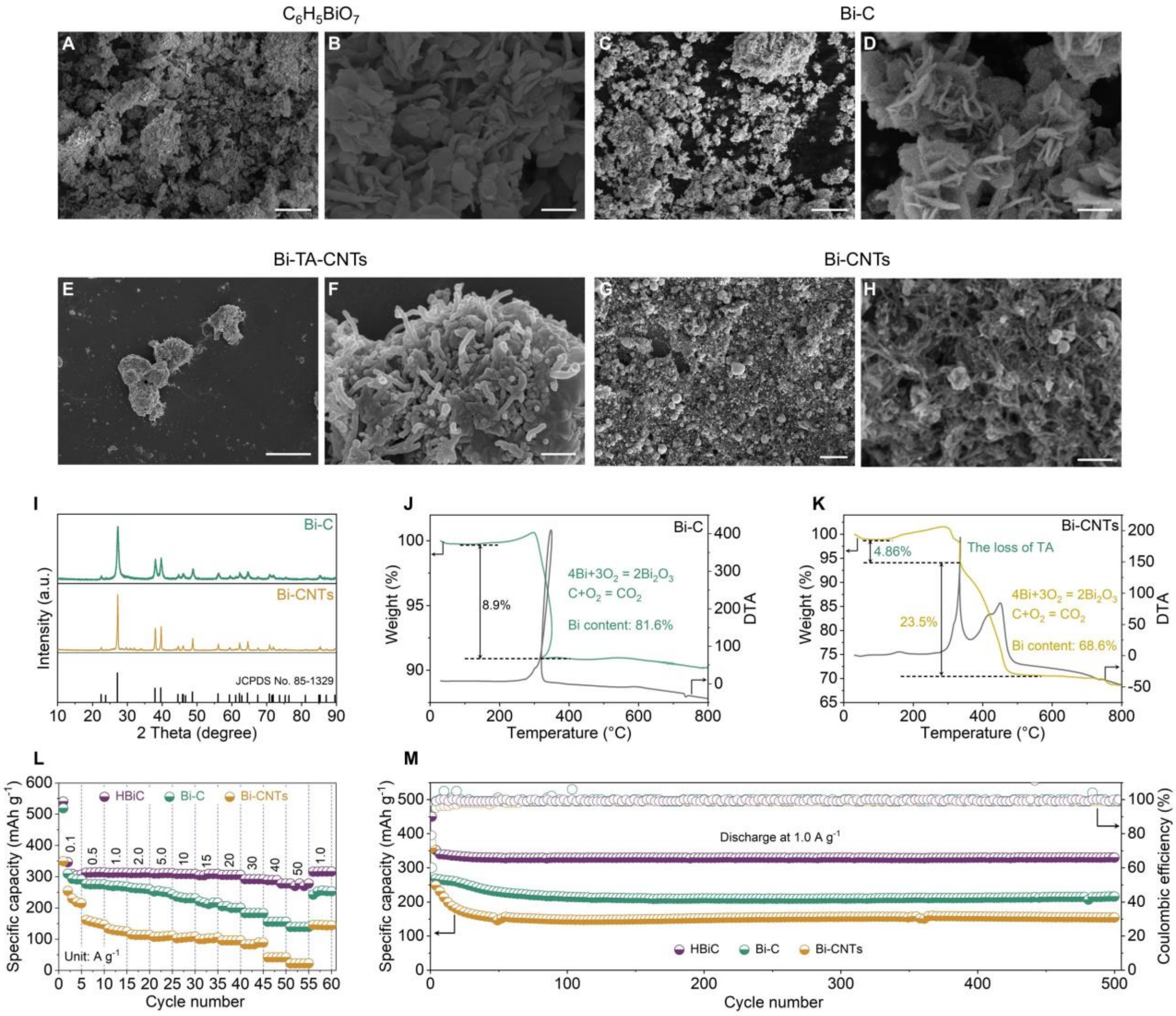

Fig. S19.

Morphology, phase, and sodium storage performance characterizations of different structured Bi-based materials. SEM images of (A and B) commercial bismuth citrate, (C and D) carbon sheets-based Bi (Bi-C) composite, (E and F) Bi-TA-CNTs, and (G and H) carbon nanotubes-based Bi (Bi-CNTs) composite. The disordered Bi-C and Bi-CNTs with simple ordered structure but without pores were prepared. (I) XRD patterns of Bi-C and Bi-CNTs. The sharp diffraction peaks in the XRD patterns suggested the existences of the hexagonal Bi phase both in Bi-C and Bi-CNTs. (J and K) TGA-DTA curves of Bi-C and Bi-CNTs. The Bi contents of Bi-C and Bi-CNTs were determined to be 81.6% and 68.6%, respectively. (L) Rate capabilities of HBiC, Bi-C, and Bi-CNTs at different current rates. The rate capabilities showed that the HBiC exhibited excellent capacities and stability at varied current density, while Bi-C and Bi-CNTs displayed low capacities at varied current density, even low to 138.9 and 22.2 mAh g^{-1} at a high current density of 50 A g^{-1}, respectively. (M) Cycling performances of HBiC, Bi-C, and Bi-CNTs at 1.0 A g^{-1}. Cycle performances of HBiC, Bi-C, and Bi-CNTs were obtained with capacities of 329.9, 215.7, and 154.1 mAh g^{-1} at a current density of 1.0 A g^{-1} after 500 cycles, respectively. These results indicated that the Bi-CNTs with simple ordered structure ensured fast electronic and ionic conductivity leading to a high initial capacity (357.7 mAh g^{-1}) similar to the theoretical capacity of Bi (385 mAh g^{-1}). However, due to the reduced order of Bi-CNTs, the

volume expansion of Bi nanoparticles during sodiation/desodiation processes could not be fully accommodated, thus resulting in a structure collapse and rapid decline of the cycle performance (reduced to 154.1 mAh g^{-1}) during the long-cycles (500 times). For Bi-C, although Bi-C had a higher Bi content (81.6%) than that of HBiC (67.1%), the Bi-C could not provide connectivity and fast electronic/ionic transport channels because of the lack of hierarchical structures, thus resulting in a dramatic decline in high-rate capacity and a decay of cycle performance. Scale bars are 5 μm (A, C, E, and G) and 500 nm (B, D, F, and H).

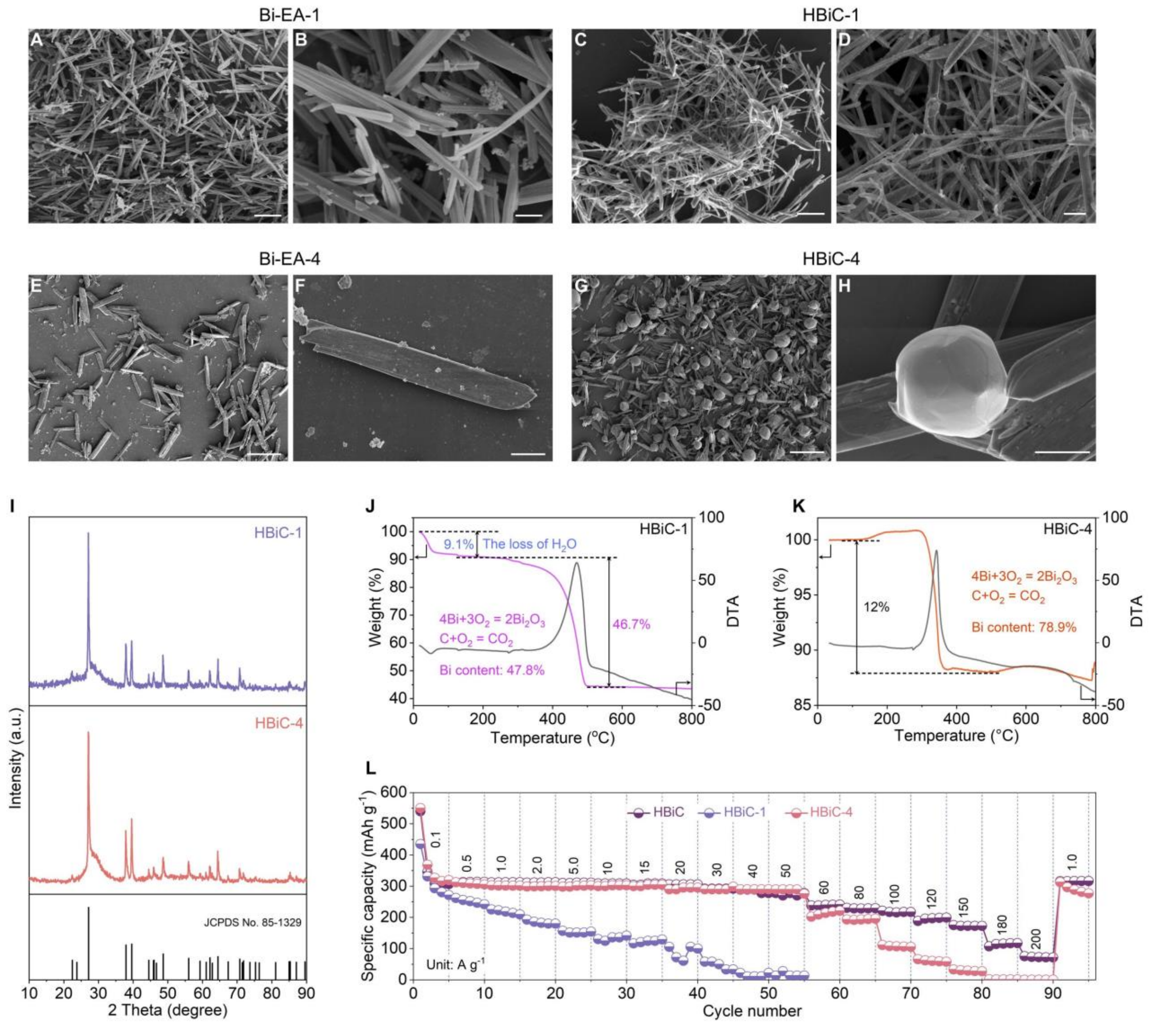

Fig. S20.

The effect of different Bi contents on morphology and electrochemical performances of HBiC. SEM images of Bi-EA mesocrystals with different molar ratios of Bi^{3+} ions to EA before and after carbonization. (A and B) Bi-EA-1, (C and D) HBiC-1, (E and F) Bi-EA-4, and (G and H) HBiC-4. The morphology of Bi-EA-1 mesocrystals and Bi-EA-4 mesocrystals were well inherited in the HBiC-1 and HBiC-4, similar to that of HBiC. (I) XRD patterns of HBiC-1 and HBiC-4. HBiC-1 and HBiC-4 also presented the characteristic peaks of the hexagonal Bi phase. (J and K) TGA-DTA curves of HBiC-1 and HBiC-4. The Bi contents of HBiC-1 and HBiC-4 were calculated to be 46.7% and 78.9%, respectively. (L) Rate capabilities of HBiC, HBiC-1, and HBiC-4 at different current densities. Compared with HBiC, the rate capabilities of HBiC-1 were inferior with increased current densities, and finally reached zero at the high current of 50 A g^{-1} because of the low Bi content. The initial capacity of HBiC-4 (550.2 mAh g^{-1}) was slightly higher than that of HBiC (540.1 mAh g^{-1}) at low current density of 0.1 A g^{-1}, owing to the high Bi content. However, when the current density was increased to 180 A g^{-1}, its capacity reduced to zero, which might be ascribed to the pulverization of the aggregated Bi particles (~10 − 20 μm) without the support of the hierarchically order structure. Scale bars are 5 μm (A, C, F, and H), 1 μm (B and D), and 50 μm (E and G).

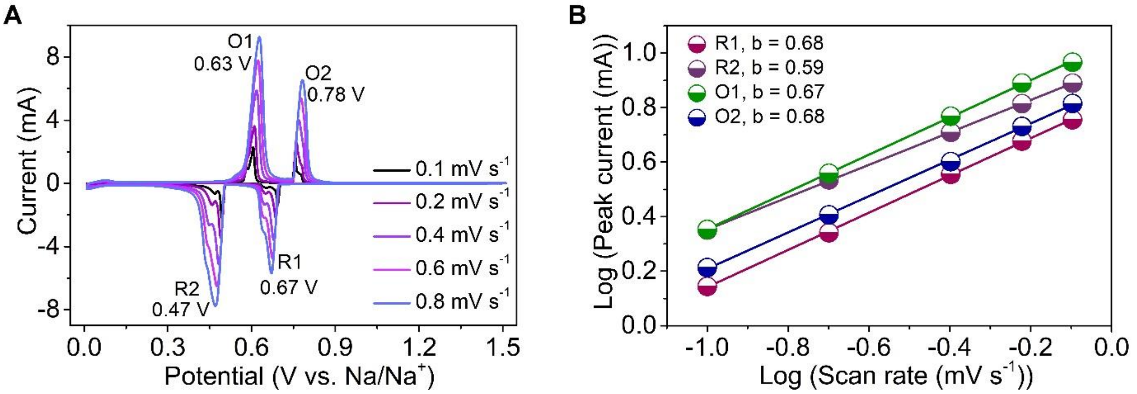

Fig. S21.

Kinetics process of Na storage mechanism of the HBiC electrode. (A) CV curves at different scan rates of 0.1, 0.2, 0.4, 0.6, and 0.8 mV s^{-1}. (B) Relationship between the peak currents and scan rates in logarithmic format. The redox peaks had similar shapes and peak potentials at increased scan rates, suggesting a small polarization voltage and fast reaction kinetics. The relationship between peak current (*i*, mA) and scan rate (*v*, mV s^{-1}) were analyzed by the power-law equation: $I = av^b$, where *a* and *b* were variables. As *b* values approached 0.5 or 1.0, a diffusion-controlled process (Faradaic) or a surface capacitance-dominated behavior was expected. When fitted two couples of redox peaks for the HBiC anode (R1/O1 and R2/O2), the *b*-values were 0.68/0.67 and 0.59/0.68 for R1/O1 and R2/O2, respectively, demonstrating that the redox process was Faradaic and depended on the diffusion of Na^+ ions.

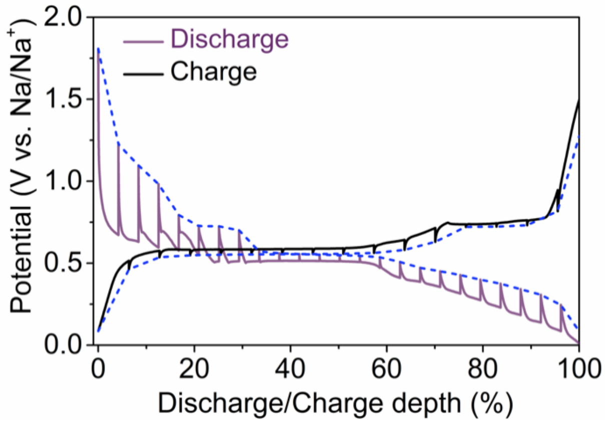

Fig. S22.
***Quasi*-equilibrium voltage curves of HBiC from galvanostatic intermittent titration technique (GITT) at 0.1 A g^{-1}.**

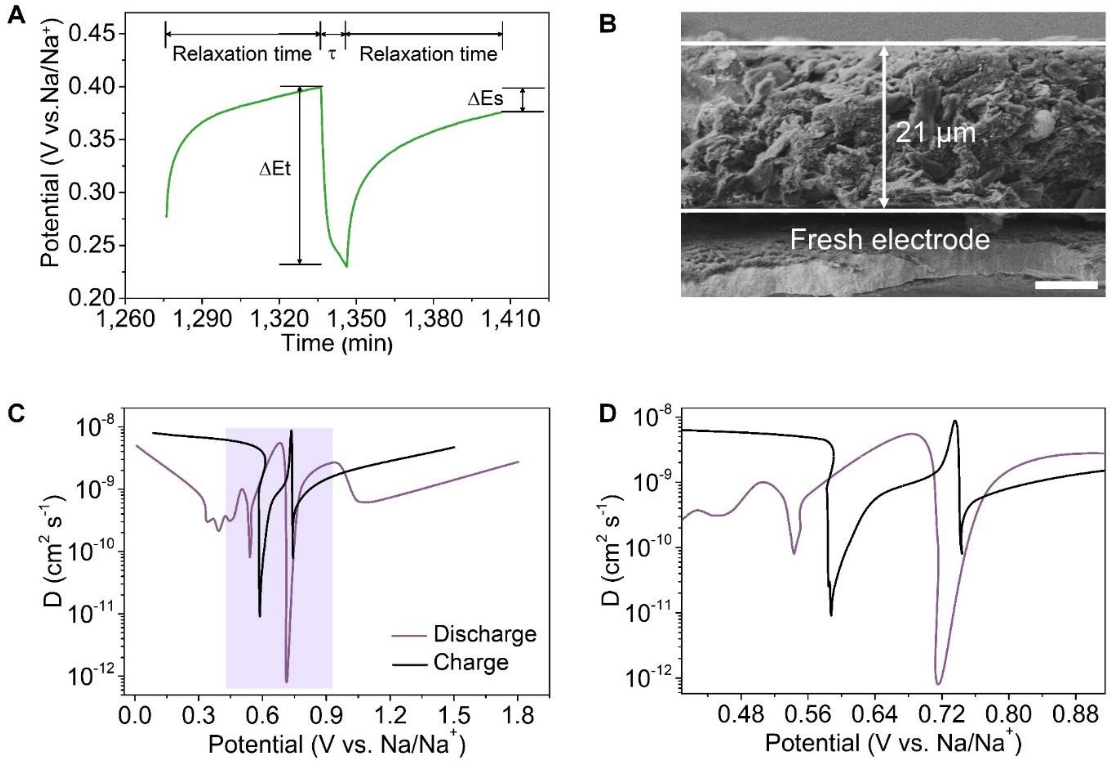

Fig. S23.

Sodium ions diffusion coefficient analysis of the HBiC electrode by GITT test. (A) A single titration process, which was composed of 10 min galvanostatic discharge-charge (pulse) at 0.1 A g^{-1} and followed by 60 min relaxation time. (B) Cross-sectional SEM image of HBiC electrode. (C and D) Na^+ diffusion coefficient vs. potential plot and corresponding enlarged profile of the selected area. The Na^+ diffusion coefficient was calculated as 1.36×10^{-9} cm^{-1} s^{-1} based on the following equation:

$$D = \frac{4L^2}{\pi\tau}\left(\frac{\Delta E_s}{\Delta E_t}\right)$$

Where t was the duration of the current pulse (s), τ was the relaxation time (s), ΔE_s was the steady-state potential change (V) by the current pulse, and ΔE_t was the potential change (V) during the constant current pulse. L was the sodium diffusion distance (cm), which was equal to the thickness of electrode. Scale bar is 5 μm (B).

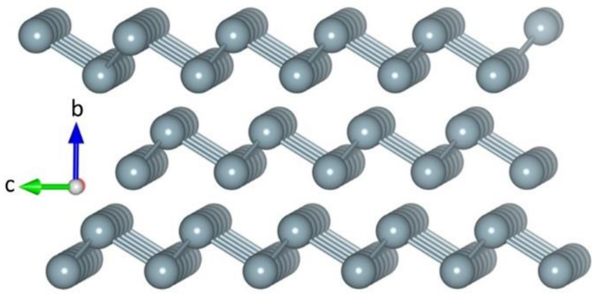

Fig. S24.
Structure model of the optimized (001) plane for Bi.

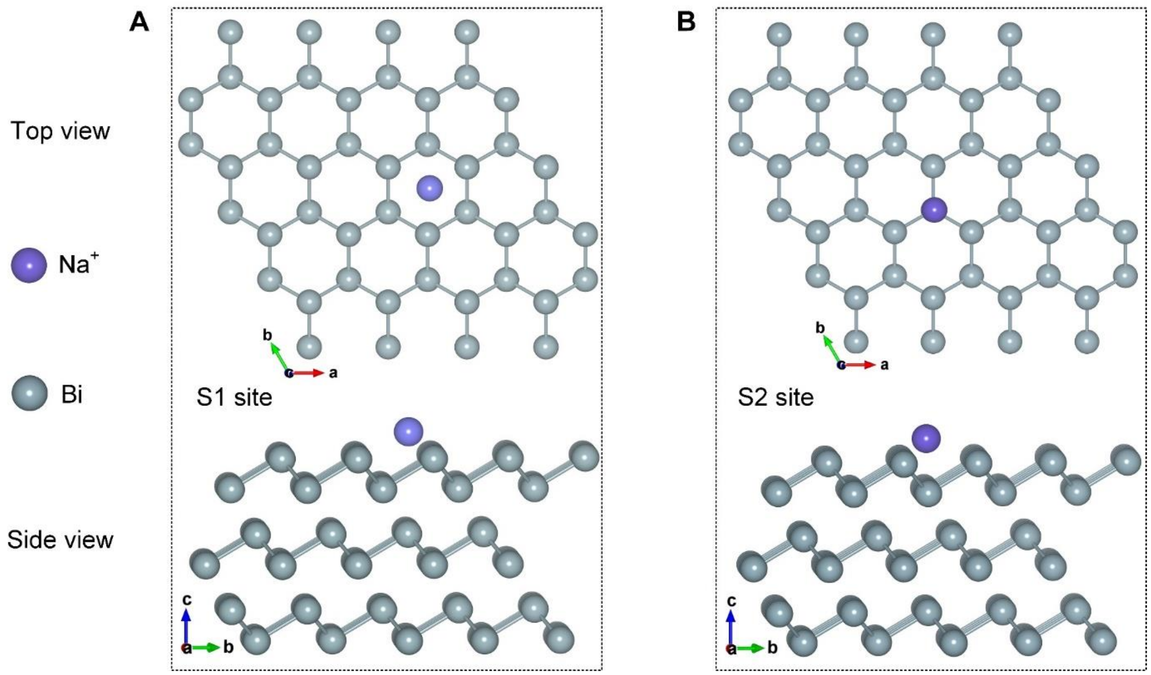

Fig. S25.
Top and side illustrations of simulations for a single Na^+ adsorbed on the Bi (001) plane. S1 site (above the center of the hexagonal ring) (A) and S2 site (on the top of Bi atom) (B).

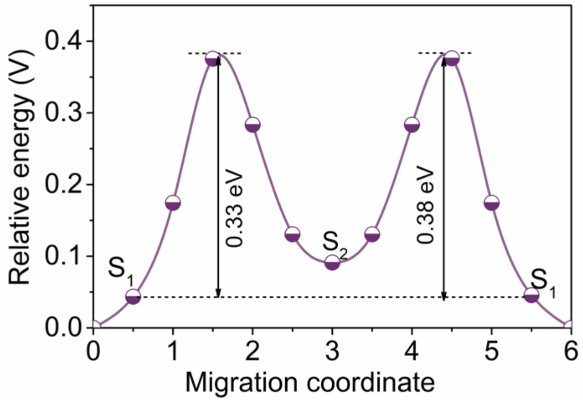

Fig. S26.
The curve of diffusion energy barriers of Na^+ through the selected diffusion pathway between bismuth layers.

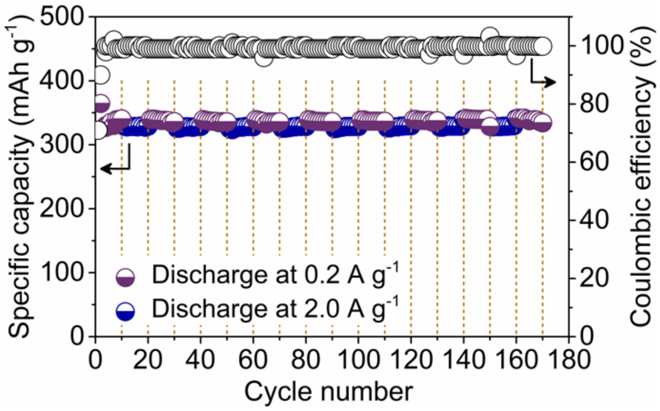

Fig. S27.

Specific capacities of HBiC under constant charge and discharge current densities of 2.0 A g^{-1} and 0.2 A g^{-1}, respectively.

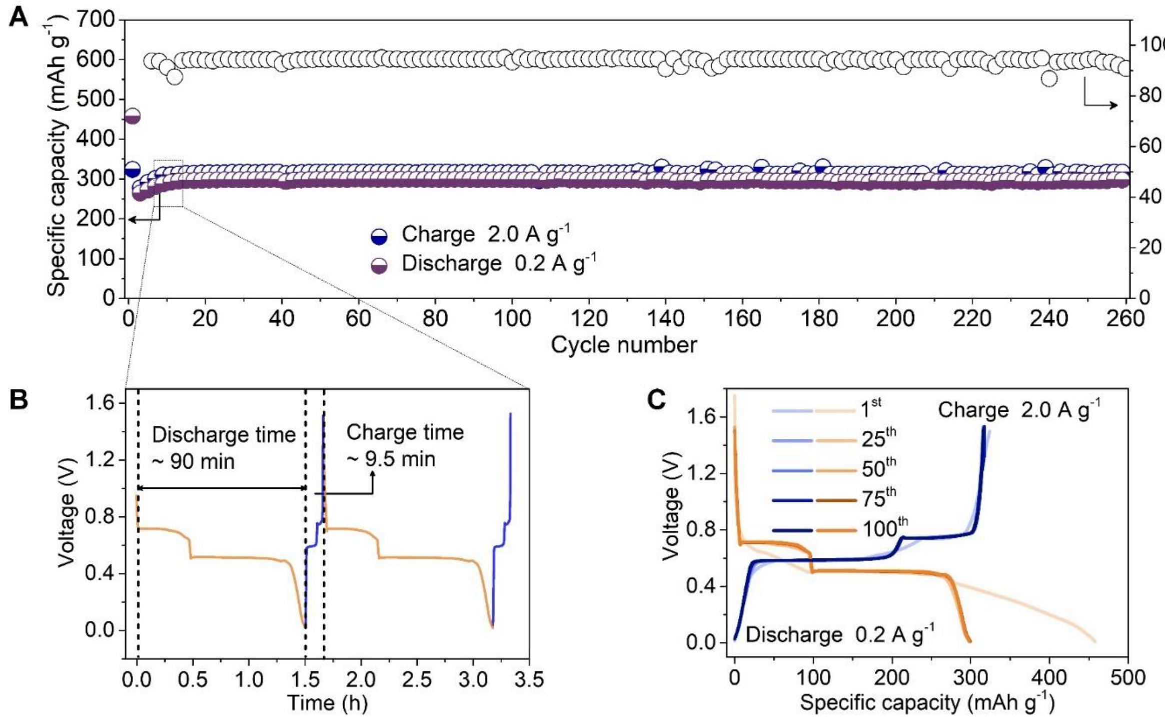

Fig. S28.

Electrode reversibility measurement under constant charge and discharge at current densities of 2.0 A g$^{–1}$ and 0.2 A g$^{–1}$. (A) Cycling performance test. The corresponding capacities still maintained at 312 and 295 mAh g$^{–1}$ after 250 cycles at 2.0 A g$^{–1}$ and 0.2 A g$^{–1}$, respectively. (B) The 6th and 7th cycles voltage-time curves. These curves suggested one complete galvanostatic charging at 2.0 A g$^{–1}$ took 9.5 min while the corresponding discharge duration at 0.2 A g$^{–1}$ reached 90 min. (C) The selected charge-discharge voltage profiles in 100 cycles for HBiC electrode. The charge-discharge voltage profiles were well overlapped in 100 cycles, indicating the stable platform and good reversibility of HBiC hybrid.

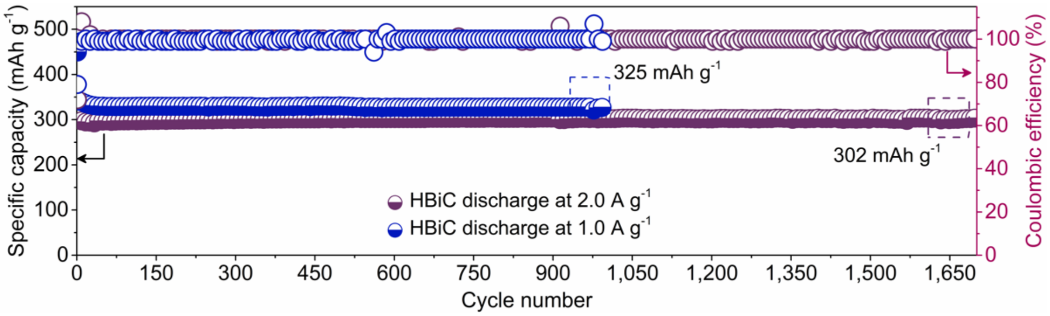

Fig. S29.

Cycling performance of HBiC at 1.0 and 2.0 A g^{-1}.

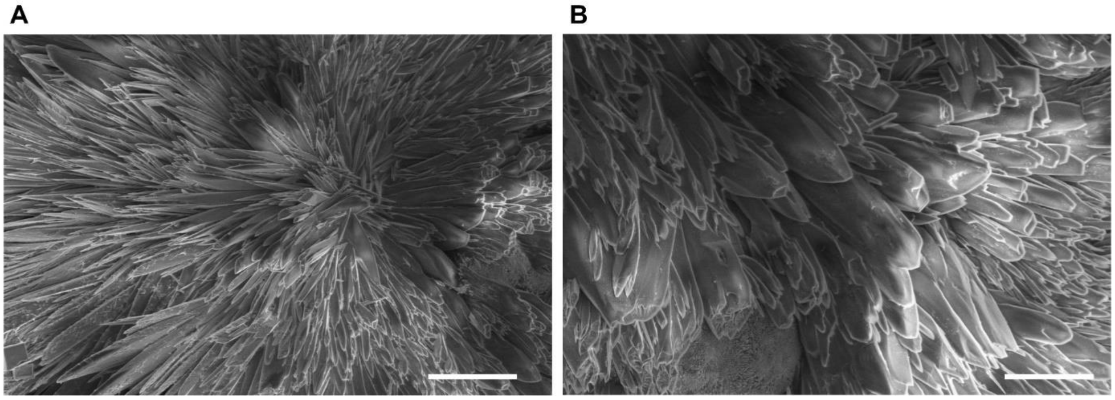

Fig. S30.
SEM images of HBiC electrode cycled at 2.0 A g^{-1} after 1,700 cycles. Scale bars are 20 μm (A) and 10 μm (B).

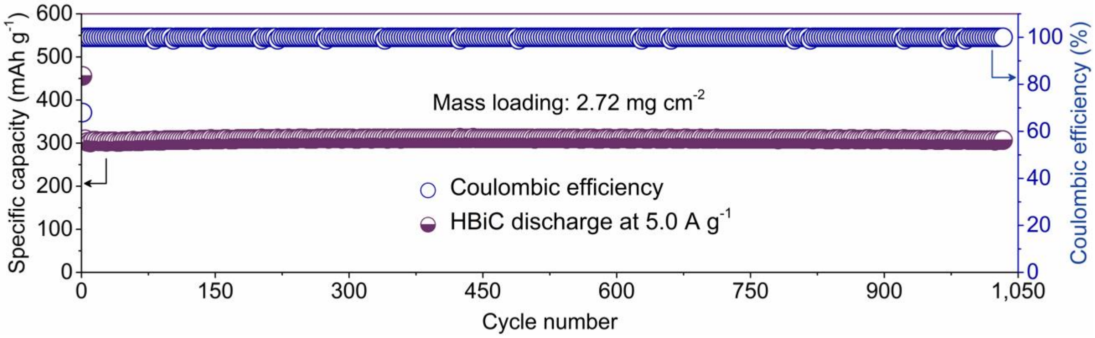

Fig. S31.
Cycling performance of HBiC with a mass loading of 2.72 mg cm^{-2} at 5.0 A g^{-1}.

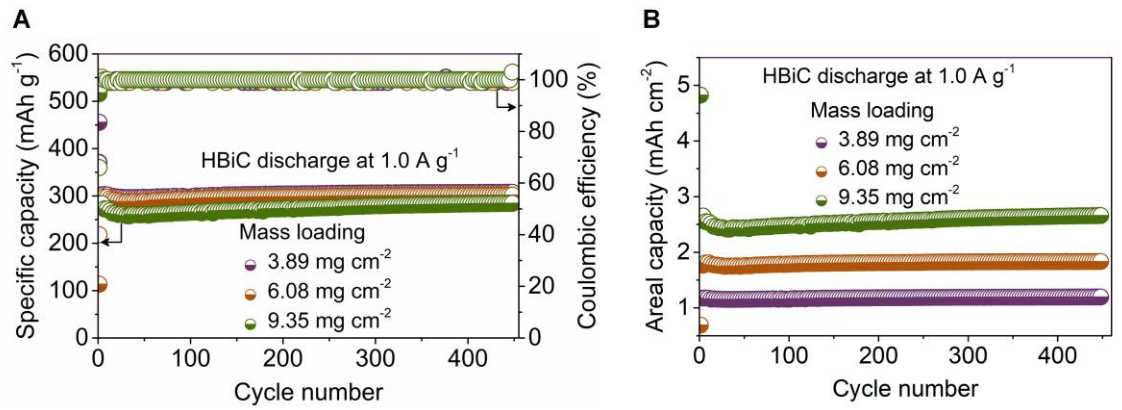

Fig. S32.

Electrochemical performances of HBiC at different mass loadings. (A) Cycling performance and (B) corresponding areal capacity with high mass loadings of 3.89, 6.08 and 9.35 mg cm^{-2}.

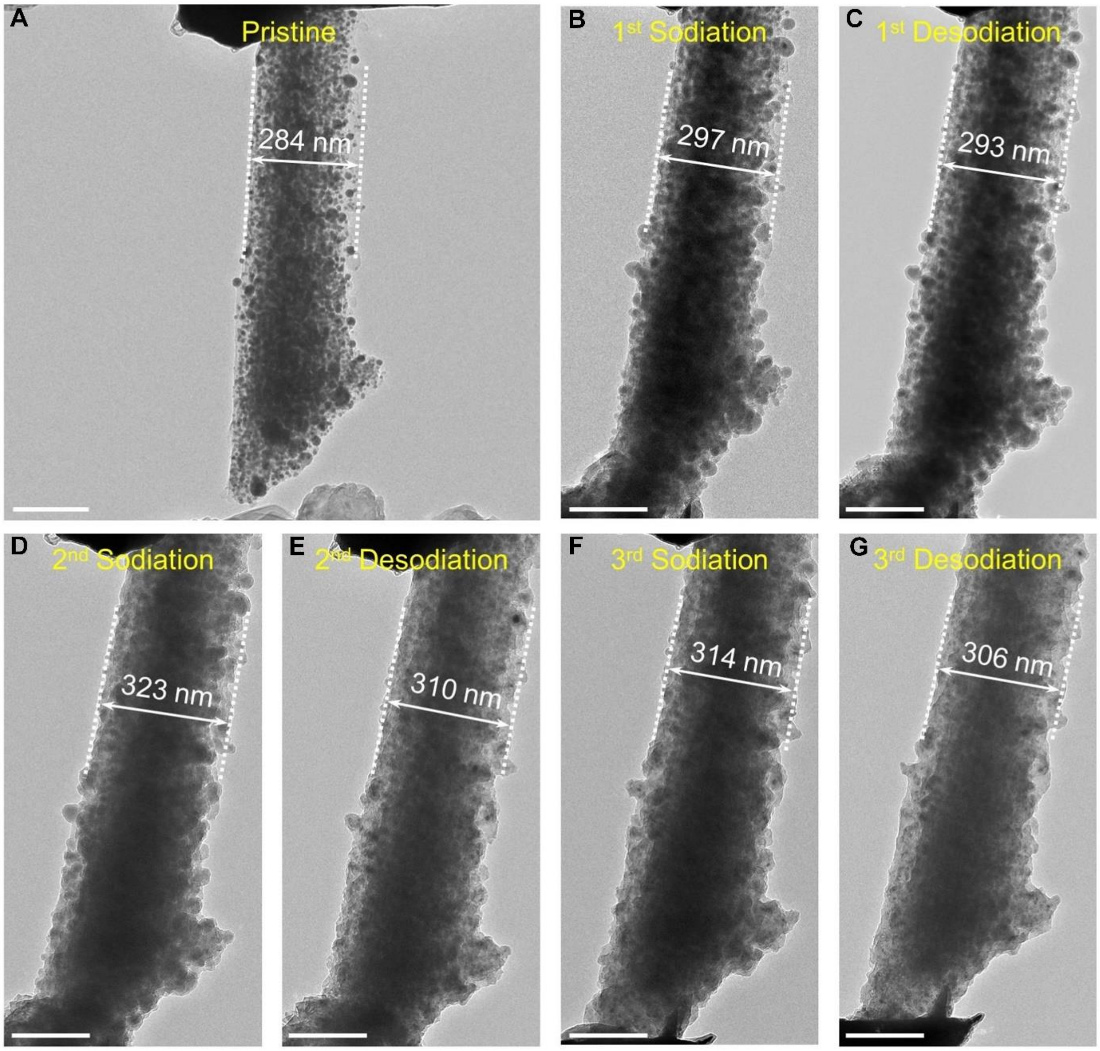

Fig. S33.
In-situ TEM images of HBiC at the pristine state and after different sodiation-desodiation cycles. The HBiC showed a gradual sodiation proceeded along the axial and radial direction, and the diameter of the HBiC slightly increased from 284 nm to 306 nm after three consequent sodiation-desodiation cycles. While the HBiC kept its structural integrity without mechanical degradation and cracking. Such robust structural integrity was greatly beneficial for the outstanding cycling performance. Scale bars are 200 nm (A to G).

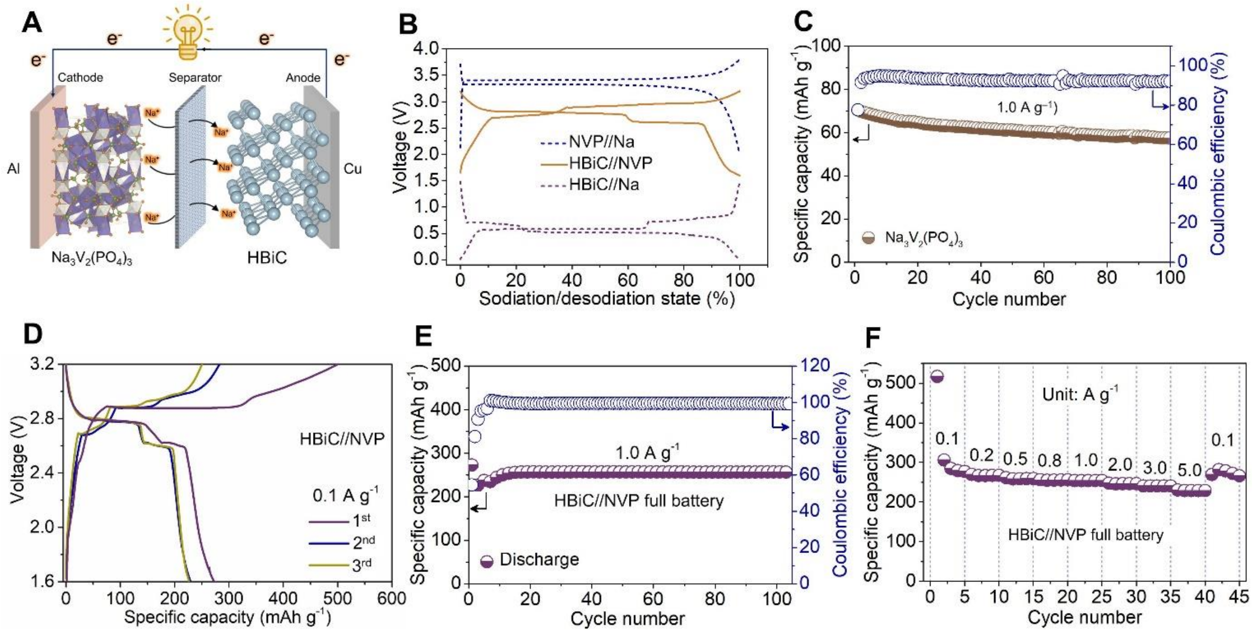

Fig. S34.

Electrochemical performances of full battery by pairing the HBiC anode with NVP cathode. (A) Working scheme. (B) Typical charge-discharge profiles of the NVP cathode, the HBiC anode and HBiC//NVP full cell. The HBiC//NVP full cell presented two voltage plateaus at 2.7 and 2.9 V in the charge cycle, corresponding to the voltage differences between NVP cathode (~3.4 V) and HBiC anode (0.7 and 0.5 V). (C) Cycling performance of NVP at 1.0 A g^{-1}. The NVP cathode existed a stable capacity of 60 mAh g^{-1} after 100 cycles. (D) Galvanostatic charge-discharge curves of HBiC//NVP full cell at 0.1 A g^{-1}. The overlapped charge-discharge profiles in selected cycles suggested a great electrochemical reversibility of HBiC//NVP full cell. (E and F) Cycling performance at 1.0 A g^{-1} and rate capability of HBiC//NVP full cell. The corresponding reversible capacity was maintained at 256 mAh g^{-1} after 100 cycles, with 100% capacity retention compared with the initial cycle.

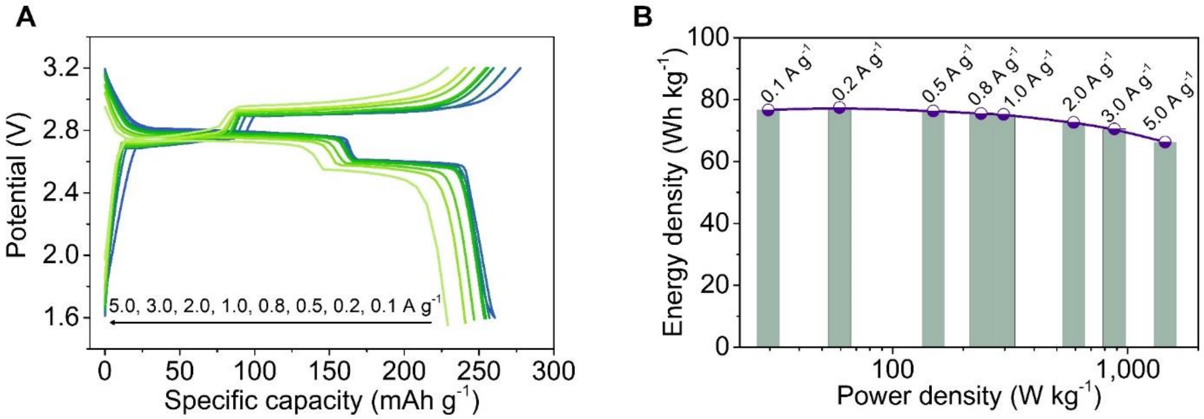

Fig. S35.
Electrochemical performance of HBiC//NVP full cell. (A) Charge-discharge curves at different rates. (B) Ragone plot (energy vs. power density), evaluated by the total mass.

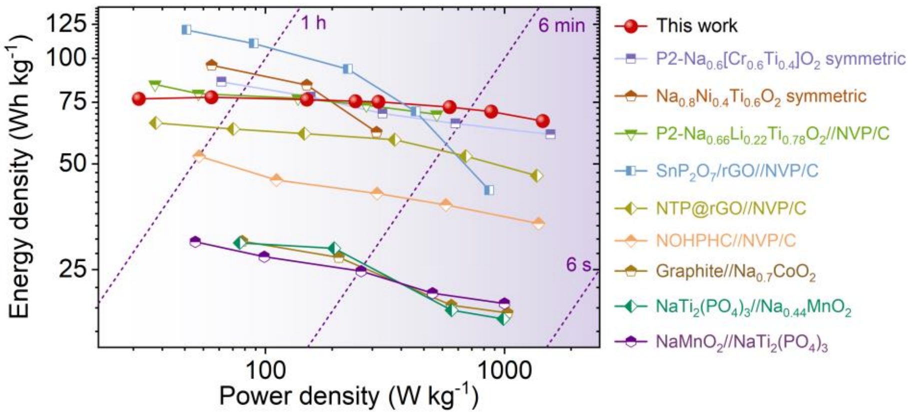

Fig. S36.

Ragone plot of the assembled HBiC//NVP full cell with comparisons to reported typical anode materials for SIBs: P2-$Na_{0.6}[Cr_{0.6}Ti_{0.4}]O_2$ symmetric (*48*), $Na_{0.8}Ni_{0.4}Ti_{0.6}O_2$ symmetric (*49*), P2-$Na_{0.66}Li_{0.22}Ti_{0.78}O_2$//NVP/C (*50*), SnP_2O_7/rGO//NVP/C (*51*), NTP@rGO//NVP/C (*52*), NOHPHC//NVP/C (*53*), Graphite//$Na_{0.7}CoO_2$ (*54*), $NaTi_2(PO_4)_3$//$Na_{0.44}MnO_2$ (*55*), $NaMnO_2$//$NaTi_2(PO_4)_3$ (*56*).

Table S1. ICP-OES results of Bi-EA mesocrystals before and after NaCl treatment.

Sample	Bi-EA mesocrystals before NaCl treatment	Bi-EA mesocrystals after NaCl treatment
Bi content (%)	46.3	47.3

Table S2. ICP-OES results of Bi-EA mesocrystals and HBiC.

Sample	Bi-EA mesocrystals	HBiC
Bi content (%)	46.3	67.1

Table S3. Comparison of the BET characteristic parameters determined by N_2 adsorption data of Bi-EA mesocrystals and HBiC.

	Bi-EA mesocrystals	HBiC
BET specific surface area	10.7 $m^2\ g^{-1}$	91.6 $m^2\ g^{-1}$
t-plot open surface area	10.7 $m^2\ g^{-1}$	20.678 $m^2\ g^{-1}$
Specific surface area contributed by micropores	0%	77.4%
Total pore volume	0.0322 $cm^3\ g^{-1}$	0.0836 $cm^3\ g^{-1}$
Micropore volume	0.002 $cm^3\ g^{-1}$	0.0317 $cm^3\ g^{-1}$
Micropore on total pore volume	6.2%	37.9%
Mesopore volume	0.0185 $cm^3\ g^{-1}$	0.0391 $cm^3\ g^{-1}$
Mesopore on total pore volume	57.4%	46.8%
Pore size	2.6 nm	3.6 nm

Table S4. Comparison of reported works on Bi-based electrodes for SIBs.

Electrode	Rate performance (mAh g^{-1}) at (Y) current density (mA g^{-1})	Cyclability (capacity retention (mAh g^{-1}) @ cycle number) at current density	First cycle Coulombic Efficiency	Reference
Bi@graphene	250 (1,280)	~110 @ 50 at 1,280 mA g^{-1}	55.5% @ 40mA g^{-1}	(*57*)
Bismuth nanorod bundle	102.3 (2,000)	302 @ 150 at 50 mA g^{-1}	55% @ 50 mA g^{-1}	(*58*)
Bi@C microsphere	83.4 (2,000)	123.5 @ 100 at 100 mA g^{-1}	45% @ 100 mA g^{-1}	(*59*)
Bi/CNF	85.6 (1,000)	186 @ 100 at 50 mA g^{-1}	53% @ 50 mA g^{-1}	(*60*)
Bi/CFC	120 (2,000)	350 @ 300 at 50 mA g^{-1}	61.2% @ 50 mA g^{-1}	(*61*)
Bi-NS@C	~110 (2,000)	106 @ 1,000 at 200 mA g^{-1}	——	(*62*)
Bulk Bi	356 (2,000)	389 @ 2,000 at 400 mA g^{-1}	94.8% @ 400 mA g^{-1}	(*35*)
Bi/Ni	206.4 (2,000)	302 @ 100 at 200 mA g^{-1}	——	(*63*)
Bi/C nanofibers	69.04 (3,200)	273.2 @ 500 at 100 mA g^{-1}	55.8% @ 100 mA g^{-1}	(*64*)
Bi@Graphite	113 (48,000)	~140 @ 10,000 at 3,200 mA g^{-1}	74.5% @ 80 mA g^{-1}	(*36*)
Bi@3DGFs	180 (50,000)	185.2 @ 2,000 at 10,000 mA g^{-1}	36% @ 100 mA g^{-1}	(*65*)
Bi@C	232 (60,000)	265 @ 30,000 at 8,000 mA g^{-1}	50.3% @ 800 mA g^{-1}	(*27*)
Bi@N-C	368 (2,000)	302 @ 1,000 at 1,000 mA g^{-1}	85.7% @ 50 mA g^{-1}	(*34*)
Bi@Void@C-2	173 (100,000)	198 @ 10,000 at 20,000 mA g^{-1}	46% @ 1,000 mA g^{-1}	(*66*)

Bi@N-C	178 (100,000)	235 @ 2,000 at 10,000 mA g^{-1}	36.5% @ 1,000 mA g^{-1}	(*33*)
Bi–C/CF	110 (2,400)	~340 @ 500 at 500 mA g^{-1}	——	(*67*)
FLB-G	263.2 (1,176)	317 @ 1,000 at 706 mA g^{-1}	——	(*68*)
**HBiC**	**72.5 (200,000)**	**263 @ 15,000 at 5,000 mA g^{-1}**	**79.9% @ 1,000 mA g^{-1}**	**This work**

Table S5. Lattice parameters and calculated surface energies of Bi with different orientations.

Surface	Lattice parameters (Å)	Surface energy ($J\ m^{-2}$)
(001)	a = 9.92, b = 9.92	0.18
(101)	a = 14.94, b = 9.92	0.26
(110)	a = 11.86, b = 15.74	0.39
(111)	a = 14.94, b = 14.94	0.35
(211)	a=19.71, b = 14.94	0.31
(221)	a = 15.74, b = 25.41	0.34
(201)	a = 25.40, b = 9.09	0.40
(210)	a = 11.86, b = 24.03	0.33
(212)	a = 14.97, b = 21.72	0.49
(102)	a = 19.71, b = 9.09	0.57

Table S6. Adsorption energy of Na^+ on the two stable adsorption sites of the Bi (001) crystal plane.

Site	Distance d (Å)	Adsorption energy (eV)
S1	2.29	−0.67
S2	2.38	−0.45

Captions for Supplementary Movies

Movie S1.
Time-resolved TEM observation of HBiC during the initial sodiation-desodiation process.

Movie S2.
In-situ TEM observation of HBiC during three sodiation-desodiation cycles.
