# Supplementary material for: Superstructured mesocrystals through multiple inherent molecular interactions for highly reversible sodium ion batteries
Source: Sci Adv. 2021 Sep 8;7(37):eabh3482. doi: 10.1126/sciadv.abh3482 (PMC8442931; doi:10.1126/sciadv.abh3482)
Supplement: Supplementary file 4 — Correction 15 April 2022: The authors inadvertently included the wrong image for Supplemental Figure S9B. This panel was replaced with the correct image in the Supplemental Materials PDF. The original version is available here: [file sciadv.abh3482_sm.v1.pdf]

Science Advances
AAAS

Supplementary Materials for

Superstructured mesocrystals through multiple inherent molecular interactions for highly reversible sodium ion batteries

Xiaoling Qiu, Xiaoling Wang, Yunxiang He, Jieying Liang, Kang Liang, Blaise L. Tardy, Joseph J. Richardson, Ming Hu, Hao Wu*, Yun Zhang*, Orlando J. Rojas, Ian Manners, Junling Guo*

*Corresponding author. Email: hao.wu@scu.edu.cn (H.W.); y_zhang@scu.edu.cn (Y.Z.); junling.guo@scu.edu.cn, junlingguo@g.harvard.edu (J.G.)

Published 8 September 2021, *Sci. Adv.* **7**, eabh3482 (2021)
DOI: 10.1126/sciadv.abh3482

The PDF file includes:

Figs. S1 to S36
Tables S1 to S6
Legends for movies S1 and S2
References

Other Supplementary Material for this manuscript includes the following:

Movies S1 and S2

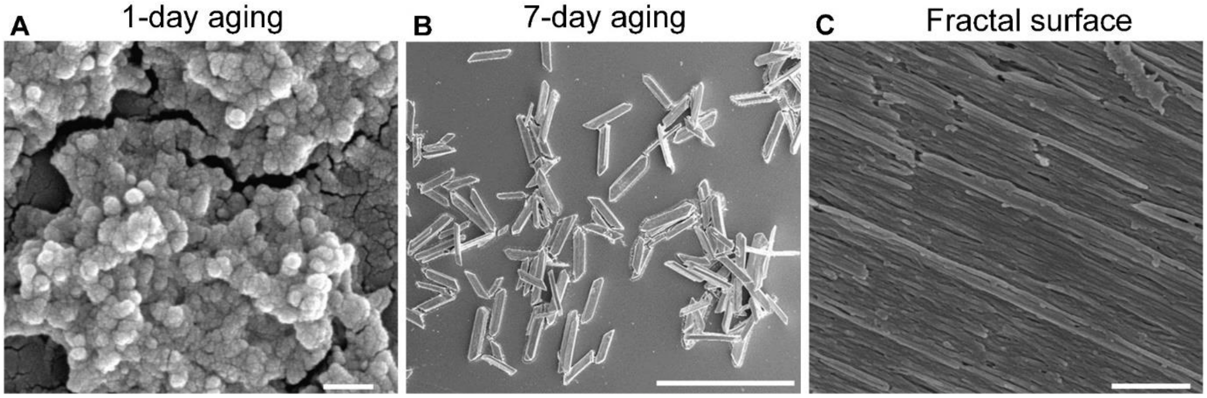

Fig. S1.
SEM characterization of Bi-EA mesocrystal formation. (A to C) SEM images of Bi-EA subunits after 1-day aging, mesocrystals after 7-day aging and fractal rough surface of mesocrystals. Scale bars are 200 nm (A), 200 μm (B) and 500 nm (C).

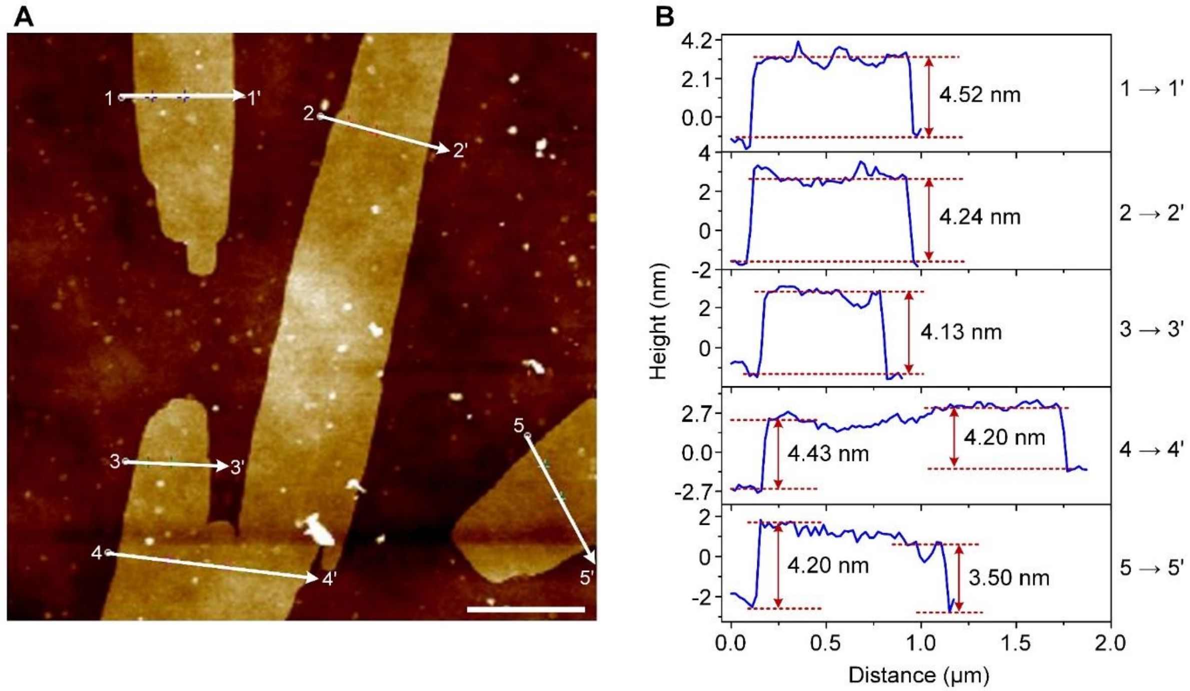

Fig. S2.

AFM image and corresponding height profiles of Bi-EA mesocrystals. Samples for AFM images were prepared by sonication in a water-bath at 23°C for 30 min with a mesocrystal suspension concentration of 0.5 mg mL^{-1}. The thin layers shown a height ~4.2 nm, which suggested the existence of subunits of filament structures. Scale bar is 1 μm (A).

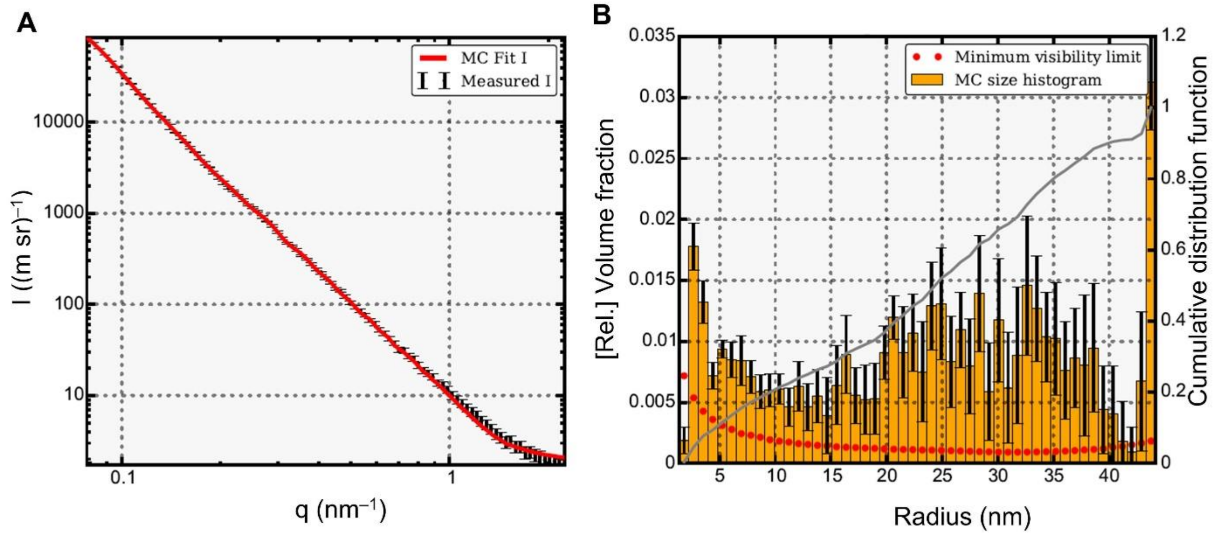

Fig. S3.

SAXS characterization of the Bi-EA mesocrystals and the model fitting for hierarchical structure analysis. (A) SAXS plot of the intensity (counts) vs. q (Å^{-1}) with measured data (black) and model fitted data (red). The collected data showed a power law slope and indicated the prepared sample was aggregated and polydisperse in size. (B) Distribution of the filament diameter by fitting with sphere model from SAXS data. The model fitting data suggested a distribution of the filament diameter from 1.8 − 44.2 nm. This suggested the polydispersity of the prepared sample, in which the subunits assembled randomly into filaments.

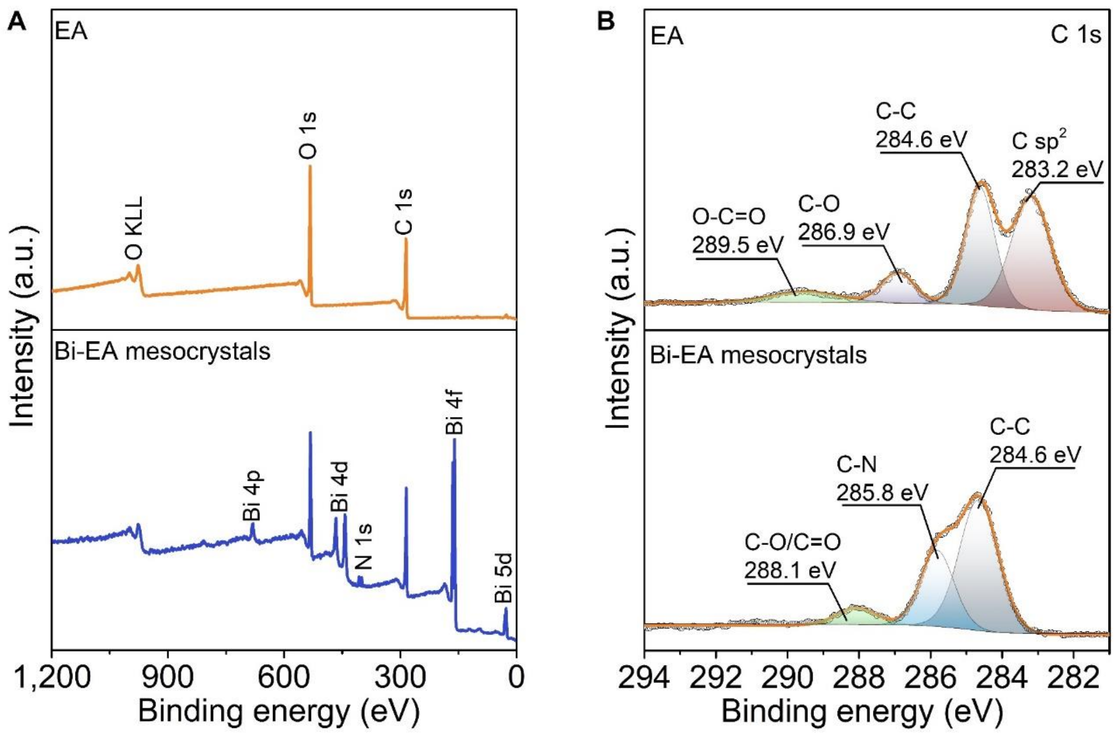

Fig. S4.
XPS analysis of EA and Bi-EA mesocrystals. (A) XPS survey spectra. (B) High-resolution XPS spectra of C 1s. A new C-N peak (285.8 eV) in C 1s spectra and the peak shifted and merged of C-O, C=O, and C-C bond, confirmed the coordination among EA, Bi^{3+} ions, and NMP molecules。

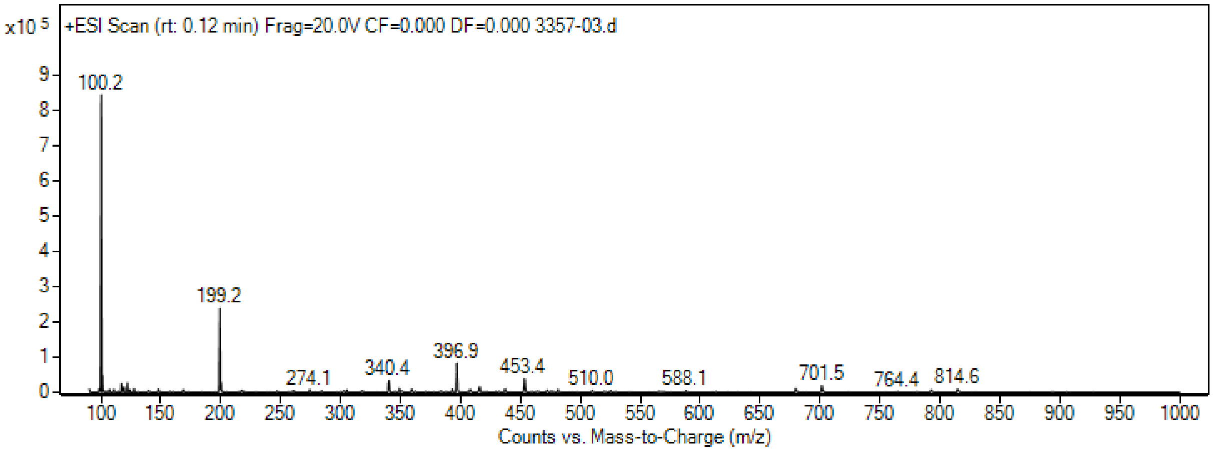

Fig. S5.

Mass spectrum of Bi-EA mesocrystals by ESI-MS. The fragment peak of 100.2 indicated the presence of NMP molecules in the mesocrystals.

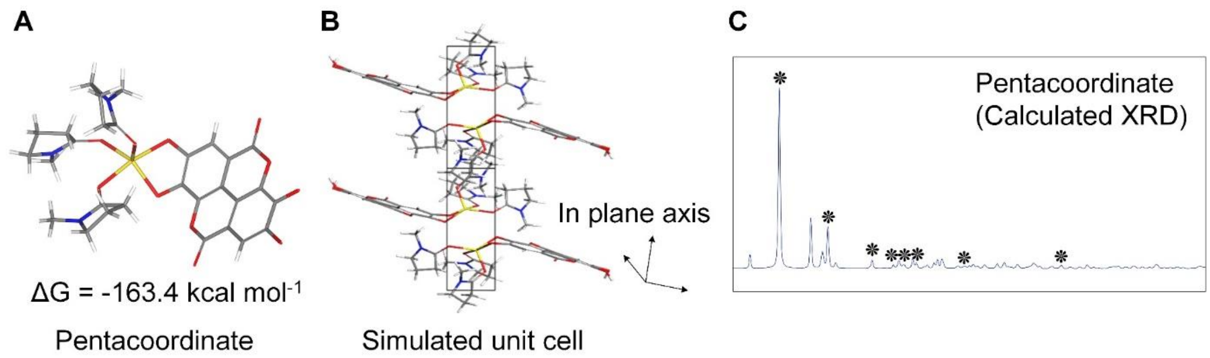

Fig. S6.
Simulation studies of the EA-Bi mesocrystals with penta-coordination primary structure. (A and B) Computational optimized pentacoordinate structure and corresponding simulated unit cell. (C) Calculated XRD pattern of pentacoordinate. The calculated XRD pattern of simulated pentacoordinate crystal unit cell presented identical peaks with the experimental ones, which suggested the presence of pentacoordinate structure characteristic, such as NMP solvent interactions in the mesocrystal.

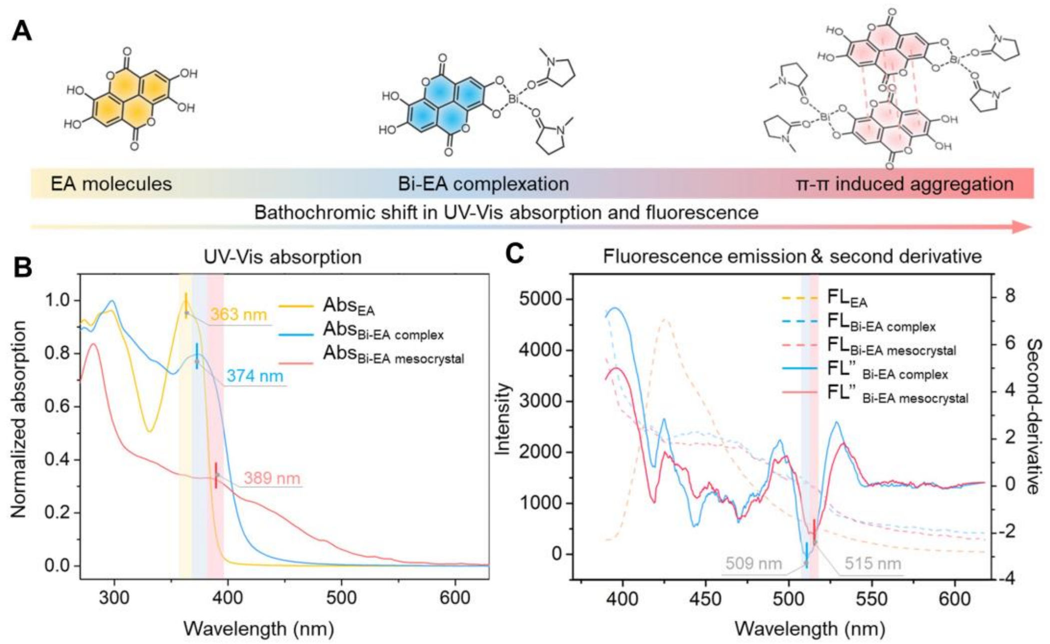

Fig. S7.

UV-vis absorption and fluorescence emission studies on the secondary structure of Bi-EA mesocrystals. (A) Structures of EA molecule, a Bi-EA complex, and π-π induced aggregation of Bi-EA complexes. (B) UV-Vis absorption spectra exhibited a bathochromic shift from 363 nm (EA molecule) to 374 nm (Bi-EA complex) due to complexation, followed by a further bathochromic shift to 389 nm (Bi-EA mesocrystal) due to π-π interactions. (C) Fluorescence (FL) spectra of EA, Bi-EA complex, and π-π induced aggregation of Bi-EA complexes, where a decreased fluorescent intensity of Bi-EA complex and Bi-EA mesocrystal compared with that of the EA molecule could be observed. Secondary-derivative spectra of Bi-EA complex and Bi-EA mesocrystal exhibited a bathochromic shift from 509 to 515 nm suggesting the formation of π-π induced aggregation.

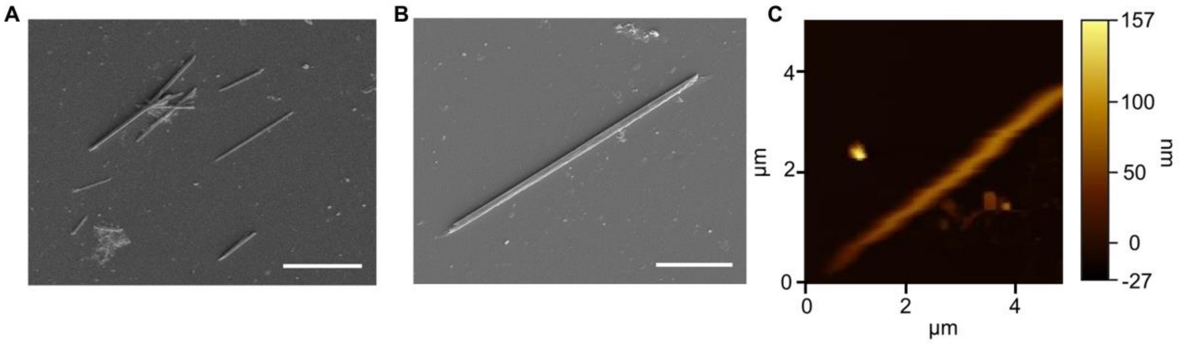

Fig. S8.
Morphological characterization of Bi-EA mesocrystals after NaCl treatment. (A and B) SEM and (C) AFM images of Bi-EA mesocrystals after NaCl treatment (200 mg mL^{-1}). Scale bars are 25 μm (A) and 10 μm (B).

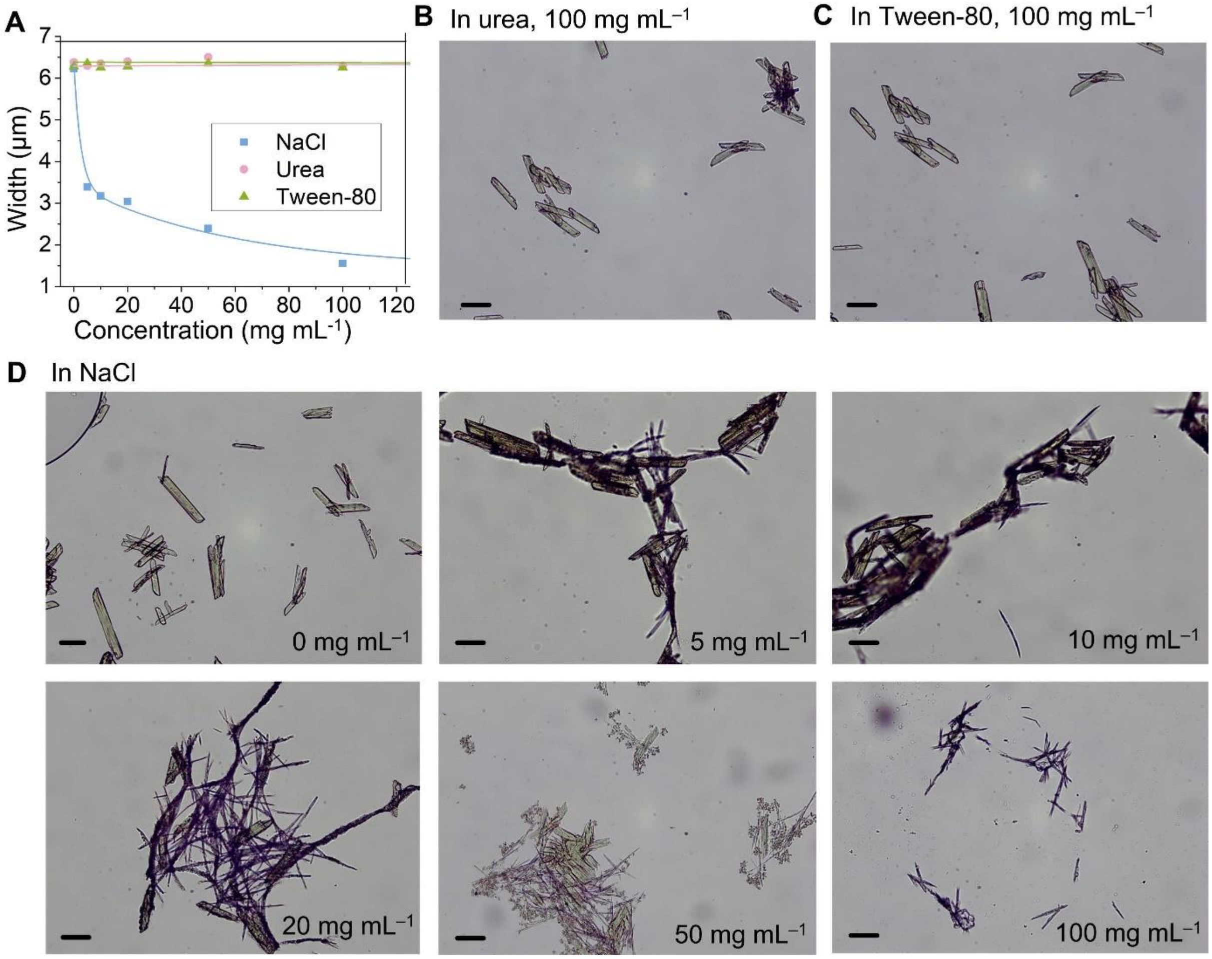

Fig. S9.

Disassembly of mesocrystals (5 mg mL^{-1} suspension in water) with additives in aqueous solution with the incubation at 37°C for 1 h. (A) Plot of width (μm) vs. additive concentration (mg mL^{-1}). (B to D) Optical microscope images of mesocrystals in urea (100 mg mL^{-1}), Tween-80 (100 mg mL^{-1}) and NaCl (with varied concentration of 0 − 100 mg mL^{-1}). A disassembly process of mesocrystals into filaments in NaCl solution was observed with an increase of concentration. The results suggested that the assembly of filaments was driven by ionic interactions. Scale bars are 20 μm (B to D).

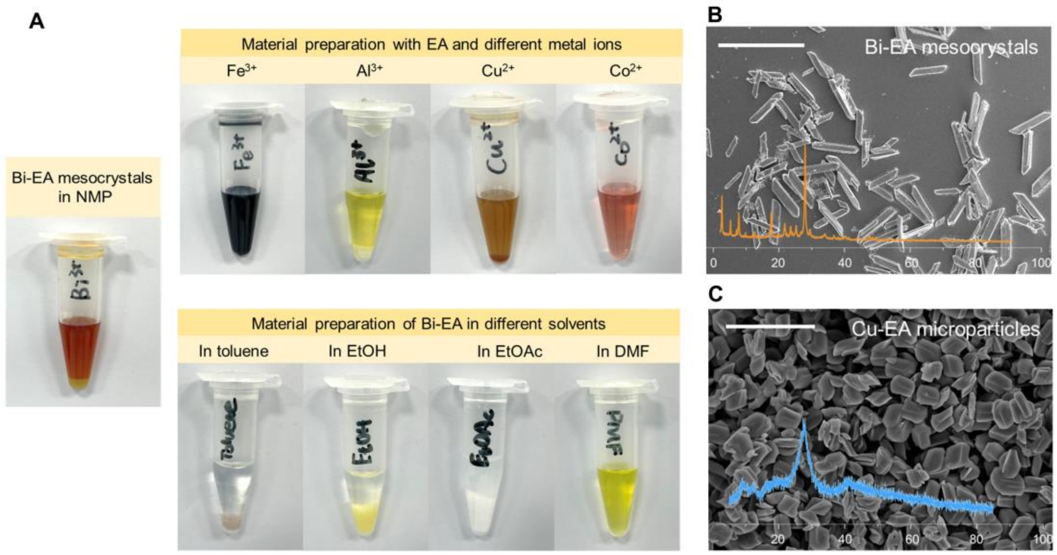

Fig. S10.

Metal ions and solvents screen for the mesocrystal preparation. (A) Photo images of samples prepared with different metal ions and solvents. For different cations, no precipitation or mesocrystal formation was seen with the usage of Fe^{3+} and Co^{2+} ions. In addition, Al^{3+} salts were not soluble in NMP, while the precipitation collected with Cu^{2+} ions had low crystallinity (C). For different solvents, either Bi^{3+} ions or EA were not soluble in toluene, EtOAc, and EtOH, while no precipitation was collected with DMF. (B and C) SEM images and XRD patterns of Bi-EA mesocrystals and Cu-EA microparticles. The morphology and crystallinity of the Cu-EA microparticles was different from that of the Bi-EA mesocrystals. Scale bars are 200 µm (B) and 5 µm (C). Photo credit (A): Xiaoling Qiu, Sichuan University.

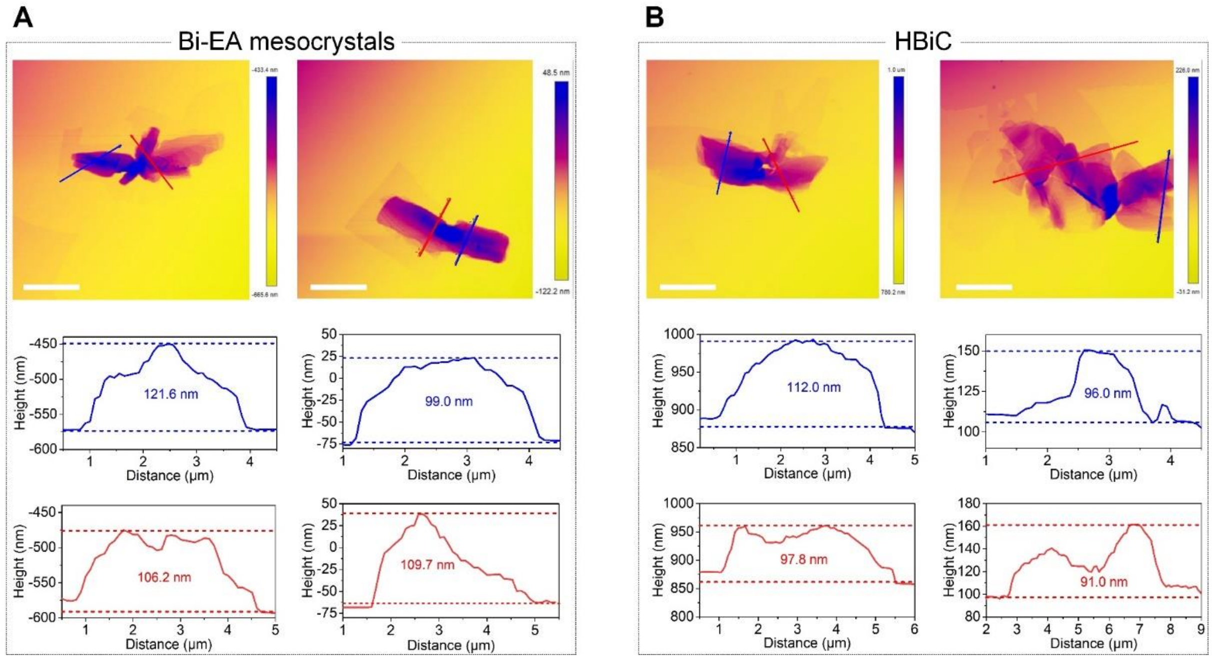

Fig. S11.

AFM images and corresponding height profiles of Bi-EA mesocrystals and HBiC hybrid. Scale bars are 5 μm (A and B).

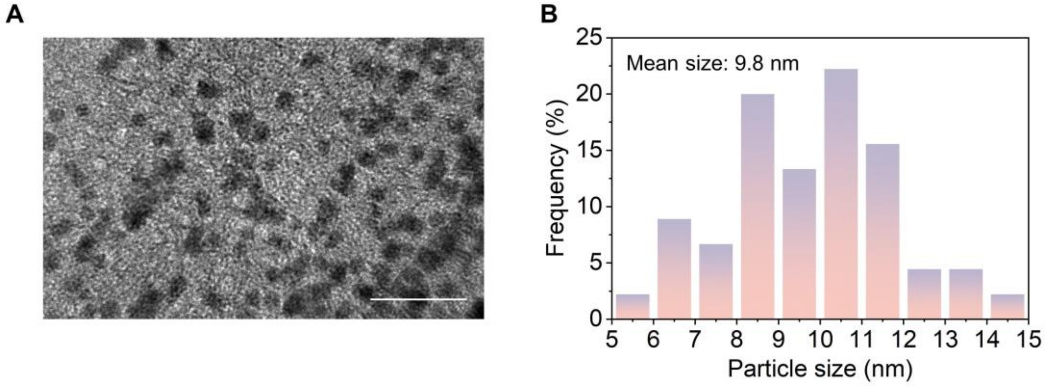

Fig. S12.
Particle size analysis of the Bi nanoparticles embedded in HBiC. (A) TEM image and (B) corresponding particle size distribution. Scale bar is 50 nm (A).

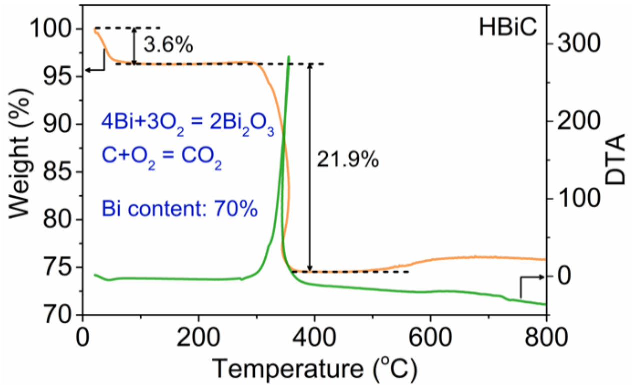

Fig. S13.
TGA curve (in air) of the HBiC. The Bi content was calculated based on the following equation and the weight content of Bi was 70%.

$$Bi\ (wt\%) = 100 \times \frac{2 \times molecular\ weight\ of\ Bi}{molecular\ weight\ of\ Bi_2O_3} \times \frac{final\ weight\ of\ Bi_2O_3}{initial\ weight\ of\ HBiC}$$

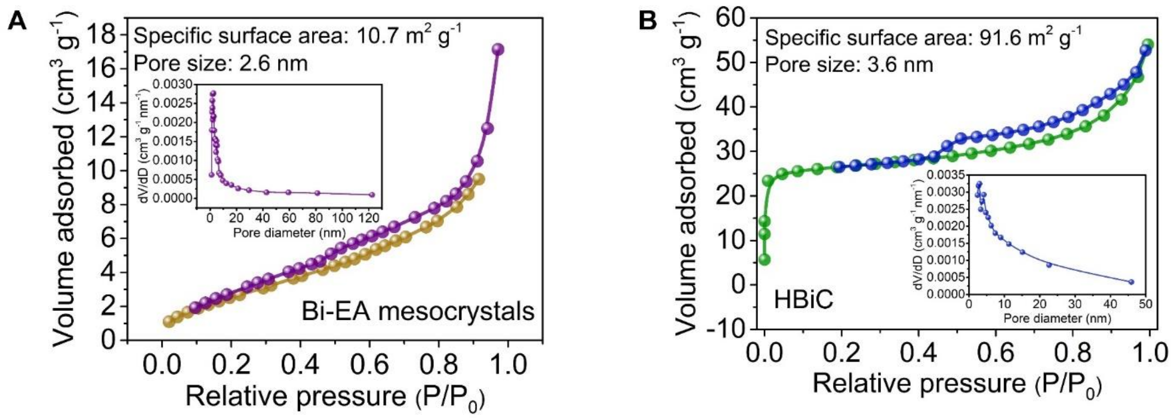

Fig. S14.

BET measurements of Bi-EA mesocrystals and HBiC. N_2 adsorption-desorption isotherms and pore size distributions (insets) of Bi-EA mesocrystals (A) and HBiC (B). As calculation from the adsorption isotherms using the BET theory and t-plots, the BET characteristic parameters determined by N_2 adsorption data of Bi-EA mesocrystals and HBiC were further showed in **table S3**. The BET specific surface area and total pore volume of HBiC were calculated to be 91.6 $m^2\ g^{-1}$ and 0.0836 $cm^3\ g^{-1}$, respectively, which is larger than the value of Bi-EA mesocrystals (10.7 $m^2\ g^{-1}$ and 0.0322 $cm^3\ g^{-1}$). The increased specific surface area and pore volume should be ascribed to the additional defects induced by the carbothermic reduction. Bi-EA mesocrystals showed pore size distribution range from 1.15 to 122.53 nm with an average pore of 2.6 nm, while the HBiC exhibited pores centered from 0.18 to 210.99 nm with an average pore size of 3.6 nm, indicating the micropores, mesopores, and macropores coexist in these two samples. Moreover, the Bi-EA mesocrystals showed no or negligible micropore (smaller than 2 nm diameter) volumes, while the HBiC showed high proportions of micropore specific surface area (77.4%) and micropore volume (37.9%) of the total N_2 adsorption isothermal. Accordingly, the electrolyte could freely get into the macropores possessing the sizes of > 50 nm, or even micron-size (2 − 50 nm), and infiltrated into the whole structure of HBiC easily. This hierarchical porous structure could increase the contact areas between the electrode and electrolyte and decrease the diffusion distance, which was in favor of the fast transportation of Na^+ ions.

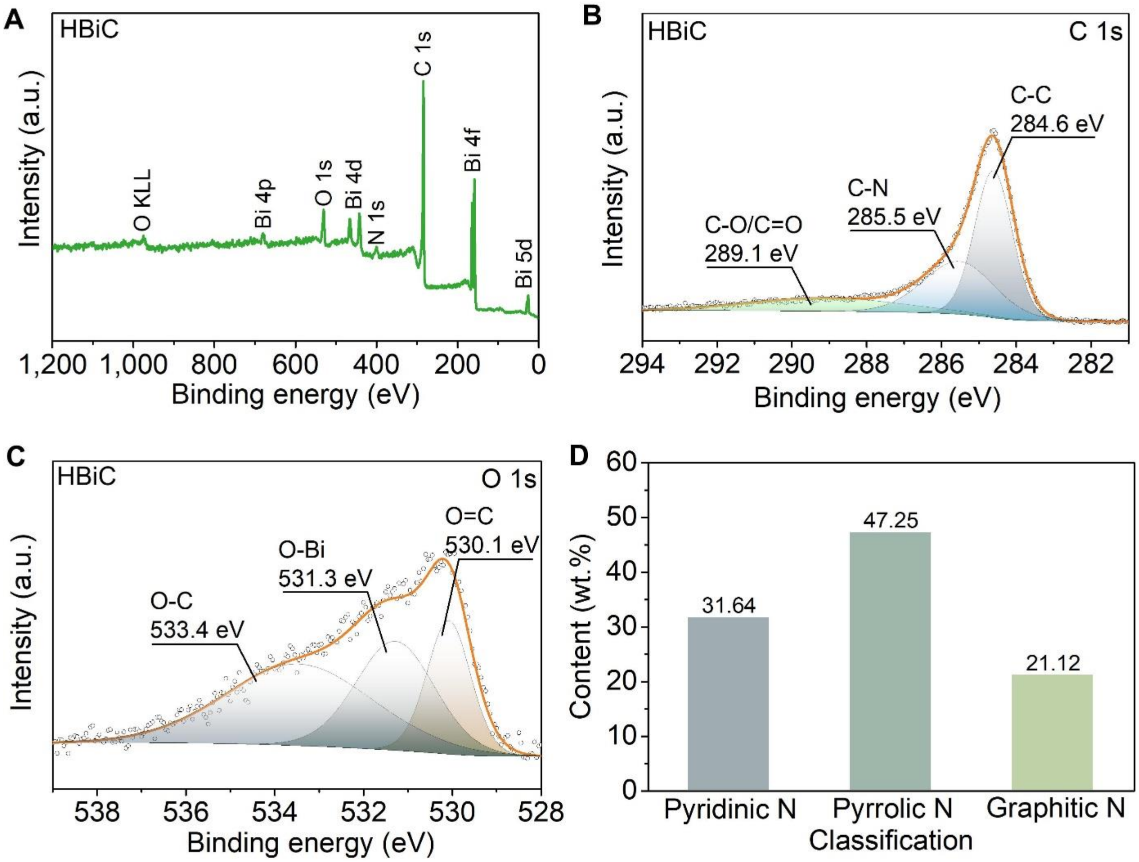

Fig. S15.
XPS analysis of HBiC. (A) XPS survey spectrum. (B and C) High-resolution spectra of C 1s and O 1s. The peaks for C-O/C=O bond (289.1 eV) in C 1s spectra and O-Bi bond (531.3 eV) in O 1s spectra suggested the existence of Bi-O-C bonds in the HBiC hybrid. (D) The relative contents of pyridinic N, pyrrolic N and graphitic N in N 1s spectra. The nitrogen content in the *N*-doped carbon was 5.36%, and this *N*-doped carbon matrix had been certified to enrich the Na^+ trapping defects, thus enhancing the sodium storage capabilities.

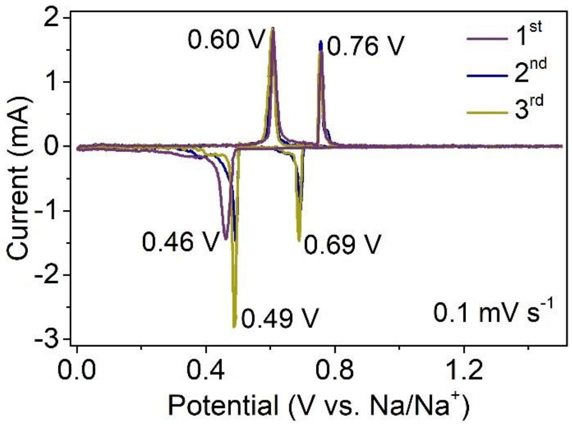

Fig. S16.

CV curves at a scan rate of 0.1 mV s^{-1} of HBiC anode.

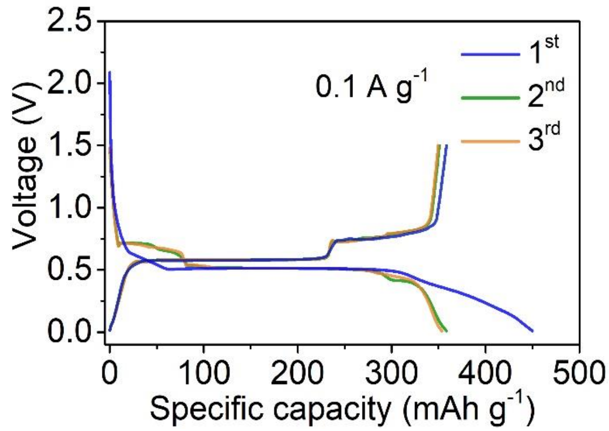

Fig. S17.

Galvanostatic charge-discharge curves at 0.1 A g^{-1} for the initial 3 cycles of HBiC anode.

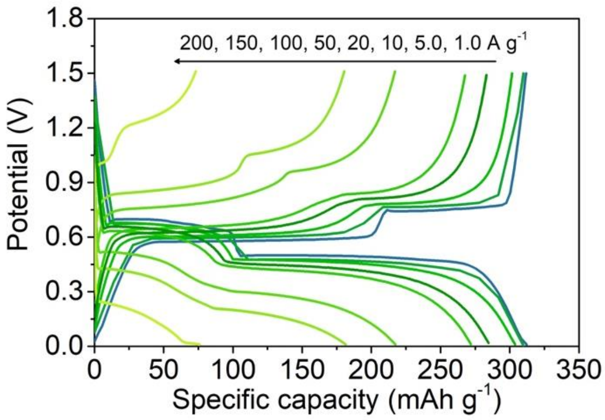

Fig. S18.
Charge-discharge curves of HBiC at different current densities.

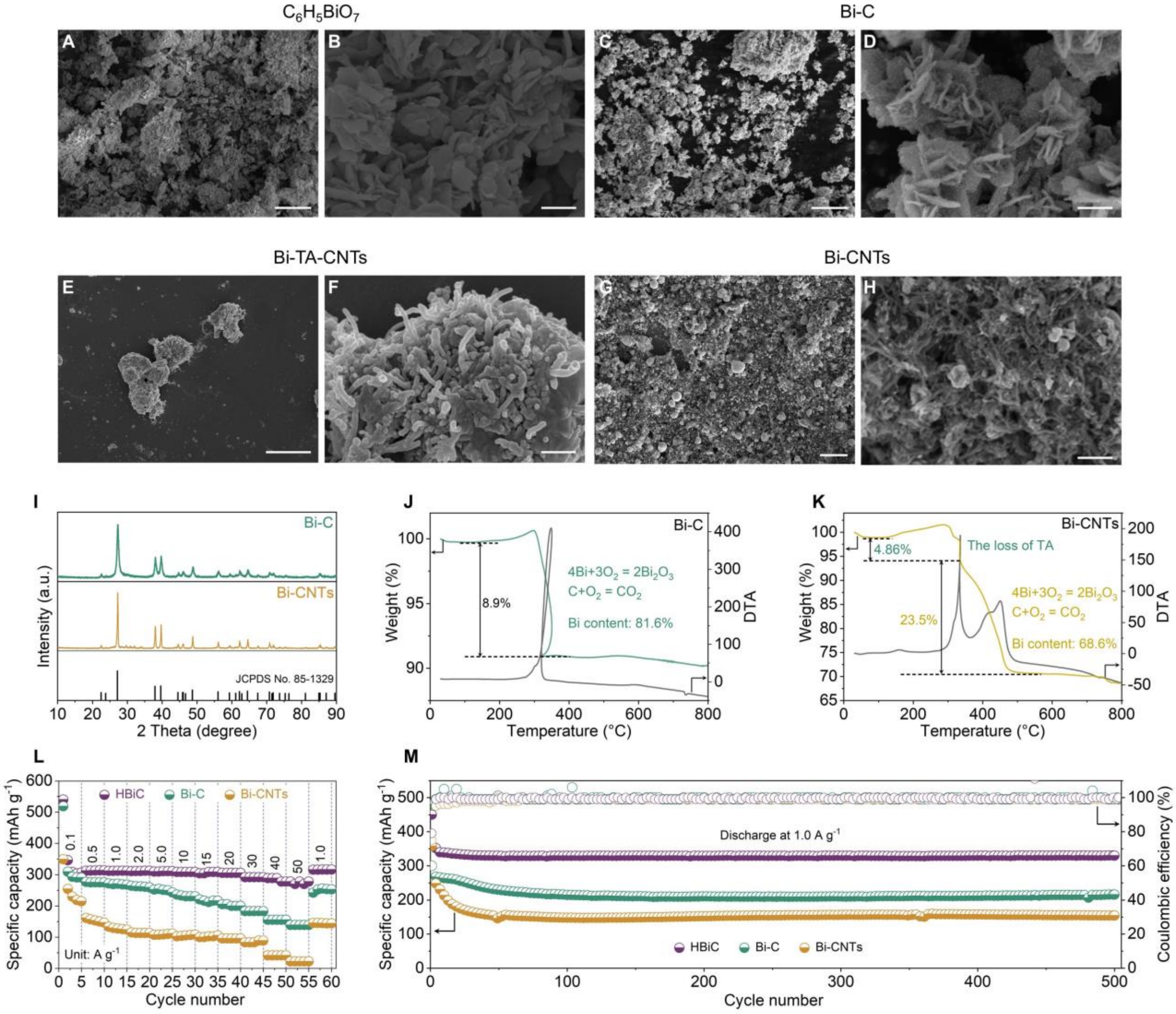

Fig. S19.

Morphology, phase, and sodium storage performance characterizations of different structured Bi-based materials. SEM images of (A and B) commercial bismuth citrate, (C and D) carbon sheets-based Bi (Bi-C) composite, (E and F) Bi-TA-CNTs, and (G and H) carbon nanotubes-based Bi (Bi-CNTs) composite. The disordered Bi-C and Bi-CNTs with simple ordered structure but without pores were prepared. (I) XRD patterns of Bi-C and Bi-CNTs. The sharp diffraction peaks in the XRD patterns suggested the existences of the hexagonal Bi phase both in Bi-C and Bi-CNTs. (J and K) TGA-DTA curves of Bi-C and Bi-CNTs. The Bi contents of Bi-C and Bi-CNTs were determined to be 81.6% and 68.6%, respectively. (L) Rate capabilities of HBiC, Bi-C, and Bi-CNTs at different current rates. The rate capabilities showed that the HBiC exhibited excellent capacities and stability at varied current density, while Bi-C and Bi-CNTs displayed low capacities at varied current density, even low to 138.9 and 22.2 mAh g^{-1} at a high current density of 50 A g^{-1}, respectively. (M) Cycling performances of HBiC, Bi-C, and Bi-CNTs at 1.0 A g^{-1}. Cycle performances of HBiC, Bi-C, and Bi-CNTs were obtained with capacities of 329.9, 215.7, and 154.1 mAh g^{-1} at a current density of 1.0 A g^{-1} after 500 cycles, respectively. These results indicated that the Bi-CNTs with simple ordered structure ensured fast electronic and ionic conductivity leading to a high initial capacity (357.7 mAh g^{-1}) similar to the theoretical capacity of Bi (385 mAh g^{-1}). However, due to the reduced order of Bi-CNTs, the

volume expansion of Bi nanoparticles during sodiation/desodiation processes could not be fully accommodated, thus resulting in a structure collapse and rapid decline of the cycle performance (reduced to 154.1 mAh g^{-1}) during the long-cycles (500 times). For Bi-C, although Bi-C had a higher Bi content (81.6%) than that of HBiC (67.1%), the Bi-C could not provide connectivity and fast electronic/ionic transport channels because of the lack of hierarchical structures, thus resulting in a dramatic decline in high-rate capacity and a decay of cycle performance. Scale bars are 5 μm (A, C, E, and G) and 500 nm (B, D, F, and H).

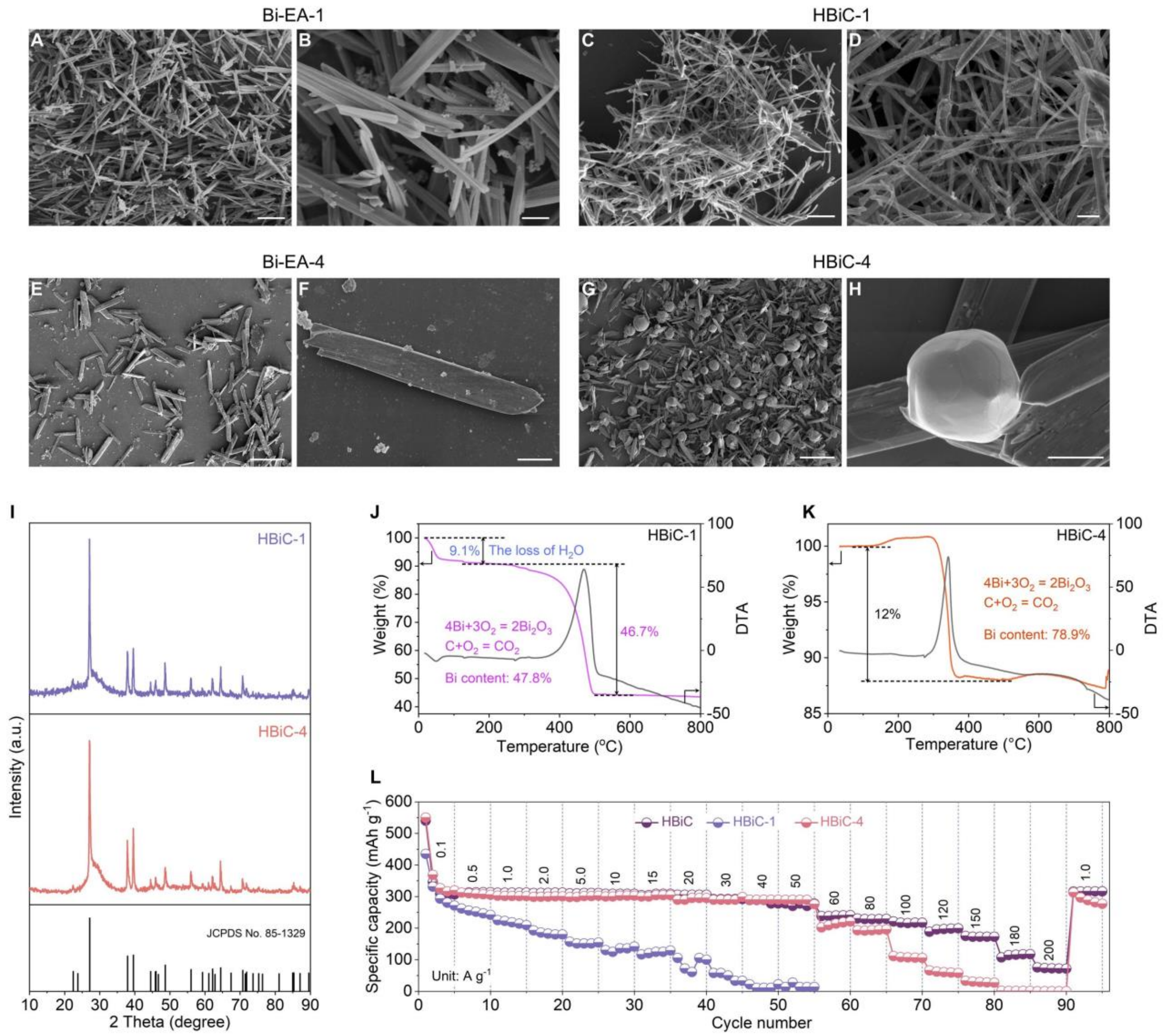

Fig. S20.

The effect of different Bi contents on morphology and electrochemical performances of HBiC. SEM images of Bi-EA mesocrystals with different molar ratios of Bi^{3+} ions to EA before and after carbonization. (A and B) Bi-EA-1, (C and D) HBiC-1, (E and F) Bi-EA-4, and (G and H) HBiC-4. The morphology of Bi-EA-1 mesocrystals and Bi-EA-4 mesocrystals were well inherited in the HBiC-1 and HBiC-4, similar to that of HBiC. (I) XRD patterns of HBiC-1 and HBiC-4. HBiC-1 and HBiC-4 also presented the characteristic peaks of the hexagonal Bi phase. (J and K) TGA-DTA curves of HBiC-1 and HBiC-4. The Bi contents of HBiC-1 and HBiC-4 were calculated to be 46.7% and 78.9%, respectively. (L) Rate capabilities of HBiC, HBiC-1, and HBiC-4 at different current densities. Compared with HBiC, the rate capabilities of HBiC-1 were inferior with increased current densities, and finally reached zero at the high current of 50 A g^{-1} because of the low Bi content. The initial capacity of HBiC-4 (550.2 mAh g^{-1}) was slightly higher than that of HBiC (540.1 mAh g^{-1}) at low current density of 0.1 A g^{-1}, owing to the high Bi content. However, when the current density was increased to 180 A g^{-1}, its capacity reduced to zero, which might be ascribed to the pulverization of the aggregated Bi particles (~10 − 20 μm) without the support of the hierarchically order structure. Scale bars are 5 μm (A, C, F, and H), 1 μm (B and D), and 50 μm (E and G).

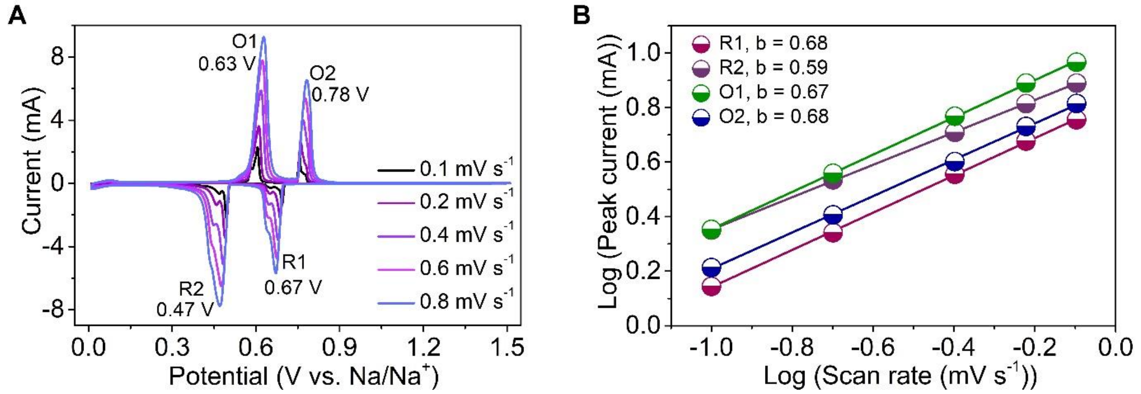

Fig. S21.

Kinetics process of Na storage mechanism of the HBiC electrode. (A) CV curves at different scan rates of 0.1, 0.2, 0.4, 0.6, and 0.8 mV s^{-1}. (B) Relationship between the peak currents and scan rates in logarithmic format. The redox peaks had similar shapes and peak potentials at increased scan rates, suggesting a small polarization voltage and fast reaction kinetics. The relationship between peak current (i, mA) and scan rate (v, mV s^{-1}) were analyzed by the power-law equation: $I = av^b$, where a and b were variables. As b values approached 0.5 or 1.0, a diffusion-controlled process (Faradaic) or a surface capacitance-dominated behavior was expected. When fitted two couples of redox peaks for the HBiC anode (R1/O1 and R2/O2), the b-values were 0.68/0.67 and 0.59/0.68 for R1/O1 and R2/O2, respectively, demonstrating that the redox process was Faradaic and depended on the diffusion of Na^+ ions.

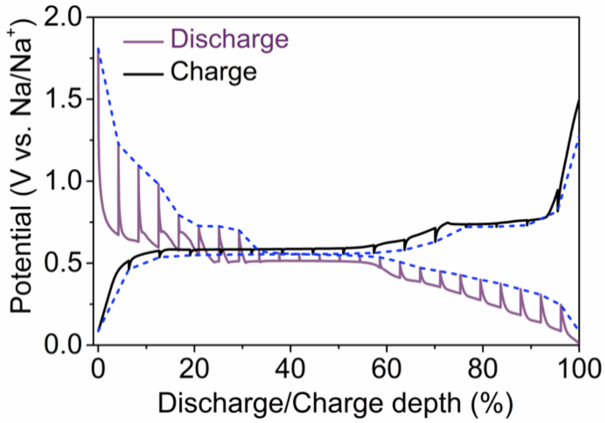

Fig. S22.
***Quasi*-equilibrium voltage curves of HBiC from galvanostatic intermittent titration technique (GITT) at 0.1 A g^{-1}.**

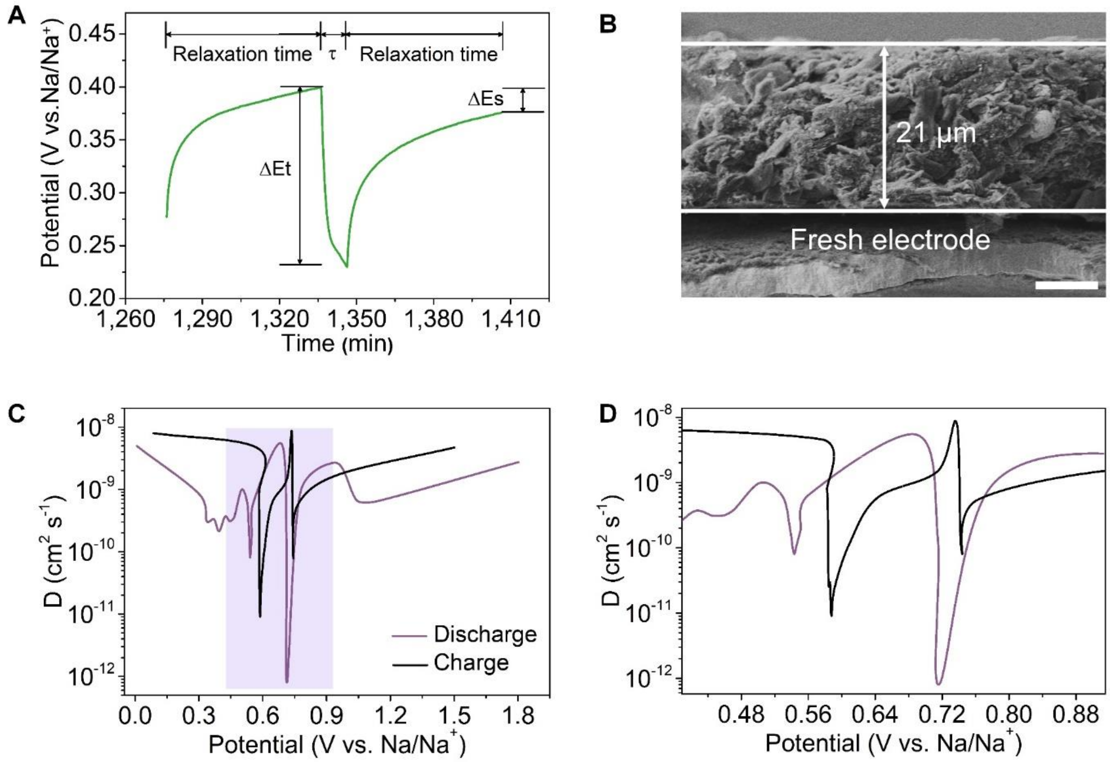

Fig. S23.

Sodium ions diffusion coefficient analysis of the HBiC electrode by GITT test. (A) A single titration process, which was composed of 10 min galvanostatic discharge-charge (pulse) at 0.1 A g^{-1} and followed by 60 min relaxation time. (B) Cross-sectional SEM image of HBiC electrode. (C and D) Na^+ diffusion coefficient vs. potential plot and corresponding enlarged profile of the selected area. The Na^+ diffusion coefficient was calculated as 1.36×10^{-9} cm^{-1} s^{-1} based on the following equation:

$$D = \frac{4L^2}{\pi\tau}\left(\frac{\Delta E_s}{\Delta E_t}\right)$$

Where t was the duration of the current pulse (s), τ was the relaxation time (s), ΔE_s was the steady-state potential change (V) by the current pulse, and ΔE_t was the potential change (V) during the constant current pulse. L was the sodium diffusion distance (cm), which was equal to the thickness of electrode. Scale bar is 5 μm (B).

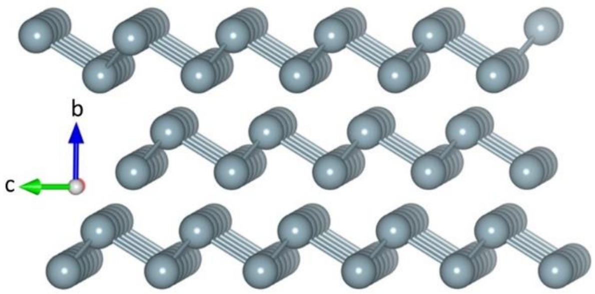

Fig. S24.
Structure model of the optimized (001) plane for Bi.

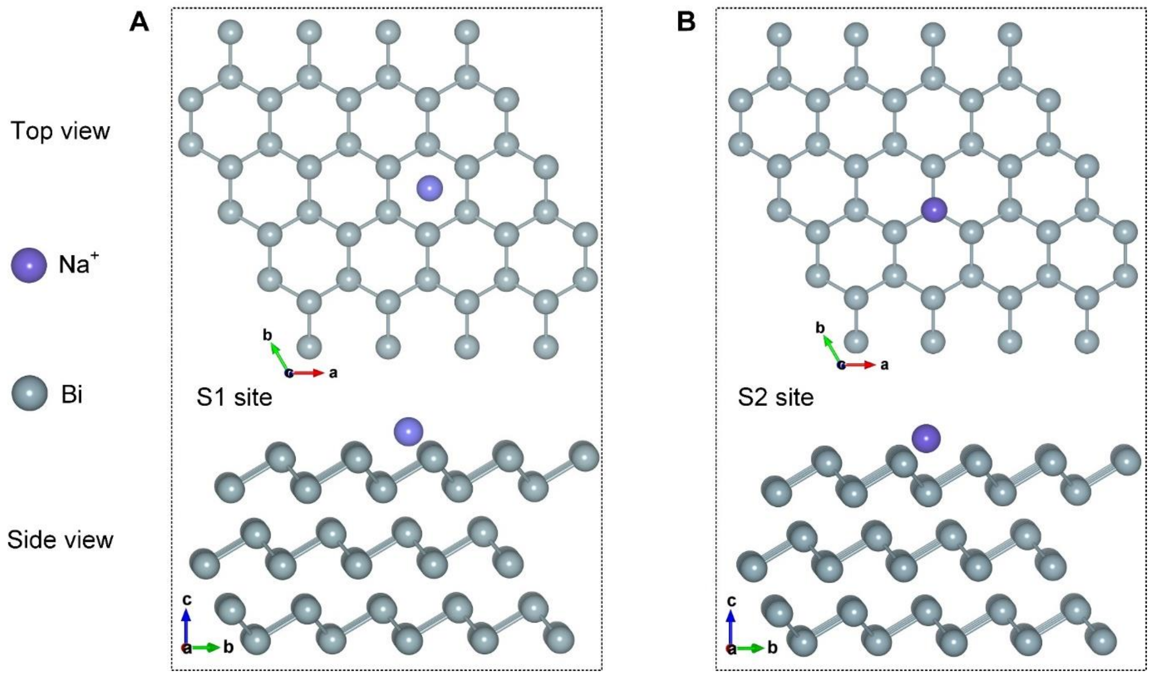

Fig. S25.
Top and side illustrations of simulations for a single Na^+ adsorbed on the Bi (001) plane. S1 site (above the center of the hexagonal ring) (A) and S2 site (on the top of Bi atom) (B).

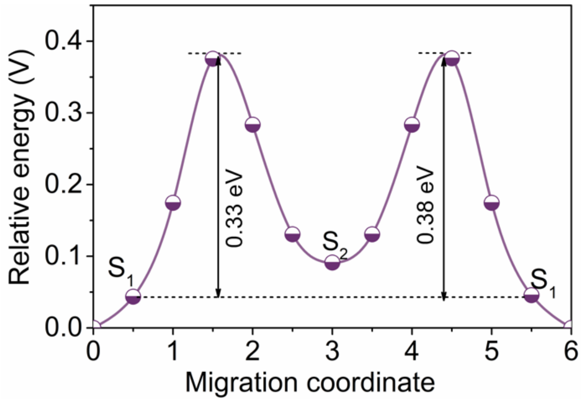

Fig. S26.
The curve of diffusion energy barriers of Na^{+} through the selected diffusion pathway between bismuth layers.

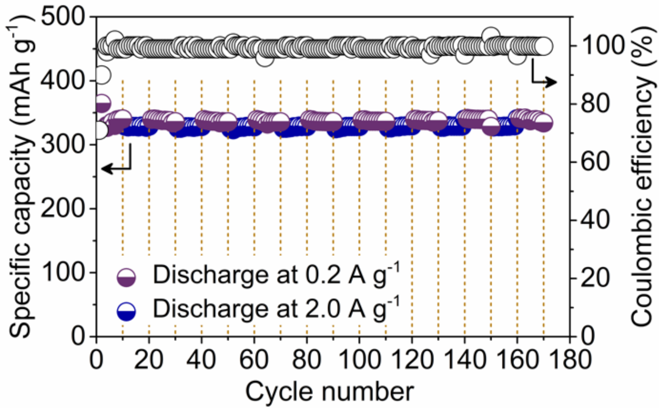

Fig. S27.
Specific capacities of HBiC under constant charge and discharge current densities of 2.0 A g^{-1} and 0.2 A g^{-1}, respectively.

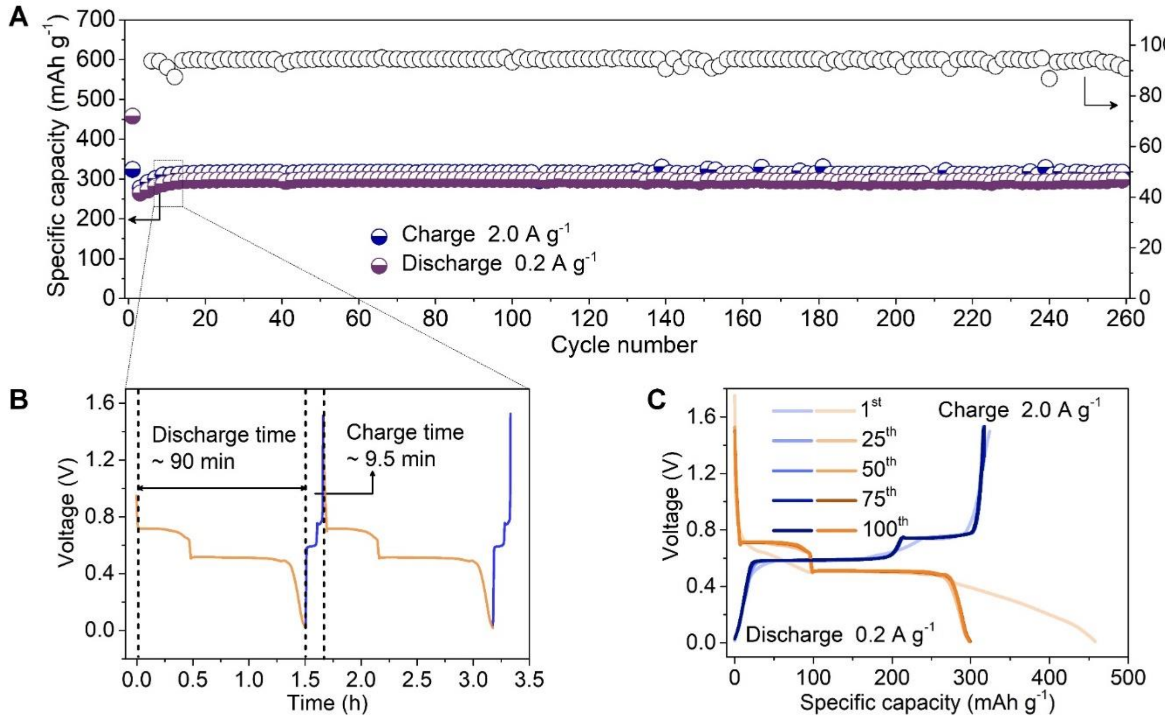

Fig. S28.

Electrode reversibility measurement under constant charge and discharge at current densities of 2.0 A g^{-1} and 0.2 A g^{-1}. (A) Cycling performance test. The corresponding capacities still maintained at 312 and 295 mAh g^{-1} after 250 cycles at 2.0 A g^{-1} and 0.2 A g^{-1}, respectively. (B) The 6th and 7th cycles voltage-time curves. These curves suggested one complete galvanostatic charging at 2.0 A g^{-1} took 9.5 min while the corresponding discharge duration at 0.2 A g^{-1} reached 90 min. (C) The selected charge-discharge voltage profiles in 100 cycles for HBiC electrode. The charge-discharge voltage profiles were well overlapped in 100 cycles, indicating the stable platform and good reversibility of HBiC hybrid.

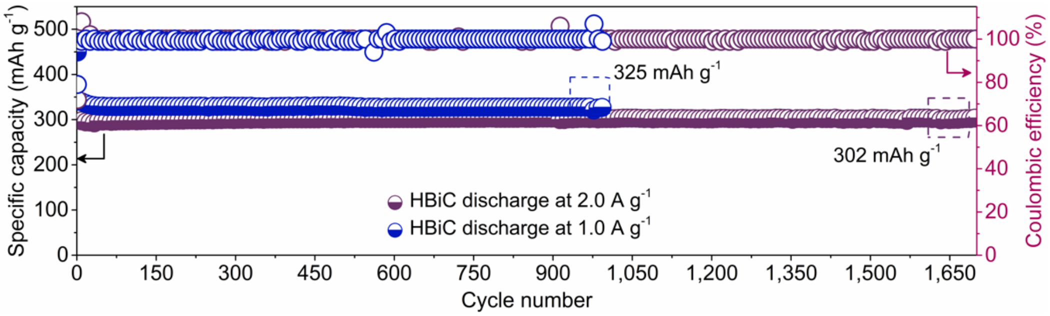

Fig. S29.
Cycling performance of HBiC at 1.0 and 2.0 A g^{-1}.

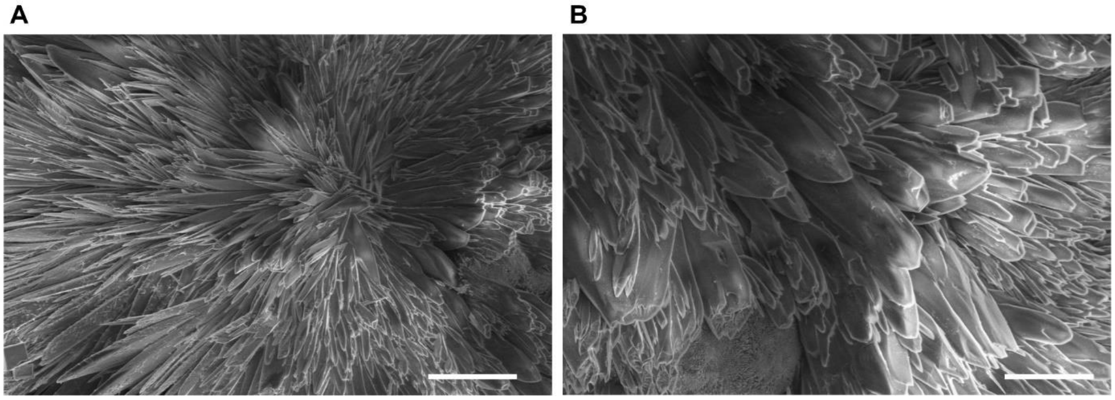

Fig. S30.
SEM images of HBiC electrode cycled at 2.0 A g^{-1} after 1,700 cycles. Scale bars are 20 μm (A) and 10 μm (B).

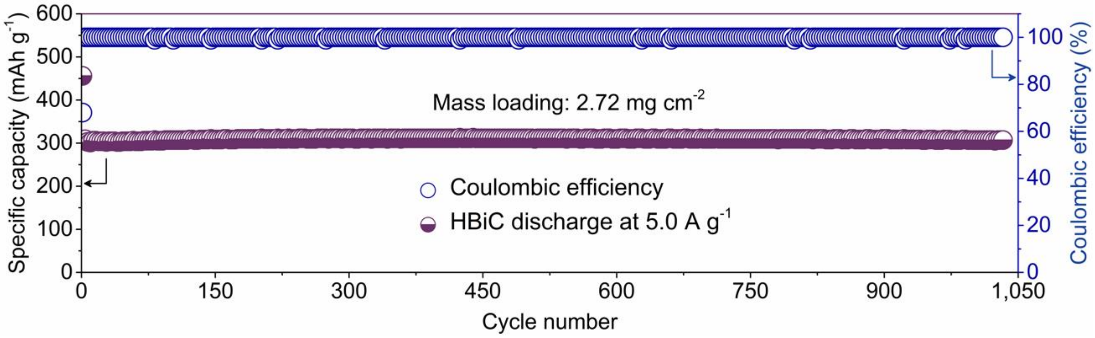

Fig. S31.

Cycling performance of HBiC with a mass loading of 2.72 mg cm^{-2} at 5.0 A g^{-1}.

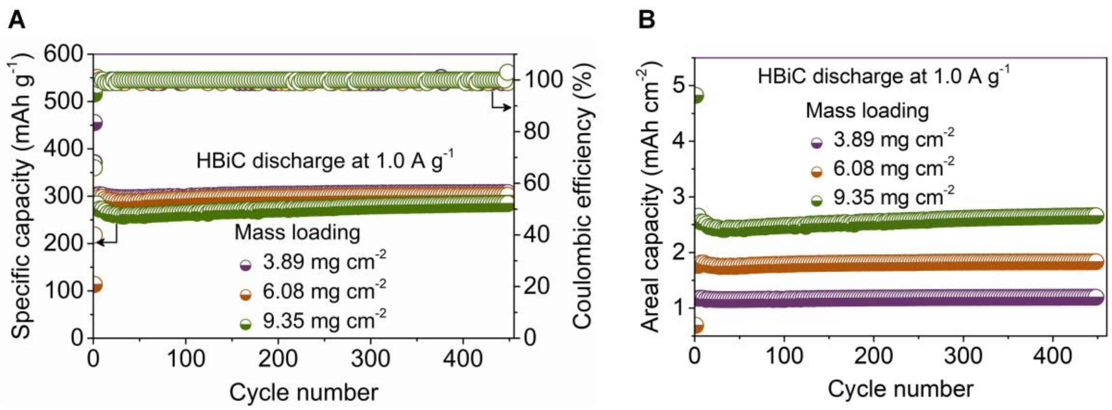

Fig. S32.

Electrochemical performances of HBiC at different mass loadings. (A) Cycling performance and (B) corresponding areal capacity with high mass loadings of 3.89, 6.08 and 9.35 mg cm^{-2}.

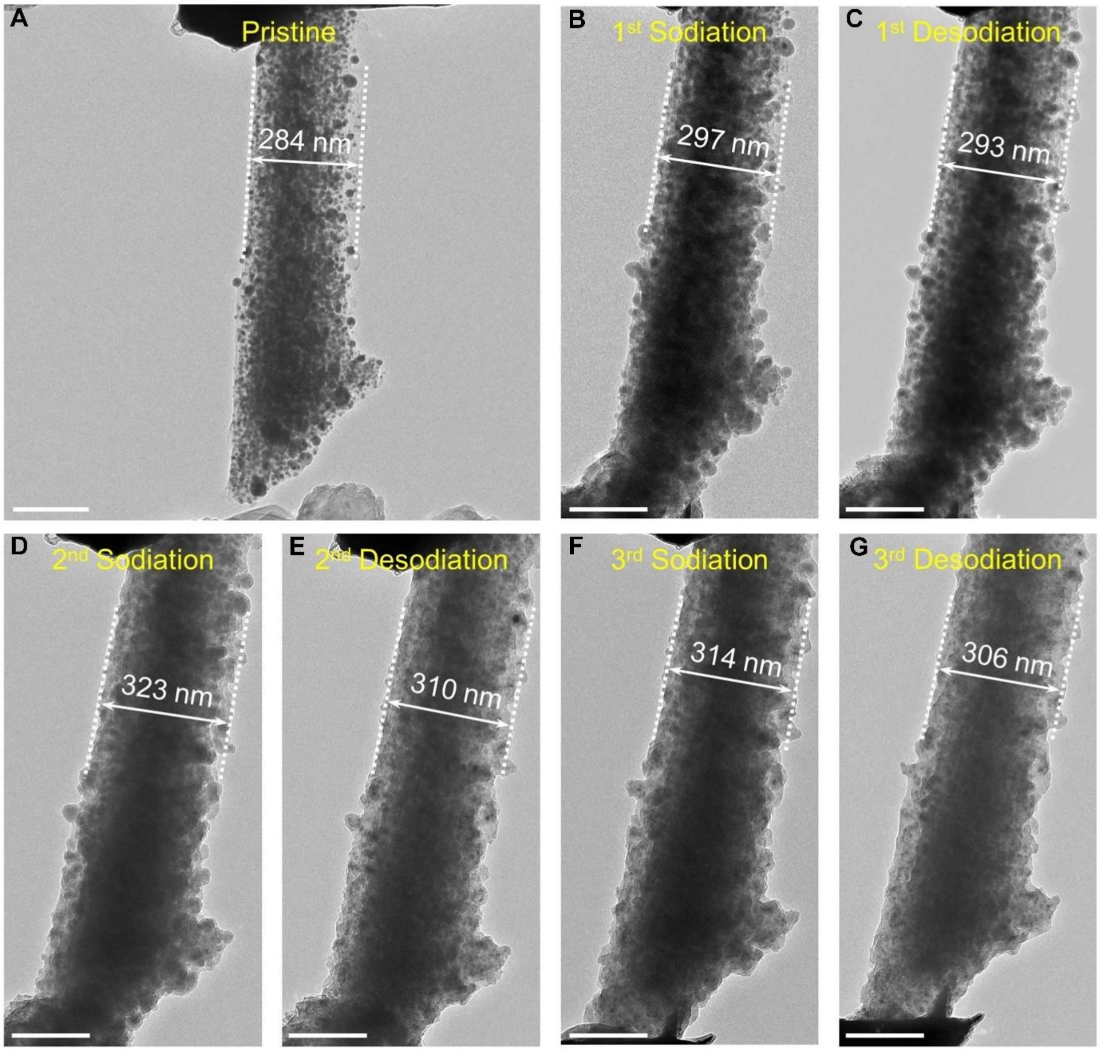

Fig. S33.

In-situ TEM images of HBiC at the pristine state and after different sodiation-desodiation cycles. The HBiC showed a gradual sodiation proceeded along the axial and radial direction, and the diameter of the HBiC slightly increased from 284 nm to 306 nm after three consequent sodiation-desodiation cycles. While the HBiC kept its structural integrity without mechanical degradation and cracking. Such robust structural integrity was greatly beneficial for the outstanding cycling performance. Scale bars are 200 nm (A to G).

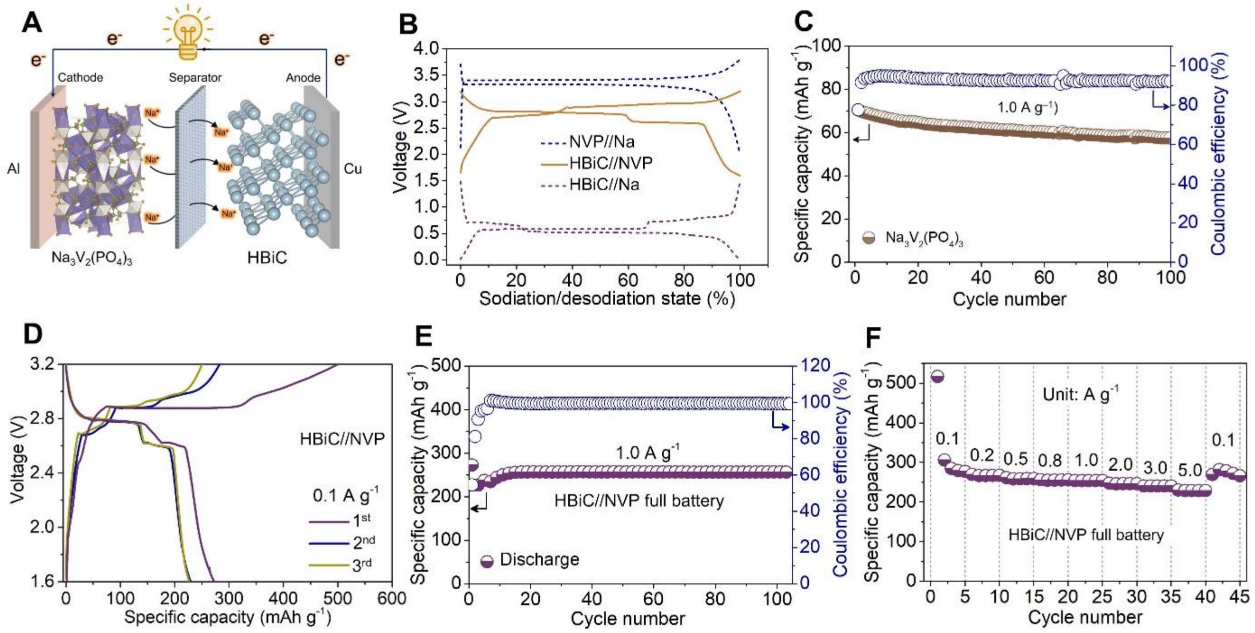

Fig. S34.

Electrochemical performances of full battery by pairing the HBiC anode with NVP cathode. (A) Working scheme. (B) Typical charge-discharge profiles of the NVP cathode, the HBiC anode and HBiC//NVP full cell. The HBiC//NVP full cell presented two voltage plateaus at 2.7 and 2.9 V in the charge cycle, corresponding to the voltage differences between NVP cathode (~3.4 V) and HBiC anode (0.7 and 0.5 V). (C) Cycling performance of NVP at 1.0 A g^{-1}. The NVP cathode existed a stable capacity of 60 mAh g^{-1} after 100 cycles. (D) Galvanostatic charge-discharge curves of HBiC//NVP full cell at 0.1 A g^{-1}. The overlapped charge-discharge profiles in selected cycles suggested a great electrochemical reversibility of HBiC//NVP full cell. (E and F) Cycling performance at 1.0 A g^{-1} and rate capability of HBiC//NVP full cell. The corresponding reversible capacity was maintained at 256 mAh g^{-1} after 100 cycles, with 100% capacity retention compared with the initial cycle.

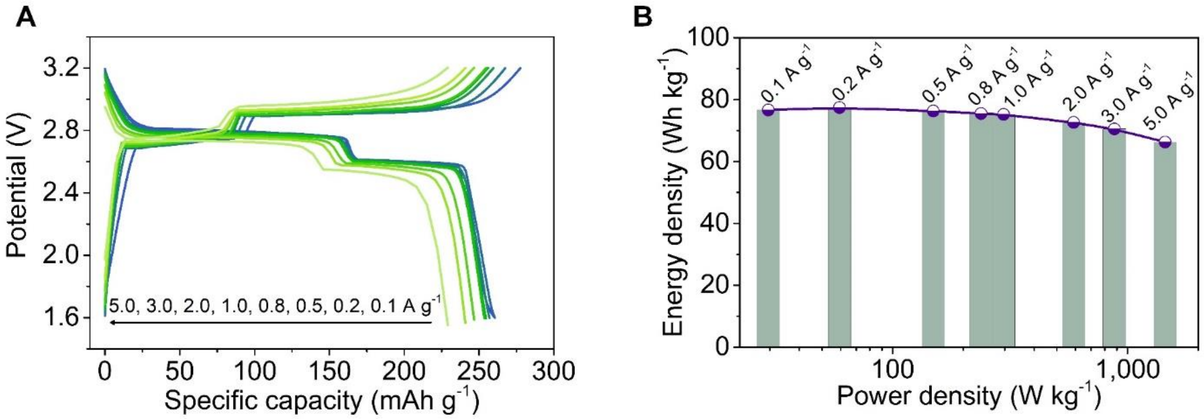

Fig. S35.

Electrochemical performance of HBiC//NVP full cell. (A) Charge-discharge curves at different rates. (B) Ragone plot (energy vs. power density), evaluated by the total mass.

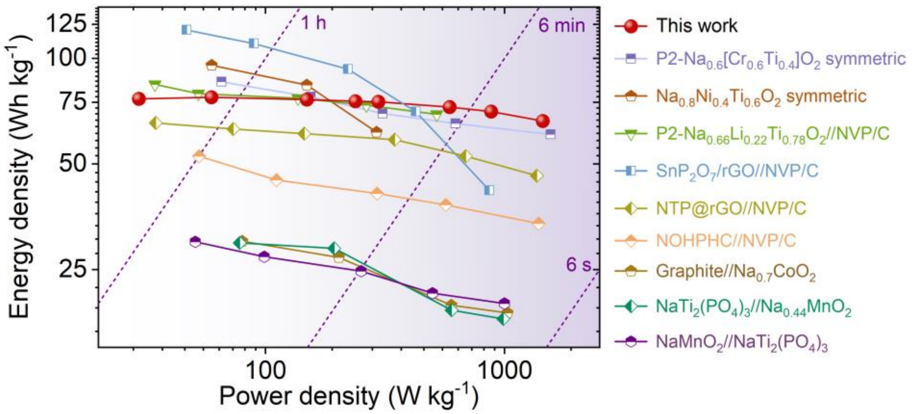

Fig. S36.

Ragone plot of the assembled HBiC//NVP full cell with comparisons to reported typical anode materials for SIBs: P2-$Na_{0.6}[Cr_{0.6}Ti_{0.4}]O_2$ symmetric (*48*), $Na_{0.8}Ni_{0.4}Ti_{0.6}O_2$ symmetric (*49*), P2-$Na_{0.66}Li_{0.22}Ti_{0.78}O_2$//NVP/C (*50*), SnP_2O_7/rGO//NVP/C (*51*), NTP@rGO//NVP/C (*52*), NOHPHC//NVP/C (*53*), Graphite//$Na_{0.7}CoO_2$ (*54*), $NaTi_2(PO_4)_3$//$Na_{0.44}MnO_2$ (*55*), $NaMnO_2$//$NaTi_2(PO_4)_3$ (*56*).

Table S1. ICP-OES results of Bi-EA mesocrystals before and after NaCl treatment.

Sample	Bi-EA mesocrystals before NaCl treatment	Bi-EA mesocrystals after NaCl treatment
Bi content (%)	46.3	47.3

Table S2. ICP-OES results of Bi-EA mesocrystals and HBiC.

Sample	Bi-EA mesocrystals	HBiC
Bi content (%)	46.3	67.1

Table S3. Comparison of the BET characteristic parameters determined by N_2 adsorption data of Bi-EA mesocrystals and HBiC.

	Bi-EA mesocrystals	HBiC
BET specific surface area	10.7 m^2 g^{-1}	91.6 m^2 g^{-1}
t-plot open surface area	10.7 m^2 g^{-1}	20.678 m^2 g^{-1}
Specific surface area contributed by micropores	0%	77.4%
Total pore volume	0.0322 cm^3 g^{-1}	0.0836 cm^3 g^{-1}
Micropore volume	0.002 cm^3 g^{-1}	0.0317 cm^3 g^{-1}
Micropore on total pore volume	6.2%	37.9%
Mesopore volume	0.0185 cm^3 g^{-1}	0.0391 cm^3 g^{-1}
Mesopore on total pore volume	57.4%	46.8%
Pore size	2.6 nm	3.6 nm

Table S4. Comparison of reported works on Bi-based electrodes for SIBs.

Electrode	Rate performance (mAh g^{-1}) at (Y) current density (mA g^{-1})	Cyclability (capacity retention (mAh g^{-1}) @ cycle number) at current density	First cycle Coulombic Efficiency	Reference
Bi@graphene	250 (1,280)	~110 @ 50 at 1,280 mA g^{-1}	55.5% @ 40mA g^{-1}	(*57*)
Bismuth nanorod bundle	102.3 (2,000)	302 @ 150 at 50 mA g^{-1}	55% @ 50 mA g^{-1}	(*58*)
Bi@C microsphere	83.4 (2,000)	123.5 @ 100 at 100 mA g^{-1}	45% @ 100 mA g^{-1}	(*59*)
Bi/CNF	85.6 (1,000)	186 @ 100 at 50 mA g^{-1}	53% @ 50 mA g^{-1}	(*60*)
Bi/CFC	120 (2,000)	350 @ 300 at 50 mA g^{-1}	61.2% @ 50 mA g^{-1}	(*61*)
Bi-NS@C	~110 (2,000)	106 @ 1,000 at 200 mA g^{-1}	——	(*62*)
Bulk Bi	356 (2,000)	389 @ 2,000 at 400 mA g^{-1}	94.8% @ 400 mA g^{-1}	(*35*)
Bi/Ni	206.4 (2,000)	302 @ 100 at 200 mA g^{-1}	——	(*63*)
Bi/C nanofibers	69.04 (3,200)	273.2 @ 500 at 100 mA g^{-1}	55.8% @ 100 mA g^{-1}	(*64*)
Bi@Graphite	113 (48,000)	~140 @ 10,000 at 3,200 mA g^{-1}	74.5% @ 80 mA g^{-1}	(*36*)
Bi@3DGFs	180 (50,000)	185.2 @ 2,000 at 10,000 mA g^{-1}	36% @ 100 mA g^{-1}	(*65*)
Bi@C	232 (60,000)	265 @ 30,000 at 8,000 mA g^{-1}	50.3% @ 800 mA g^{-1}	(*27*)
Bi@N-C	368 (2,000)	302 @ 1,000 at 1,000 mA g^{-1}	85.7% @ 50 mA g^{-1}	(*34*)
Bi@Void@C-2	173 (100,000)	198 @ 10,000 at 20,000 mA g^{-1}	46% @ 1,000 mA g^{-1}	(*66*)

Bi@N-C	178 (100,000)	235 @ 2,000 at 10,000 mA g^{-1}	36.5% @ 1,000 mA g^{-1}	(*33*)
Bi–C/CF	110 (2,400)	~340 @ 500 at 500 mA g^{-1}	——	(*67*)
FLB-G	263.2 (1,176)	317 @ 1,000 at 706 mA g^{-1}	——	(*68*)
**HBiC**	**72.5 (200,000)**	**263 @ 15,000 at 5,000 mA g^{-1}**	**79.9% @ 1,000 mA g^{-1}**	**This work**

Table S5. Lattice parameters and calculated surface energies of Bi with different orientations.

Surface	Lattice parameters (Å)	Surface energy ($J\ m^{-2}$)
(001)	a = 9.92, b = 9.92	0.18
(101)	a = 14.94, b = 9.92	0.26
(110)	a = 11.86, b = 15.74	0.39
(111)	a = 14.94, b = 14.94	0.35
(211)	a=19.71, b = 14.94	0.31
(221)	a = 15.74, b = 25.41	0.34
(201)	a = 25.40, b = 9.09	0.40
(210)	a = 11.86, b = 24.03	0.33
(212)	a = 14.97, b = 21.72	0.49
(102)	a = 19.71, b = 9.09	0.57

Table S6. Adsorption energy of Na^+ on the two stable adsorption sites of the Bi (001) crystal plane.

Site	Distance d (Å)	Adsorption energy (eV)
S1	2.29	−0.67
S2	2.38	−0.45

Captions for Supplementary Movies

Movie S1.
Time-resolved TEM observation of HBiC during the initial sodiation-desodiation process.

Movie S2.
In-situ TEM observation of HBiC during three sodiation-desodiation cycles.

REFERENCES AND NOTES

1. C. Yuan, W. Ji, R. Xing, J. Li, E. Gazit, X. Yan, Hierarchically oriented organization in supramolecular peptide crystals. *Nat. Rev. Chem.* **3**, 567−588 (2019).

2. M. R. Jones, N. C. Seeman, C. A. Mirkin, Programmable materials and the nature of the DNA bond. *Science* **347**, 1260901 (2015).

3. Y. Lu, J. Lin, L. Wang, L. Zhang, C. Cai, Self-assembly of copolymer micelles: Higher-level assembly for constructing hierarchical structure. *Chem. Rev.* **120**, 4111−4140 (2020).

4. Y. Sun, C. Chen, P. J. Stang, Soft materials with diverse suprastructures via the self-assembly of metal-organic complexes. *Acc. Chem. Res.* **52**, 802−817 (2019).

5. T. Christoff-Tempesta, Y. Cho, D. Y. Kim, M. Geri, G. Lamour, A. J. Lew, X. Zuo, W. R. Lindemann, J. H. Ortony, Self-assembly of aramid amphiphiles into ultra-stable nanoribbons and aligned nanoribbon threads. *Nat. Nanotechnol.* **16**, 447−454 (2021).

6. D. J. Park, C. Zhang, J. C. Ku, Y. Zhou, G. C. Schatz, C. A. Mirkin, Plasmonic photonic crystals realized through DNA-programmable assembly. *Proc. Natl. Acad. Sci. U.S.A.* **112**, 977−981 (2015).

7. Y. F. Chao, R. Jalili, Y. Ge, C. Y. Wang, T. Zheng, K. W. Shu, G. G. Wallace, Self-assembly of flexible free-standing 3D porous MoS_2-reduced graphene oxide structure for high-performance lithium-ion batteries. *Adv. Funct. Mater.* **27**, 1700234 (2017).

8. Z. Fan, L. Sun, Y. Huang, Y. Wang, M. Zhang, Bioinspired fluorescent dipeptide nanoparticles for targeted cancer cell imaging and real-time monitoring of drug release. *Nat. Nanotechnol.* **11**, 388−394 (2016).

9. K. J. Chen, D. G. Madden, S. Mukherjee, T. Pham, K. A. Forrest, A. Kumar, B. Space, J. Kong, Q. Y. Zhang, M. J. Zaworotko, Synergistic sorbent separation for one-step ethylene purification from a four-component mixture. *Science* **366**, 241−246 (2019).

10. M. Mon, R. Bruno, S. Sanz-Navarro, C. Negro, J. Ferrando-Soria, L. Bartella, L. Di Donna, M. Prejano, T. Marino, A. Leyva-Perez, D. Armentano, E. Pardo, Hydrolase-like catalysis and structural resolution of natural products by a metal-organic framework. *Nat. Commun.* **11**, 3080 (2020).

11. R. Freeman, M. Han, Z. Alvarez, J. A. Lewis, J. R. Wester, N. Stephanopoulos, M. T. Mcclendon, C. Lynsky, J. M. Godbe, H. Sangji, E. Luijten, S. I. Stupp, Reversible self-assembly of superstructured networks. *Science* **362**, 808−813 (2018).

12. Y. Bai, Q. Luo, J. Liu, Protein self-assembly via supramolecular strategies. *Chem. Soc. Rev.* **45**, 2756−2767 (2016).

13. A. F. Mason, B. C. Buddingh, D. S. Williams, J. C. M. Van Hest, Hierarchical self-assembly of a copolymer-stabilized coacervate protocell. *J. Am. Chem. Soc.* **139**, 17309−17312 (2017).

14. X. Liu, F. Zhang, X. Jing, M. Pan, P. Liu, W. Li, B. Zhu, J. Li, H. Chen, L. Wang, J. Lin, Y. Liu, D. Zhao, H. Yan, C. Fan, Complex silica composite nanomaterials templated with DNA origami. *Nature* **559**, 593−598 (2018).

15. O. Vonshak, Y. Divon, S. Forste, D. Garenne, V. Noireaux, R. Lipowsky, S. Rudorf, S. S. Daube, R. H. Bar-Ziv, Programming multi-protein assembly by gene-brush patterns and two-dimensional compartment geometry. *Nat. Nanotechnol.* **15**, 783−791 (2020).

16. R. Chakrabarty, P. S. Mukherjee, P. J. Stang, Supramolecular coordination: Self-assembly of finite two- and three-dimensional ensembles. *Chem. Rev.* **111**, 6810−6918 (2011).

17. D. Kashiwagi, H. K. Shen, S. Sim, K. Sano, Y. Ishida, A. Kimura, T. Niwa, H. Taguchi, T. Aida, Molecularly engineered “janus groel”: Application to supramolecular copolymerization with a higher level of sequence control. *J. Am. Chem. Soc.* **142**, 13310−13315 (2020).

18. C. Li, Q. Li, Y. V. Kaneti, D. Hou, Y. Yamauchi, Y. Mai, Self-assembly of block copolymers towards mesoporous materials for energy storage and conversion systems. *Chem. Soc. Rev.* **49**, 4681−4736 (2020).

19. H. Qiu, Z. M. Hudson, M. A. Winnik, I. Manners, Multidimensional hierarchical self-assembly of amphiphilic cylindrical block comicelles. *Science* **347**, 1329−1332 (2015).

20. H. Ejima, J. J. Richardson, K. Liang, J. P. Best, M. P. Van Koeverden, G. K. Such, J. Cui, F. Caruso, One-step assembly of coordination complexes for versatile film and particle engineering. *Science* **341**, 154−157 (2013).

21. J. Guo, B. L. Tardy, A. J. Christofferson, Y. Dai, J. J. Richardson, W. Zhu, M. Hu, Y. Ju, J. Cui, R. R. Dagastine, I. Yarovsky, F. Caruso, Modular assembly of superstructures from polyphenol-functionalized building blocks. *Nat. Nanotechnol.* **11**, 1105−1111 (2016).

22. J. Guo, M. Suastegui, K. K. Sakimoto, V. M. Moody, G. Xiao, D. G. Nocera, N. S. Joshi, Light-driven fine chemical production in yeast biohybrids. *Science* **362**, 813−816 (2018).

23. H. Lee, S. M. Dellatore, W. M. Miller, P. B. Messersmith, Mussel-inspired surface chemistry for multifunctional coatings. *Science* **318**, 426−430 (2007).

24. S. J. Yang, M. Antonietti, N. Fechler, Self-assembly of metal phenolic mesocrystals and morphosynthetic transformation toward hierarchically porous carbons. *J. Am. Chem. Soc.* **137**, 8269−8273 (2015).

25. Z. Bao, J. Wang, Z. Zhang, H. Xing, Q. Yang, Y. Yang, H. Wu, R. Krishna, W. Zhou, B. Chen, Q. Ren, Molecular sieving of ethane from ethylene through the molecular cross-section size differentiation in gallate-based metal-organic frameworks. *Angew. Chem. Int. Ed.* **57**, 16020−16025 (2018).

26. Z. Lin, J. Zhou, C. Cortez-Jugo, Y. Han, Y. Ma, S. Pan, E. Hanssen, J. J. Richardson, F. Caruso, Ordered mesoporous metal-phenolic network particles. *J. Am. Chem. Soc.* **142**, 335−341 (2020).

27. P. Xiong, P. Bai, A. Li, B. Li, M. Cheng, Y. Chen, S. Huang, Q. Iang, X.-H. Bu, Y. Xu, Bismuth nanoparticle@carbon composite anodes for ultralong cycle life and high-rate sodium-ion batteries. *Adv. Mater.* **31**, 1904771 (2019).

28. W. Xu, H. Wang, Y. Tao, X. Zheng, The structural organization of *N*-methyl-2-pyrrolidinone in binary mixtures probed by Raman spectroscopy: Experimental and quantum chemical results. *J. Raman Spectrosc.* **49**, 362−371 (2018).

29. S. J. Lee, B. S. Cheong, H. G. Cho, Surface-enhanced Raman spectroscopic studies of ellagic acid in silver colloids. *Bull. Kor. Chem. Soc.* **36**, 1637−1644 (2015).

30. M. Rossi, J. Erlebacher, D. E. Zacharias, H. L. Carrell, B. Iannucci, The crystal and molecular structure of ellagic acid dihydrate: A dietary anti-cancer agent. *Carcinogenesis* **12**, 2227−2232 (1991).

31. Z. Liu, W. He, Z. Guo, Metal coordination in photoluminescent sensing. *Chem. Soc. Rev.* **42**, 1568−1600 (2013).

32. C. F. Wang, R. F. Li, X. Y. Chen, R. J. Wei, L. S. Zheng, J. Tao, Synergetic spin crossover and fluorescence in one-dimensional hybrid complexes. *Angew. Chem. Int. Ed.* **54**, 1574−1577 (2015).

33. H. Yang, R. Xu, Y. Yao, S. Ye, X. Zhou, Y. Yu, Multicore-shell Bi@N-doped carbon nanospheres for high power density and long cycle life sodium- and potassium-ion anodes. *Adv. Funct. Mater.* **29**, 1809195 (2019).

34. P. Xue, N. Wang, Z. Fang, Z. Lu, X. Xu, L. Wang, Y. Du, X. Ren, Z. Bai, S. Dou, G. Yu, Rayleigh-instability-induced bismuth nanorod@nitrogen-doped carbon nanotubes as a long cycling and high rate anode for sodium-ion batteries. *Nano Lett.* **19**, 1998−2004 (2019).

35. C. Wang, L. Wang, F. Li, F. Cheng, J. Chen, Bulk bismuth as a high-capacity and ultralong cycle-life anode for sodium-ion batteries by coupling with glyme-based electrolytes. *Adv. Mater.* **29**, 1702212 (2017).

36. J. Chen, X. Fan, X. Ji, T. Gao, S. Hou, X. Zhou, L. N. Wang, F. Wang, C. Yang, L. Chen, C. Wang, Intercalation of Bi nanoparticles into graphite results in an ultra-fast and ultra-stable anode material for sodium-ion batteries. *Energy Environ. Sci.* **11**, 1218−1225 (2018).

37. H. Ying, W.-Q. Han, Metallic Sn-based anode materials: Application in high-performance lithium-ion and sodium-ion batteries. *Adv. Sci.* **4**, 1700298 (2017).

38. W. Li, S. Hu, X. Luo, Z. Li, X. Sun, M. Li, F. Liu, Y. Yu, Confined amorphous red phosphorus in MOF-derived N-doped microporous carbon as a superior anode for sodium-ion battery. *Adv. Mater.* **29**, 1605820 (2017).

39. W. Luo, F. Li, J.-J. Gaumet, P. Magri, S. Diliberto, L. Zhou, L. Mai, Bottom-up confined synthesis of nanorod-in-nanotube structured Sb@N-C for durable lithium and sodium storage. *Adv. Energy Mater.* **8**, 1703237 (2018).

40. J. Zhou, L. Wang, M. Yang, J. Wu, F. Chen, W. Huang, N. Han, H. Ye, F. Zhao, Y. Li, Y. Li, Hierarchical VS_2 nanosheet assemblies: A universal host material for the reversible storage of alkali metal ions. *Adv. Mater.* **29**, 1702061 (2017).

41. N. Nitta, G. Yushin, High-capacity anode materials for lithium-ion batteries: Choice of elements and structures for active particles. *Part. Part. Syst. Charact.* **31**, 317−336 (2014).

42. M. J. Frisch, G. W. Trucks, H. B. Schlegel, G. E. Scuseria, M. A. Robb, J. R. Cheeseman, G. Scalmani, V. Barone, B. Mennucci, G. A. Petersson, H. Nakatsuji, M. Caricato, X. Li, H. P. Hratchian, A. F. Izmaylov, J. Bloino, G. Zheng, J. L. Sonnenberg, M. Hada, M. Ehara, K. Toyota, R. Fukuda, J. Hasegawa, M. Ishida, T. Nakajima, Y. Honda, O. Kitao, H. Nakai, T. Vreven, J. A. Montgomery, J. J. E. Peralta, F. Ogliaro, M. Bearpark, J. J. Heyd, E. Brothers, K. N. Kudin, V. N. Taroverov, T. Keith, R. Kobayashi, J. Normand, K. Raghavachari, A. Rendell, J. C. Burant, S. S. Iyengar, J. Tomasi, M. Cossi, N. Rega, J. M. Millam, M. Klene, J. E. Knox, J. B. Cross, V. Bakken, C. Adamo, J. Jaramillo, R. Gomperts, R. E. Stratmann, O. Yazyev, A. J. Austin, R. Cammi, C. Pomelli, J. W. Ochterski, R. L. Martin, K. Morokuma, V. G. Zakrzewski, G. A. Voth, P. Salvador, J. J. Dannenberg, S. Dapprich, A. D. Daniels, O. Farkas, J. B. Foresman, J. V. Ortiz, J. Cioslowski, D. J. Fox, Gaussian 09 (revision d.01) v. Revision D.01, (Gaussian Inc., 2013).

43. Y. Zhao, D. G. Truhlar, Density functionals with broad applicability in chemistry. *Acc. Chem. Res.* **41**, 157−167 (2008).

44. R. Krishnan, J. S. Binkley, R. Seeger, J. A. Pople, Self-consistent molecular-orbital methods. 20. Basis set for correlated wave-functions. *J. Chem. Phys.* **72**, 650−654 (1980).

45. P. J. Hay, W. R. Wadt, *Ab* initio effective core potentials for molecular calculations. Potentials for the transition metal atoms Sc to Hg. *J. Chem. Phys.* **82**, 270−283 (1985).

46. S. Grimme, J. Antony, S. Ehrlich, H. Krieg, A consistent and accurate *ab initio* parametrization of density functional dispersion correction (DFT-D) for the 94 elements H-Pu. *J. Chem. Phys.* **132**, 154104 (2010).

47. A. V. Marenich, C. J. Cramer, D. G. Truhlar, Universal solvation model based on solute electron density and on a continuum model of the solvent defined by the bulk dielectric constant and atomic surface tensions. *J. Phys. Chem. B* **113**, 6378–6396 (2009).

48. Y. Wang, R. Xiao, Y. S. Hu, M. Avdeev, L. Chen, P2-$Na_{0.6}[Cr_{0.6}Ti_{0.4}]O_2$ cation-disordered electrode for high-rate symmetric rechargeable sodium-ion batteries. *Nat. Commun.* **6**, 6954 (2015).

49. S. Guo, H. Yu, P. Liu, Y. Ren, T. Zhang, M. Chen, M. Ishida, H. Zhou, High-performance symmetric sodium-ion batteries using a new, bipolar O3-type material, $Na_{0.8}Ni_{0.4}Ti_{0.6}O_2$. *Energy Environ. Sci.* **8**, 1237–1244 (2015).

50. Y. Wang, X. Yu, S. Xu, J. Bai, R. Xiao, Y.-S. Hu, H. Li, X.-Q. Yang, L. Chen, X. Huang, A zero-strain layered metal oxide as the negative electrode for long-life sodium-ion batteries. *Nat. Commun.* **4**, 2365 (2013).

51. J. Pan, S. Chen, D. Zhang, X. Xu, Y. Sun, F. Tian, P. Gao, J. Yang, SnP_2O_7 covered carbon nanosheets as a long-life and high-rate anode material for sodium-ion batteries. *Adv. Funct. Mater.* **28**, 1804672 (2018).

52. Y. Fang, L. Xiao, J. Qian, Y. Cao, X. Ai, Y. Huang, H. Yang, 3D graphene decorated $NaTi_2(PO_4)_3$ microspheres as a superior high-rate and ultracycle-stable anode material for sodium ion batteries. *Adv. Energy Mater.* **6**, 1502197 (2016).

53. M. Huang, B. Xi, Z. Feng, J. Liu, J. Feng, Y. Qian, S. Xiong, Facile synthesis of N,O-codoped hard carbon on the kilogram scale for fast capacitive sodium storage. *J. Mater. Chem. A* **6**, 16465–16474 (2018).

54. I. Hasa, X. Dou, D. Buchholz, Y. Shao-Horn, J. Hassoun, S. Passerini, B. Scrosati, A sodium-ion battery exploiting layered oxide cathode, graphite anode and glyme-based electrolyte. *J. Power Sources* **310**, 26–31 (2016).

55. Z. Li, D. Young, K. Xiang, W. C. Carter, Y.-M. Chiang, Towards high power high energy aqueous sodium-ion batteries: The $NaTi_2(PO_4)_3/Na_{0.44}MnO_2$ System. *Adv. Energy Mater.* **3**, 290–294 (2013).

56. Z. Hou, X. Li, J. Liang, Y. Zhu, Y. Qian, An aqueous rechargeable sodium ion battery based on a $NaMnO_2$–$NaTi_2(PO_4)_3$ hybrid system for stationary energy storage. *J. Mater. Chem. A* **3**, 1400–1404 (2015).

57. D. Su, S. Dou, G. Wang, Bismuth: A new anode for the Na-ion battery. *Nano Energy* **12**, 88–95 (2015).

58. S. Liu, J. Feng, X. Bian, J. Liu, H. Xu, Advanced arrayed bismuth nanorod bundle anode for sodium-ion batteries. *J. Mater. Chem. A* **4**, 10098–10104 (2016).

59. F. Yang, F. Yu, Z. Zhang, K. Zhang, Y. Lai, J. Li, Bismuth nanoparticles embedded in carbon spheres as anode materials for sodium/lithium-ion batteries. *Chem. Eur. J.* **22**, 2333–2338 (2016).

60. Y. Jin, H. Yuan, J.-L. Lan, Y. Yu, Y.-H. Lin, X. Yang, Bio-inspired spider-web-like membranes with a hierarchical structure for high performance lithium/sodium ion battery electrodes: The case of 3D freestanding and binder-free bismuth/CNF anodes. *Nanoscale* **9**, 13298–13304 (2017).

61. S. Liu, Z. Luo, J. Guo, A. Pan, Z. Cai, S. Liang, Bismuth nanosheets grown on carbon fiber cloth as advanced binder-free anode for sodium-ion batteries. *Electrochem. Commun.* **81**, 10–13 (2017).

62. J. Qiu, S. Li, X. Su, Y. Wang, L. Xu, S. Yuan, H. Li, S. Zhang, Bismuth nano-spheres encapsulated in porous carbon network for robust and fast sodium storage. *Chem. Eng. J.* **320**, 300–307 (2017).

63. L. Wang, C. Wang, F. Li, F. Cheng, J. Chen, In situ synthesis of Bi nanoflakes on Ni foam for sodium-ion batteries. *Chem. Commun.* **54**, 38–41 (2017).

64. H. Yin, Q. Li, M. Cao, W. Zhang, H. Zhao, C. Li, K. Huo, M. Zhu, Nanosized-bismuth-embedded 1D carbon nanofibers as high-performance anodes for lithium-ion and sodium-ion batteries. *Nano Res.* **10**, 2156–2167 (2017).

65. X. L. Cheng, D. J. Li, Y. Wu, R. Xu, Y. Yu, Bismuth nanospheres embedded in three-dimensional (3D) porous graphene frameworks as high performance anodes for sodium- and potassium-ion batteries. *J. Mater. Chem. A* **7**, 4913–4921 (2019).

66. H. Yang, L. W. Chen, F. He, J. Zhang, Y. Feng, L. Zhao, B. Wang, L. He, Q. Zhang, Y. Yu, Optimizing the void size of yolk-shell Bi@Void@C nanospheres for high-power-density sodium-ion batteries. *Nano Lett.* **20**, 758–767 (2020).

67. Y. Zhang, Q. Su, W. Xu, G. Cao, Y. Wang, A. Pan, S. Liang, A confined replacement synthesis of bismuth nanodots in MOF derived carbon arrays as binder-free anodes for sodium-ion batteries. *Adv. Sci.* **6**, 1900162 (2019).

68. J. Zhou, J. Chen, M. Chen, J. Wang, X. Liu, B. Wei, Z. Wang, J. Li, L. Gu, Q. Zhang, H. Wang, L. Guo, Few-layer bismuthene with anisotropic expansion for high-areal-capacity sodium-ion batteries. *Adv. Mater.* **31**, e1807874 (2019).
